# Cyclic Ruthenium-Peptide Conjugates as Integrin-Targeting Phototherapeutic Prodrugs for the Treatment of Brain Tumors

**DOI:** 10.1021/jacs.3c04855

**Published:** 2023-06-28

**Authors:** Liyan Zhang, Peiyuan Wang, Xue-Quan Zhou, Ludovic Bretin, Xiaolong Zeng, Yurii Husiev, Ehider A. Polanco, Gangyin Zhao, Lukas S. Wijaya, Tarita Biver, Sylvia E. Le Dévédec, Wen Sun, Sylvestre Bonnet

**Affiliations:** † Leiden Institute of Chemistry, Universiteit Leiden, Einsteinweg 55, 2333 CC Leiden, Netherlands; ‡ State Key Laboratory of Fine Chemicals, Dalian University of Technology, 2 Linggong Road, Dalian 116024, P. R. China; § Key Laboratory of Design and Assembly of Functional Nanostructures, Fujian Institute of Research on the Structure of Matter, Chinese Academy of Sciences, Fuzhou 350002, P. R. China; ∥ Leiden Institute of Biology, Universiteit Leiden, Einsteinweg 55, 2333 CC Leiden, Netherlands; ⊥ Leiden Academic Centre for Drug Research, Universiteit Leiden, Einsteinweg 55, 2333 CC Leiden, Netherlands; # Department of Chemistry and Industrial Chemistry, University of Pisa, 56124 Pisa, Italy

## Abstract

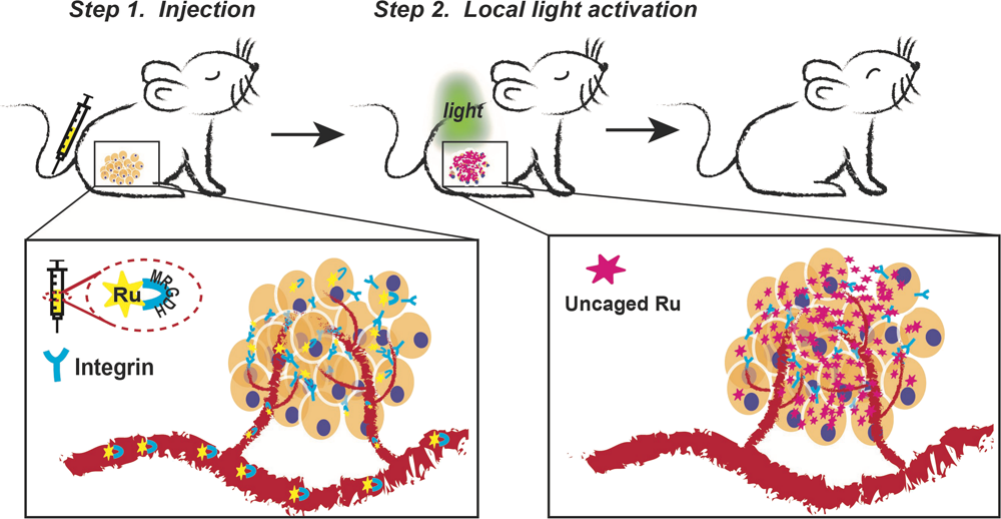

To investigate the potential of tumor-targeting photoactivated chemotherapy, a chiral ruthenium-based anticancer warhead, Λ/Δ-[Ru(Ph_2_phen)_2_(OH_2_)_2_]^2+^, was conjugated to the RGD-containing Ac-MRGDH-NH_2_ peptide by direct coordination of the M and H residues to the metal. This design afforded two diastereoisomers of a cyclic metallopeptide, Λ-[**1**]Cl_2_ and Δ-[**1**]Cl_2_. In the dark, the ruthenium-chelating peptide had a triple action. First, it prevented other biomolecules from coordinating with the metal center. Second, its hydrophilicity made [**1**]Cl_2_ amphiphilic so that it self-assembled in culture medium into nanoparticles. Third, it acted as a tumor-targeting motif by strongly binding to the integrin (*K*_d_ = 0.061 μM for the binding of Λ-[**1**]Cl_2_ to α_IIb_β_3_), which resulted in the receptor-mediated uptake of the conjugate *in vitro*. Phototoxicity studies in two-dimensional (2D) monolayers of A549, U87MG, and PC-3 human cancer cell lines and U87MG three-dimensional (3D) tumor spheroids showed that the two isomers of [**1**]Cl_2_ were strongly phototoxic, with photoindexes up to 17. Mechanistic studies indicated that such phototoxicity was due to a combination of photodynamic therapy (PDT) and photoactivated chemotherapy (PACT) effects, resulting from both reactive oxygen species generation and peptide photosubstitution. Finally, *in vivo* studies in a subcutaneous U87MG glioblastoma mice model showed that [**1**]Cl_2_ efficiently accumulated in the tumor 12 h after injection, where green light irradiation generated a stronger tumoricidal effect than a nontargeted analogue ruthenium complex [**2**]Cl_2_. Considering the absence of systemic toxicity for the treated mice, these results demonstrate the high potential of light-sensitive integrin-targeted ruthenium-based anticancer compounds for the treatment of brain cancer *in vivo*.

## 1 Introduction

Since cisplatin has been approved for the treatment of cancer in clinics in 1978, metallodrugs have become an important line of research in oncology.^1^ However, in the clinics, cisplatin and its derivatives such as oxaliplatin or carboplatin have demonstrated significant side effects for cancer patients; moreover, their treatment efficacy is highly limited because of drug resistance and poor tumor selectivity.^2^ Many studies have focused on the improvement of platinum drugs by developing, for example, prodrugs that are activated by intracellular reduction,^3^ targeted to the tumor by conjugation to cancer-targeting motives,^4^ or based on different metals.^5,6^ Among these alternatives, ruthenium(II)-polypyridine compounds have received much attention because of their appealing photophysical and photochemical properties, making them effective candidates as light-activated prodrugs, for example, for the photodynamic therapy (PDT) treatment of tumors.^7−10^ Other types of ruthenium(II) polypyridyl compounds have more recently been developed as a new form of light-triggered cancer treatment deemed photoactivated chemotherapy (PACT).^11^ In such compounds, the ability of the ruthenium center to bind to biological molecules,^12^ or of the ligand to inhibit a protein,^13^ is temporarily shielded in the dark by the formation of a coordination bond between both moieties, which is referred to as “caging.” Upon light irradiation of the prodrug in the tumor, the ruthenium complex is “uncaged,” i.e., the protecting ligand is released by photosubstitution, which re-establishes the ability of the ligand or the ruthenium center to bind to biomolecules and to kill cancer cells.^14^ While PDT requires dioxygen in the irradiated tissues because it involves energy or electron transfer from a triplet excited state of the ruthenium complex to the O_2_ molecule,^15,16^ in PACT, the photosubstitution reaction does not require O_2_ to occur.^17−19^ This different mode-of-action has triggered several studies toward the application of PACT for the treatment of hypoxic tumors.^17,20,21^ Hypoxic tumors form a class of solid tumors characterized by a low dioxygen concentration, which limits the outcome not only of PDT but also of other anticancer treatments such as radiation therapy.^22,23^ In principle, the PACT strategy lowers the systemic toxicity of ruthenium warheads without jeopardizing their anticancer efficacy and enables them to work effectively even in hypoxic environments.

In PACT, like in PDT, local light activation of the prodrug represents a form of physical tumor targeting, which contributes to lower systemic toxicity compared to traditional chemotherapy.^24^ However, systemic toxicity would be further diminished if the prodrug could also be biologically targeted to the tumor; it would enhance the prodrug tumor delivery efficacy before light activation, and hence allow for lower dosages without sacrificing the antitumor efficacy. Several conjugation strategies have shown great promise in enhancing the pharmacological properties of ruthenium-based therapeutics and PDT compounds.^25^ These strategies include conjugation of the ruthenium complex to biomolecules,^6,25−27^ especially those involved in tumor proliferation.^26−28^ Among these targeted groups, the arginylglycylaspartic acid tripeptide (Arg-Gly-Asp, hereafter RGD) has been particularly studied because of its simplicity and its good targeting properties toward integrins.^29,30^ Integrins form a family of 24 transmembrane heterodimeric glycoproteins assembled by 18 α-subunits and 8 β-subunits, many of which, including α_v_β_1_, α_v_β_3_, α_IIb_β_3_, α_v_β_5_, etc., can be recognized by the RGD sequence.^29,31^ Such integrins are present at the surface of all cells; however, their overexpression in blood vessels during tumor angiogenesis has made them highly attractive as molecular targets in cancer chemotherapy.^32^ Therefore, short peptides containing the RGD motif have been widely applied as cancer-targeting moieties for cancer therapy and diagnosis:^31^ They have been conjugated to coordination complexes,^30^ molecular probes,^33−36^ photosensitizers for PDT,^37,38^ and organometallic ruthenium complexes.^39^ Though simple linear RGD peptides can be used, cyclic (penta)peptides including the RGD motif have higher binding affinity with integrins, thus offering better active targeting properties *in vitro* and *in vivo*.^40^ In most reported metal–RGD conjugates, the peptide is covalently bound to one of the ligands chelated to the metal center, and the sole function of the RGD peptide is to target the complex to integrins at the surface of cancer cells, without a guarantee that internalization of the bioactive agent takes place. This is not an issue for imaging agents, as binding to the surface of cancer cells is sufficient for imaging a tumor. However, for therapeutic agents such as ruthenium-based PACT compounds, it may represent an issue, as the metal warhead needs to penetrate the cells to generate cytotoxicity.

**Scheme 1:**
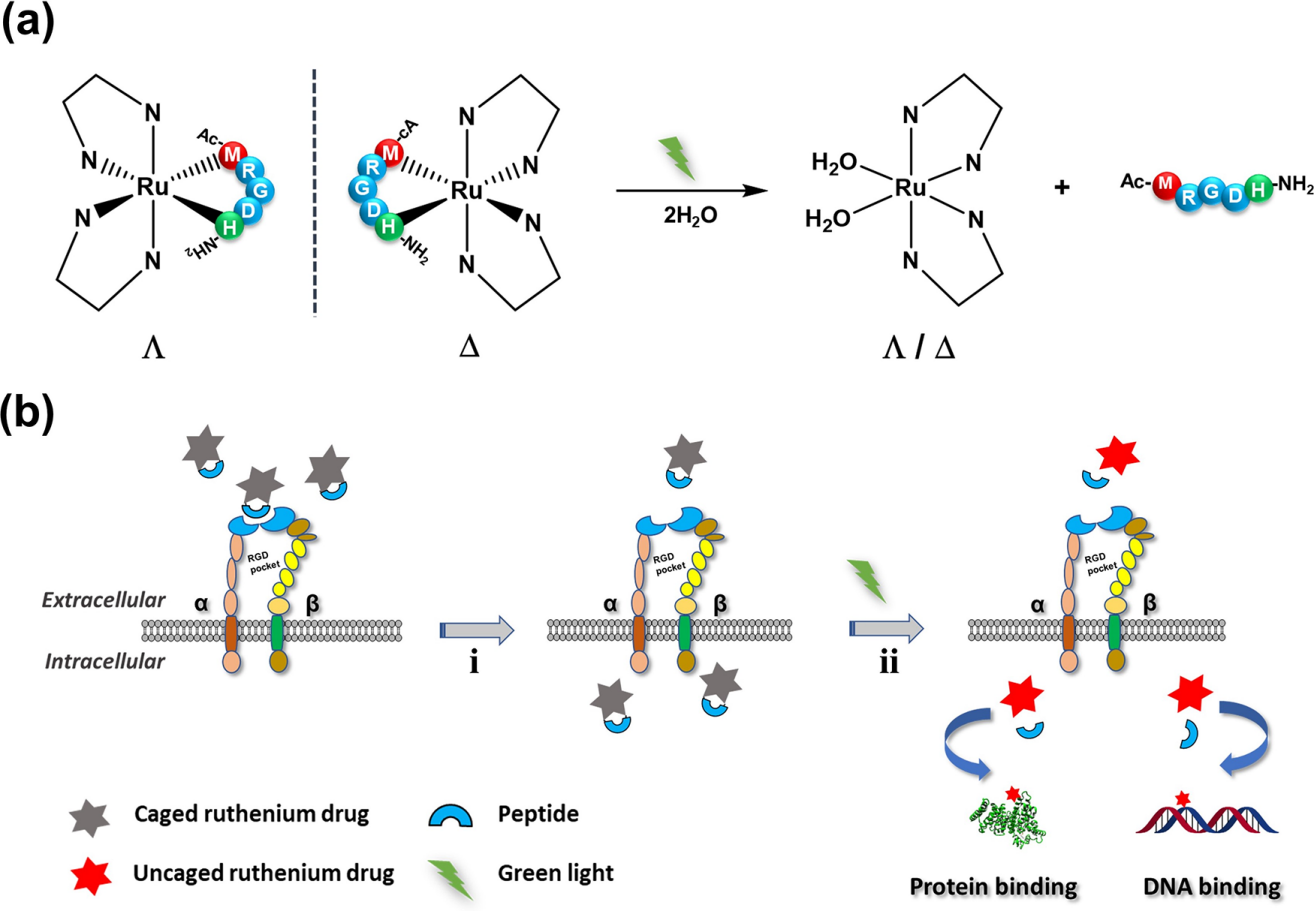
Ru-RGD Conjugates for PACT Anticancer Treatment (a) Photosubstitution of an RGD-containing peptide by green light irradiation from cyclic Ru-MRGDH conjugates in water. (b) Activation of the cytotoxicity from the cyclic Ru-MRGDH conjugate: (i) recognition of the cyclic RGD motif by overexpressed integrins at the surface of cancer cells and subsequent internalization through receptor-mediated uptake and (ii) releasing the toxic ruthenium payload upon light activation.

In this work, we investigated the need for biological tumor targeting in ruthenium-based PACT by synthesizing the pair of diastereoisomeric prodrugs Λ**-** and Δ-[Ru(Ph_2_phen)_2_(κ^S^,κ^N^-(Ac-MRGDH-NH_2_))]Cl_2_ (Λ-**[1]**Cl_2_ and Δ-**[1]**Cl_2_, where Ph_2_phen = 4,7-diphenyl-1,10-phenanthroline). In these prodrugs, the pentapeptide Ac-MRGDH-NH_2_ bears three different functions. First, by direct coordination of its terminal methionine and histidine residues to ruthenium(II), it serves as a photocleavable protecting group for the cytotoxic bis-aqua ruthenium warhead [Ru(Ph_2_phen)_2_(OH_2_)_2_]^2+^ (Scheme 1a). Second, we envisioned that upon coordination with the metal, the peptide would generate a cyclic RGD-ruthenocycle that may bind integrins in a strong manner, thereby targeting the prodrug biologically to the tumor.^35,41^ Third, because of the reported photosubstitution properties of ruthenium polypyridyl complexes, the pentapeptide should be cleaved off upon visible light irradiation, thereby recovering a ruthenium warhead capable of penetrating through the cell membrane and killing the cancer cell.^20,42^ In other words, Λ-**[1]**Cl_2_ and Δ-**[1]**Cl_2_ were designed as integrin-targeted PACT compounds (Scheme 1b). We present the chemical, photochemical, and biological properties of Λ-**[1]**Cl_2_ and Δ-**[1]**Cl_2_*in vitro* and demonstrate their tumor-targeting and antitumor properties *in vivo* using a brain cancer mouse model. We included in our study the known analogous complex *rac*-[Ru(Ph_2_phen)_2_(mtmp)]Cl_2_ (**[2]**Cl_2_, mtmp = 2-methylthiomethylpyridine); it has similar photoreactivity and delivers the same [Ru(Ph_2_phen)_2_(OH_2_)_2_]^2+^ warhead upon photosubstitution of mtmp, but it is, in principle, not biologically targeted to integrins in the dark (Figure S9).

## 2 Results

### 2.1 Synthesis and Characterization

**Figure 1:**
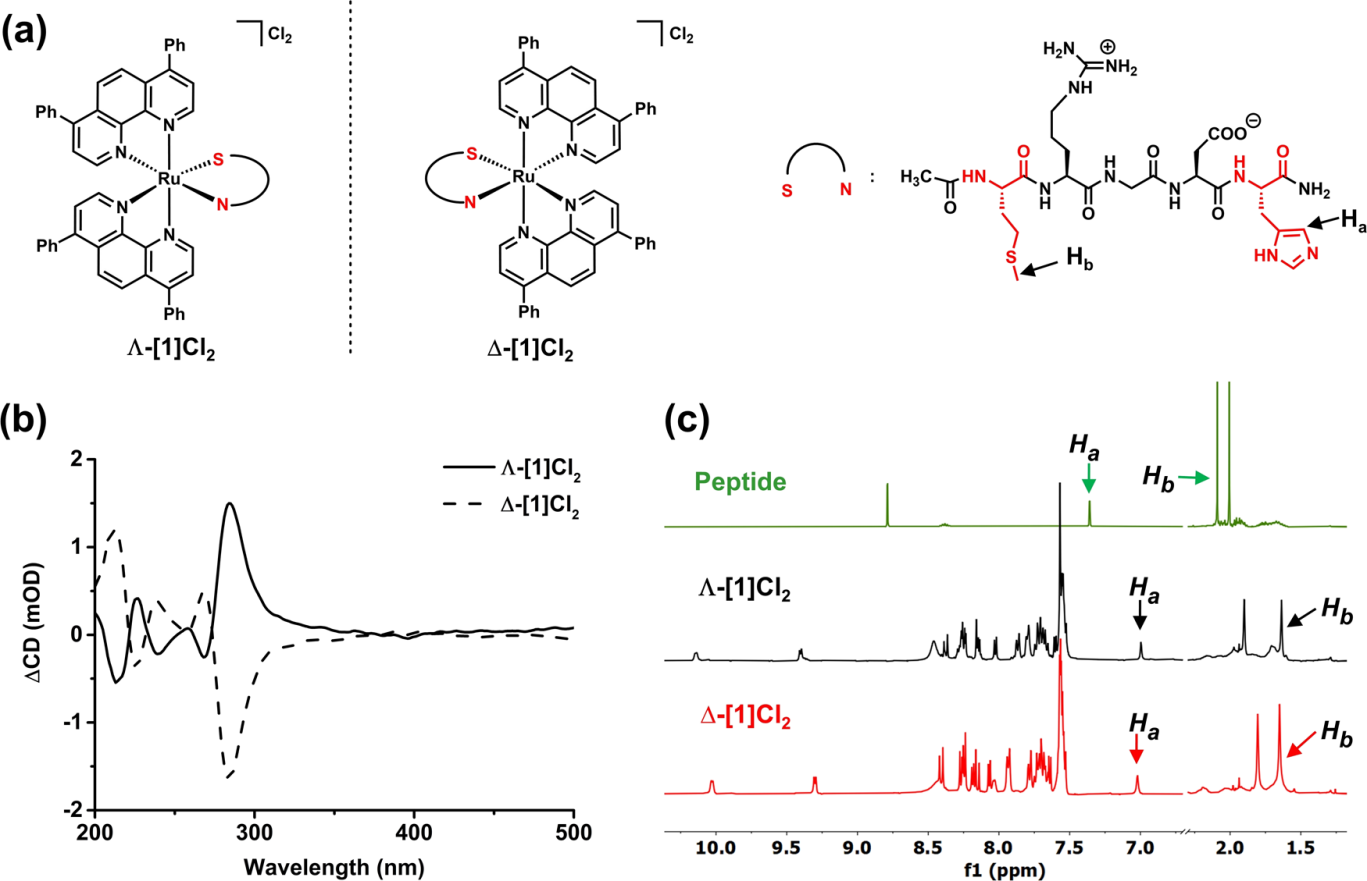
Chemical structures (a) and characterization (b) of the two diastereoisomers of the Ru-RGD conjugate [**1**]Cl_2_. (a) Formulas of Λ-[**1**]Cl_2_, **Δ**-[**1**]Cl_2_, and the enantiomerically pure l-peptide Ac-MRGDH-NH_2_. For the sake of simplicity, the Ac-MRGDH-NH_2_ peptide is further abbreviated as “S–N,” where S symbolizes the coordinating sulfur atom from Met and N is the coordinated imine atom of the imidazole cycle from His. (b) Circular dichroism spectra (0.1 mM, H_2_O) of the purified diastereomers Λ-[**1**]Cl_2_ and Δ-[**1**]Cl_2_. (c) Selected regions of the ^1^H NMR spectra (400 MHz, CD_3_OD) of the free Ac-MRGDH-NH_2_ peptide and the purified diastereomers Λ-[**1**]Cl_2_ and Δ-[**1**]Cl_2_.

The compounds Λ-[**1**]Cl_2_ and Δ-[**1**]Cl_2_ were prepared by refluxing the racemic ruthenium precursor *cis*-[Ru(Ph_2_phen)_2_Cl_2_] and the free Ac-MRGDH-NH_2_ peptide in an ethanol/water 1:1 mixture (pH = 7.5) for three days at 60 °C under N_2_. Due to the enantiomerically pure nature of the peptide and the Δ/Λ chirality of the metal center, it was possible to isolate both diastereomers *via* high-performance liquid chromatography (HPLC) separation (see structures Figure 1a). According to the integral peak area and isolated yields, [**1**]Cl_2_ was obtained for 40% as Λ-[**1**]Cl_2_, and for 60% as Δ-[**1**]Cl_2_ (Figure S10a and the Supporting Information). According to circular dichroism (CD),^43,44^ both diastereoisomers show a nearly symmetrical configuration (Figure 1b), suggesting that most optical transitions involve the chiral metal center (and not the peptide backbone) *via* either π–π* or triplet metal-to-ligand charge transfer (^1^MLCT) transitions. From the aromatic region of the ^1^H NMR spectra, the H_a_ proton from the histidine imidazole ring (see the definition in Figure 1a) was shifted from 7.36 ppm in the free peptide to 6.99 and 7.02 ppm in Λ-[**1**]Cl_2_ and Δ-[**1**]Cl_2_, respectively, while the H_b_ proton from the methionine *S*-methyl group was shifted from 2.09 ppm to 1.63 and 1.65 ppm, respectively (Figure 1c), thereby demonstrating coordination of both terminal amino acid residues to the metal center. Further characterization by high-resolution mass spectrometry (HR-MS), two-dimensional (2D) NMR, and HPLC (Figures S2–S8 and S11a,b) confirmed the formation of a 1:2:1 Ru/Ph_2_phen/peptide conjugate, thereby proving the metallacycle structure of both Ru-peptide conjugates Λ-[**1**]Cl_2_ and Δ-[**1**]Cl_2_.

### 2.2 Photochemistry Studies

**Figure 2:**
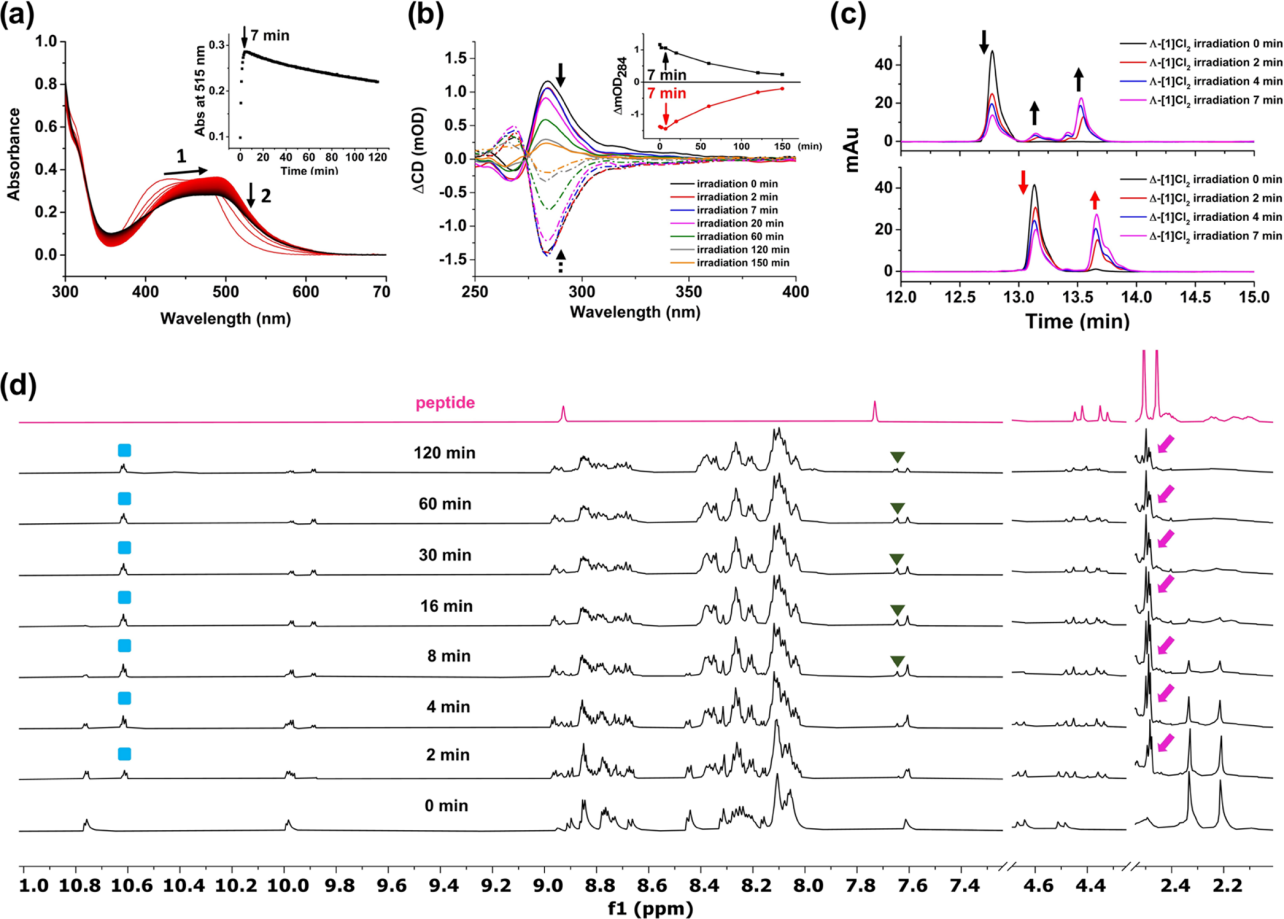
Photochemistry of Ru-RGD conjugates. (a) Evolution of the UV–vis absorption spectra of Δ-[**1**]Cl_2_ (25 μM) upon irradiation with a LED light (515 nm, 4.0 mW/cm^2^) at 25 °C in H_2_O. (b) Time-dependent CD spectra of Λ-[**1**]Cl_2_ and Δ-[**1**]Cl_2_ (67 μM, water) under green light irradiation for 150 min. Inset: Evolution of ΔmOD at 284 nm for Λ-[**1**]Cl_2_ and Δ-[**1**]Cl_2_ under irradiation. (c) HPLC trace of Λ-[**1**]Cl_2_ and Δ-[**1**]Cl_2_ (0.67 mM, water) upon green light irradiation for 7 min. Every HPLC run used the gradient: 10–90% phase B/phase A, 25 min, flow rate = 14 mL/min, UV channel = 290 nm (see the Experimental Section). (d) ^1^H NMR spectra of Δ-[**1**]Cl_2_ in 7:3 v/v acetone-*d*_6_/D_2_O after irradiation with green light (525 nm, 12.6 mW/cm^2^, N_2_) for different times; pink arrow, green triangle, and blue square represent H_b_, H_a_ of the peptide, and H_3_ of Ph_2_phen of the photoproduct, respectively (see Figure S7).

As these conjugates were designed for PACT, we first monitored the time evolution of their UV–vis spectrum and mass spectra in the dark and under light irradiation in pure water and water/acetonitrile 1:1 mixtures. Pure water models, to some extent, represent the aqueous environment experienced by the compound in an *in vitro* assay. On the other hand, H_2_O is a worse ligand for ruthenium(II) than many nucleophiles encountered in a biological environment, such as amino acids or nucleic acids, which results in underestimations of photosubstitution kinetics when pure water is used as a solvent. A water/acetonitrile 1:1 mixture was additionally used to test the photoactivity of the compound, in order to take this effect into account. Dark stability tests in a H_2_O/CH_3_CN (1:1 v/v) mixture demonstrated that both complexes were thermally stable at room temperature in the presence of either H_2_O or CH_3_CN (Figure S15). When the sample was irradiated with green light in pure water, however, the UV–vis spectrum of Δ-[**1**]Cl_2_ (Figure 2a) showed first a red shift and an increase of the broad ^1^MLCT band at 400–500 nm (0–7 min) and then a gradual decrease in intensity (>7 min). In the H_2_O/CH_3_CN (1:1 v/v) mixture (Figure S16c), the initial increase was much faster (<1 min), after which the peak slowly shifted to higher energies. Mass spectrometry of the reaction mixture after irradiation in water showed new peaks at *m*/*z* = 479 (Figure S17a,b), corresponding to [Ru(Ph_2_phen)_2_(η^1^-Ac-MRGDH-NH_2_)(H_2_O)]^3+^ (calcd *m*/*z* for [M]^3+^ = 479.4), as well as at *m*/*z* = 419, corresponding to the final photoproduct [Ru(Ph_2_phen)_2_(H_2_O)_2_]^2+^ plus H_2_O (calcd *m*/*z* = 419.1, Figure S17b). In the presence of acetonitrile, the peaks for Δ-[**1**]Cl_2_ at *m*/*z* = 473.9 and 710.6 were no longer observed after irradiation, and instead, a new peak at *m*/*z* = 423.9 was detected, corresponding to [Ru(Ph_2_phen)_2_(MeCN)_2_]^2+^ (calcd *m*/*z* =424.1, Figure S17c). Altogether these irradiation studies showed that the peptide can be fully photosubstituted by solvent molecules, especially when the solvent contained a large excess of a strong donor (MeCN). For Λ-[**1**]Cl_2_, qualitatively similar photoreactivity was observed (Figure S16a,b), indicating that the photosubstitution reactivity of these complexes is independent of the chirality at the metal center. In this photochemistry study, two individual photosubstitution steps were clearly observed, with ∼7 min as the turning point (in such irradiation conditions). To quantify the efficiency of photochemical peptide cleavage, the two successive photosubstitution quantum yields, Φ_PS1_ and Φ_PS2_, were measured using the time evolution of the absorption spectrum of an irradiated solution for each isomer.^45^ As shown in Figures S18 and S19 and Table S1, Φ_PS1_ and Φ_PS2_ were found to be 0.13 and 0.0007, respectively, in H_2_O for Λ-[**1**]Cl_2_, while higher values were observed (Φ_PS1_ = 0.25 and Φ_PS2_ = 0.0024) in a H_2_O/CH_3_CN 1:1 mixture. In both conditions, the second photosubstitution reaction was found to be much slower than the first one, and no qualitative differences were observed between Λ-[**1**]Cl_2_ and Δ-[**1**]Cl_2_.

We also followed the time evolution of the CD spectrum of Λ-[**1**]Cl_2_ and Δ-[**1**]Cl_2_ under green light irradiation (Figure 2b). Surprisingly, within 7 min there was almost no change of the ΔCD intensity of Δ-[**1**]Cl_2_ at 284 nm, while that of Λ-[**1**]Cl_2_ slightly increased. Upon further light irradiation, the intensity of the CD band of both compounds gradually decreased to zero, which is characteristic of the well-documented racemization of bis- or tris-chelated Ru complexes upon visible light irradiation.^46^ HPLC was further applied to monitor the relative rates of the first ligand dissociation step and of the metal center racemization during the first 7 min of irradiation. As shown in Figure 2c, the retention time of Λ-[**1**]Cl_2_ and Δ-[**1**]Cl_2_ before irradiation were 12.7 and 13.1 min, respectively. Initially (<7 min), both compounds were photoactivated to afford [Ru(Ph_2_phen)_2_(Ac-MRGDH-NH_2_)(H_2_O)]Cl_2_, where we hypothesized that either the Met sulfur atom or the His nitrogen donor was photosubstituted by a water molecule, leading to a cycle opening. At *t* = 7 min (Figure 2c), the photoproducts observed for Λ-[**1**]Cl_2_ and Δ-[**1**]Cl_2_ had different retention times and were concluded to be different species. At this time point, the initial Ru-peptide conjugates had degraded by ∼50% according to HPLC, while the CD spectra barely showed any change (Figure 2b). According to these observations, we hypothesize that 2 photoreactions occurred during light activation: the first step was the cleavage of one coordination bond (according to UV–vis spectroscopy and HPLC), without racemization of the chirality of the Ru core since the CD spectrum showed no change. After 7 min, however, further ligand dissociation occurred, while according to CD, racemization of the metal center also started to occur. The small peak observed at *t* = 13.1 min in a sample of Λ-[**1**]Cl_2_ irradiated for 2 min suggested that a small ratio of Λ-[**1**]Cl_2_ converted to Δ-[**1**]Cl_2_, while the opposite transformation did not occur.

To further confirm our hypotheses on the photosubstitution process, we tracked the evolution of the ^1^H NMR spectrum of Δ-[**1**]Cl_2_ in 7:3 v/v acetone-*d*_6_/D_2_O at different irradiation times (525 nm, 12.6 mW/cm^2^). According to Figure 2d, within the first 8 min, the proton traces at 2.33 and 2.22 ppm, corresponding to the −CH_3_ groups from the *N*-terminal acetyl and methionine residue of the peptide, gradually disappeared, while the new peaks around 2.50 ppm arose (pink arrow), implying methionine was gradually released during irradiation. On the other hand, the proton from imidazole of histidine at 7.60 ppm was essentially retained at *t* = 8 min. According to NMR, upon green light activation of Δ-[**1**]Cl_2_ in H_2_O, the Ru-Met bond was cleaved significantly faster than the Ru-His bond, which is reasonable since thioether ligands are softer than imidazole ligands and can be photosubstituted faster.^42,47,48^

### 2.3 Morphology Studies in Cell-Growing Medium

**Figure 3:**
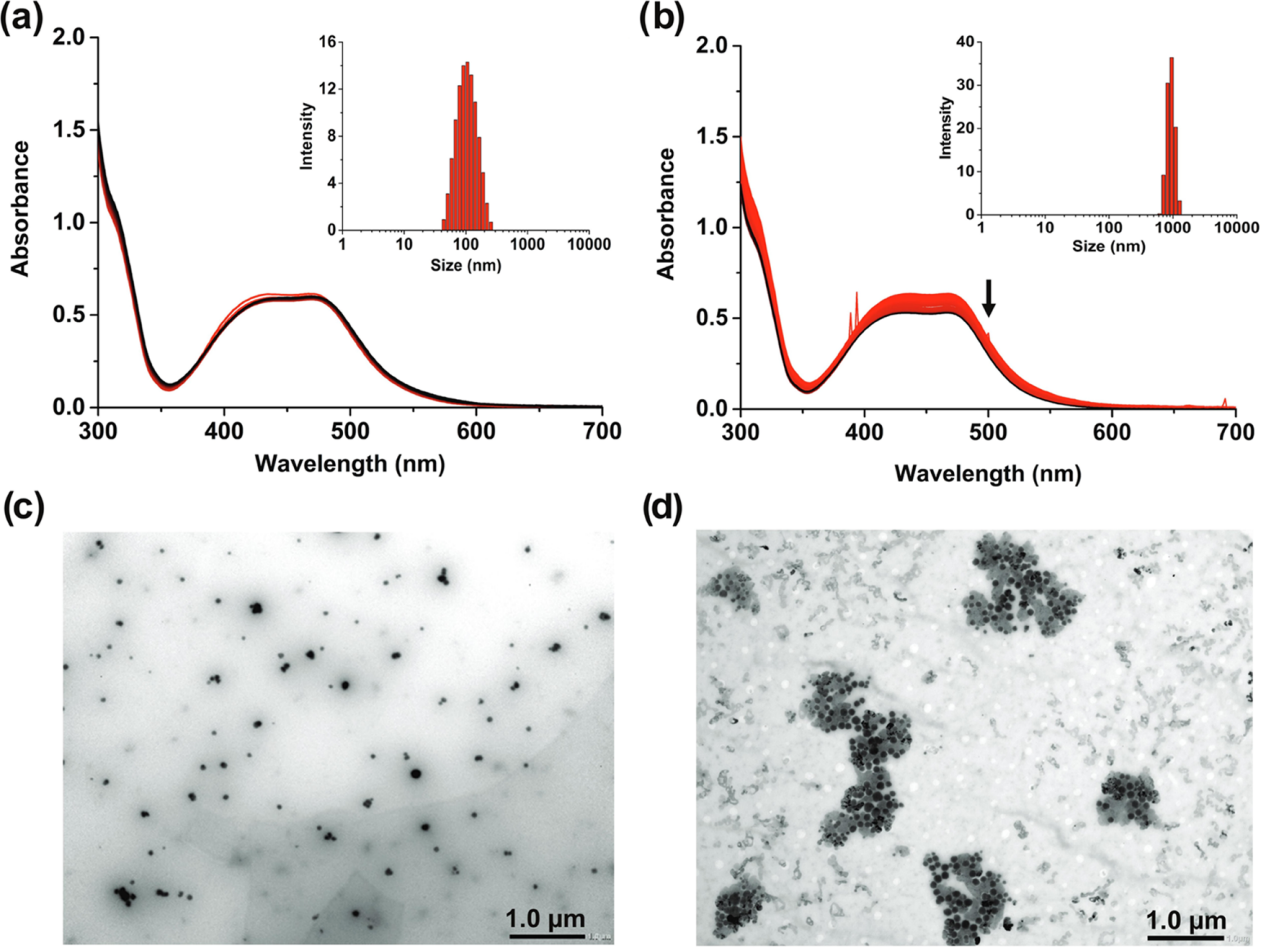
Self-assembly of Ru-RGD conjugates in cell-growing medium. Time-dependent UV–vis absorption spectra and DLS (inset) of Δ-[**1**]Cl_2_ in Opti-MEM with 2.5% FCS (a) or without FCS (b) in the dark (50 μM, 24 h). (c, d) TEM images of Δ-[**1**]Cl_2_ in Opti-MEM with 2.5% FCS (c) or without FCS (d) (50 μM).

Full peptide release upon green light irradiation suggests that [**1**]Cl_2_ can serve as a PACT compound and that it should be tested *in vitro*. However, since the cellular uptake and interaction with the integrin receptors on the cell membrane also depend on the aggregation properties of these compounds, we first studied the self-assembly of both conjugates Λ-[**1**]Cl_2_ and Δ-[**1**]Cl_2_ in the dark in Opti-MEM cell culture medium by dynamic light scattering (DLS) and transmission electron microscopy (TEM). In Opti-MEM containing 2.5% fetal calf serum (FCS), the absorbance spectra of Δ-[**1**]Cl_2_ (Figure 3a) did not show any variation for 24 h, suggesting good dark stability of the conjugate. However, in a medium deprived of FCS (Figure 3b), the intensity of the UV–vis spectrum gradually decreased, suggesting precipitation. DLS analysis (Figure 3a and b, inset) confirmed these hypotheses, as it showed the formation of nanoparticles in the presence of FCS, characterized by a size distribution centered around 100 nm, while in the absence of FCS, only peaks above 1 μm were observed, characteristic of precipitation. TEM images of a solution of Δ-[**1**]Cl_2_ in Opti-MEM containing 2.5% FCS (Figure 3c) clearly showed a homogeneous distribution of well-defined nanoparticles, while large aggregates of these nanoparticles were observed for a solution prepared without FCS (Figure 3d). Nanoparticles of similar size were observed for Λ-[**1**]Cl_2_ but not for [**2**]Cl_2_ (Figure S20a–c), suggesting that the amphiphilic structure of the ruthenium-peptide conjugates, with a polar peptide and an apolar [Ru^II^(Ph_2_phen)_2_] fragment, promotes self-assembly. Such colloid self-assembly seems to be stabilized in solution by FCS, as discussed recently for another metallodrug.^49^ Altogether, this unexpected aggregation suggested that Ru-RGD conjugates such as Λ-[**1**]Cl_2_ and Δ-[**1**]Cl_2_ may represent a new form of drug self-delivery system (DSDS),^50^ a family of small molecular drugs that form their own nanosized drug-delivery system by self-assembly triggered by the physiological environment. This property makes them particularly promising drug candidates, compared to classical small molecules, as DSDS may afford tumor targeting *in vivovia* the enhanced permeability and retention (EPR) effect.^31,51^

### 2.4 Anticancer Studies on 2D Monolayer Cells

As a following step, cytotoxicity studies were performed in 2D monolayers of three human cancer cell lines, i.e., A549 (human adenocarcinoma alveolar basal epithelial cells), U87MG (human primary glioblastoma cells), and PC-3 (human prostate cancer cells). Hypoxia is a common characteristic of solid tumors and comes as a consequence of the high consumption of oxygen by rapidly dividing cells, coupled to suboptimal blood vessel growth and resulting in deficient oxygen delivery.^22^ Hypoxia has been reported to be associated with different kinds of resistance to anticancer agents, especially PDT drugs,^52^ while PACT compounds are notoriously known for their light activation mechanism to remain efficient in hypoxic cancer cells.^53^ Efficacy studies in hypoxic conditions are hence important for any light-activated drugs, and the *in vitro* cytotoxicity studies were realized here under both normoxic (21% O_2_) and hypoxic (1% O_2_) conditions. In these first cytotoxicity studies, after 24 h dark incubation with the compound, cells were either directly irradiated with green light (λ_irr_ = 520 nm, 20 min, 13.1 J/cm^2^) or left in the dark. A sulforhodamine B (SRB) endpoint viability assay was realized after 48 h of further incubation (*t* = 96 h). Half-maximal effective concentrations (EC_50_ in μM), defined as the concentration capable of killing half of the cancer cells compared to the untreated control, and the photoindex (PI), defined as EC_50,dark_/EC_50,light_, were then determined to characterize the cytotoxicity and light activation of the Ru-peptide conjugate. The dose–response curves and the corresponding EC_50_ values are shown in Figure S21 and Table 1, respectively. Cisplatin was included in this study as a prototypical cytotoxic chemotherapy metallodrug. In normoxic conditions, both diastereomers showed EC_50,dark_ values between 39.5 and 72.5 μM depending on the cell line, while upon green light activation, EC_50,light_ decreased to 3.6 to 6.0 μM, resulting in PI values of 13 and 17 in A549 cells, 12 and 11 in U87MG cells, and 12 and 9 in PC-3 cells for Λ-[**1**]Cl_2_ and Δ-[**1**]Cl_2_, respectively. The EC_50_ values measured after light irradiation were comparable to those measured for cisplatin, which is considered a highly cytotoxic species. On the other hand, in the dark, Λ-[**1**]Cl_2_ and Δ-[**1**]Cl_2_ were much less toxic than cisplatin, which highlights the potential for reduced side effects of such compounds in the nonirradiated area. In hypoxic conditions, the EC_50,dark_ values of Λ-[**1**]Cl_2_ and Δ-[**1**]Cl_2_ were found to be similar to those in normoxia, while the EC_50,light_ values in, for example, the A549 cells, were unexpectedly 3 times higher, i.e., 19 and 17 μM, respectively, resulting in overall lower photoindex values of 3. Similar trends were also observed in hypoxic U87MG cells, especially in the hypoxic PC-3 cell line in which cytotoxicity was very mild in the hypoxic light-activated group. Lower phototoxicity of light-activated drugs under hypoxia can be a sign either that the phototoxicity under normoxia involves some form of a photodynamic effect, or that the hypoxic cells are more difficult to kill than normoxic cells, as hypoxia triggers a range of resistance effects.^22^

**Table 1:** Cytotoxicity of Ru-RGD Conjugates^a^,^b^,^c^,^d^

		Λ-[1]Cl_2_					Δ-[1]Cl_2_					cisplatin			
cell lines	% O_2_	EC_50,dark_ (μM)	CI_95_ (μM)	EC_50,light_ (μM)	CI_95_ (μM)	PI	EC_50,dark_ (μM)	CI_95_ (μM)	EC_50,light_ (μM)	CI_95_ (μM)	PI	EC_50,dark_ (μM)	CI_95_ (μM)	EC_50,light_ (μM)	CI_95_ (μM)
A549	21	66.8	–7.9	5.0	–0.4	**13**	72.5	-	4.3	–0.2	**17**	2.3	–0.2	2.4	–0.3
			+5.5		+0.4			+48.2		+0.2			+0.3		+0.3
	1	51.0	–2.4	19.0	–2.4	**2.7**	50.1	-	17.3	–3.9	**2.9**	6.5	–1.5	6.3	–1.3
			+3.0		+2.1			+		+3.1			+2.0		+1.7
U87MG	21	41.6	–8.9	3.8	–0.8	**12**	39.5	–8.6	3.6	–0.4	**11**	2.8	–0.4	4.0	–0.5
			+16		+0.8			+6.6		+0.3			+0.4		+0.6
	1	41.4	–1.7	19.0	–1.5	**2.2**	42.7	–5.0	18.8	–0.9	**2.3**	13.0	–1.2	12.5	–1.1
			+6.6		+1.7			+7.0		+1.0			+1.3		+1.2
PC-3	21	66.6	-	5.3	–1.1	**12**	52.0	-	6.0	–1.3	**9**	4.5	–0.8	4.1	–0.9
			+>50		+1.5			+		+1.9			+1.0		+1.1
	1	74.3	-	46.5	–6.8	**1.5**	61.4	-	50.7	–7.1	**1.2**	23.1	–3.8	25.8	–3.2
			+>50		+11			+>50		+13			+4.9		+3.8
U87MG spheroids	21	37.0	–4.4	7.6	–1.3	**4.9**	46.0	–3.1	10.9	–2.2	**4.2**	8.6	–2.9	7.9	–2.2
			+4.5		+1.4			+3.3		+2.6			+3.9		+2.7

a Half-maximal effective concentrations (EC_50_ in μM, *n* = 3) and 95% confidence intervals (CI_95_ in μM) for Λ-[**1**]Cl_2_, Δ-[**1**]Cl_2_, and cisplatin in the dark or upon green light irradiation in 2D monolayers of A549, U87MG, and PC-3 cell lines under normoxic (21% O_2_) and hypoxic (1% O_2_) conditions and three-dimensional (3D) spheroids of U87MG under normoxia (21%).

b PI = photoindexes, defined as (EC_50,dark_/EC_50,light_).

c Irradiation condition: normoxia 520 nm, 10.9 mW/cm^2^, 13.1 J/cm^2^, 20 min; hypoxia 520 nm, 7.22 mW/cm^2^, 13.1 J/cm^2^, 30 min.

d Cancer cells were treated for 24 h in the dark and were not washed before or after irradiation.

### 2.5 Anticancer Study on 3D Multicellular U87MG Spheroids

Although the prodrugs seemed to work on all three cell lines, we decided to pursue our biological investigations on the brain cancer cell line U87MG. Glioblastoma is one of the most aggressive cancers that begins within the brain, with fewer than 5–10% of patients surviving 5 years after diagnosis.^54^ Several clinical studies (e.g., NCT04391062, NCT05363826) are currently ongoing using clinically approved PDT photosensitizers, which suggests that light-activated therapies may be used in the near future to improve the survival of brain cancer patients. Hence, three-dimensional (3D) U87MG tumor spheroids were grown for further testing of the photocytotoxicity of the ruthenium-peptide conjugates. Compared to 2D, 3D multicellular tumor spheroid models provide a more accurate model for the physical penetration of nanoparticles, light, and dioxygen inside a real tumor.^49^ The cytotoxicity of Λ-[**1**]Cl_2_ and Δ-[**1**]Cl_2_ was measured by incubating and irradiating large U87MG glioblastoma spheroids (diameter ∼500 nm) in identical conditions and with the same timeline as for the 2D assays. Phase-contrast brightfield imaging microscopy was used to monitor the spheroid diameter (Figure S22), while the viability of the cells in the spheroids was assayed at the end of the experiment (96 h) using CellTiter-Glo 3D endpoint ATP quantification (Figure S23, Table 1).^55^ The EC_50,dark_ values of Λ-[**1**]Cl_2_ and Δ-[**1**]Cl_2_ toward U87 3D tumor spheroids were 37 ± 4.4 and 46 ± 3.1 μM, respectively, which were similar to the ones found in 2D cell monolayers. Upon light irradiation, the EC_50_ values of Λ-[**1**]Cl_2_ and Δ-[**1**]Cl_2_ decreased to 7.6 ± 1.3 and 10.9 ± 2.2 μM, giving PI values of 4.9 and 4.2 for Λ-[**1**]Cl_2_ and Δ-[**1**]Cl_2_, respectively, showing efficient photoactivation in such conditions. Although the position of these conjugates cannot be determined easily in the spheroids because of their poor emission properties (Figure S24a), their good PI and micromolar cytotoxicity upon light activation allows us to hypothesize that they are able to penetrate well into tumor spheroids. Direct observation of the spheroids showed that the two conjugates seemed to destroy spheroids in a different manner than cisplatin (Figure S22). While cisplatin decreased the size of the spheroids at concentrations down to 60 μM, the ruthenium conjugates completely eliminated the spheroid at high concentrations, and at lower concentrations (20 μM) in the light-irradiated group. Interestingly, at lower concentrations of the light group (5–10 μM, Figure S22, red arrow), the spheroids broke into several pieces. Considering the integrin targeting of these cyclic RGD conjugates, it is speculated that with increasing complex dose, [**1**]Cl_2_ in the dark may interact with the cell–cell aggregation mechanism, while upon light activation, ruthenium separates from the peptide and kills the cancer cells.

### 2.6 Light-Activated Cell Death Mechanism

**Figure 4:**
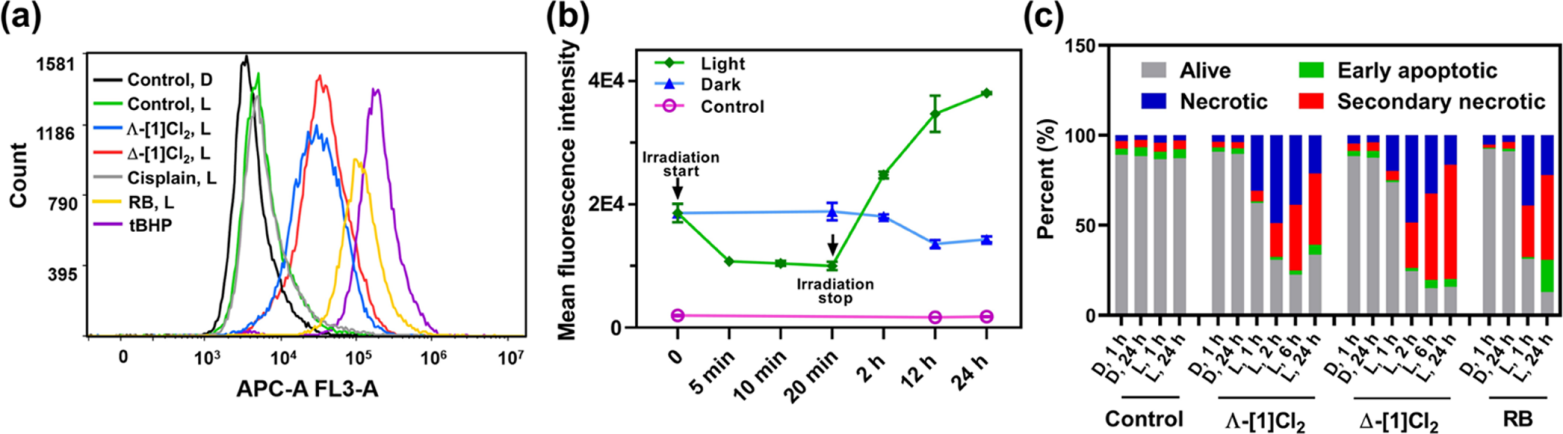
Light-activated cell death. (a) Histograms of reactive oxygen species generation in U87MG cells according to FACS analysis using the CellROX Deep Red Reagent as the ROS probe, after treatment with medium (control), Λ-[**1**]Cl_2_ or Δ-[**1**]Cl_2_, cisplatin, Rose Bengal, or [**2**]Cl_2_ (15 μM, 24 h) in the dark (D) or after light irradiation (L, 515 nm, 13.1 J/cm^2^). tBHP (250 μM) was used as a positive control for ROS generation. The x-axis represents the ROS probe intensity detected by the APC-A channel on the FACS machine; higher values mean higher ROS generation. The corresponding histogram of the dark group and the mean fluorescence intensity of every group are shown in Figure S25 and Table S3. (b) Mean fluorescence intensity of cells (error = standard derivation (SD), *n* = 3) detected by flow cytometry (PC5.5 channel, λ_ex_ = 480 nm, λ_em_ = 650/50 nm) after treatment with Δ-[**1**]Cl_2_ (6 h, 10 μM), medium replacement with drug-free medium, and either irradiation with a green light or no irradiation. Cells treated with medium were taken as the control. Histograms of the mean fluorescence intensity of every group are shown in Figure S26. (c) Percentage of alive (Apop-/DCS1−), early apoptotic (Apop+/DCS1−), necrotic (Apop-/DCS1+), and secondary necrotic (Apop+/DCS1+) U87MG cells quantified by flow cytometry using the Apopxin Deep Red Indicator (apoptosis) and Nuclear Green DCS1 (necrosis) double-staining protocol after treatment with Λ-[**1**]Cl_2_, Δ-[**1**]Cl_2_, or Rose Bengal (20 μM) in the dark (D) or 1, 2, 6, or 24 h after green light irradiation (L, 515 nm, 13.1 J/cm^2^).

The light-induced cell death observed in 2D and 3D cell models encouraged us to further explore the mode-of-action of the two ruthenium-peptide conjugates. Two typical characteristics of photoactive molecules are singlet oxygen (^1^O_2_) generation quantum yields (Φ_Δ_, Figure S24b, Table S2)^56^ and their ability to generate intracellular reactive oxygen species (ROS), which can be measured spectroscopically using the NIR emission of ^1^O_2_, and in cells using the CellROX deep red reagent (Figure S25, Table S3).^57^ Both isomers showed comparatively low but non-zero Φ_Δ_ values (0.046 and 0.059 for Λ-[**1**]Cl_2_ and Δ-[**1**]Cl_2_, respectively), suggesting that a PDT type II mechanism can hardly explain the phototoxicity observed in normoxic conditions. Unexpectedly, however, the intracellular ROS generation assay in U87MG cell lines showed that both isomers generated non-negligible amounts of ROS upon green light activation, with a light/dark ROS ratio of 7.03 and 8.24, respectively (Figure 4a, Table S3). These values were lower compared to that obtained with the prototypical PDT type II agent Rose Bengal (24.0), but they still represented a noticeable level of ROS production, in particular, compared to the negative control cisplatin. To explain this result, we hypothesized that the photoproduct of [**1**]Cl_2_, i.e., the bis-aqua ruthenium complex [Ru(Ph_2_phen)_2_(H_2_O)_2_]^2+^, was able to bind to proteins or nucleic acids and subsequently generate ROS. As shown in Figure 4b, we tracked the ruthenium-based emission (λ_ex_ = 480 nm, λ_em_ = 600–700 nm) of cells treated with Δ-[**1**]Cl_2_, washed with drug-free medium, and either irradiated with a green light or left in the dark. Decreased emission was observed 5 min after the start of light activation, suggesting photosubstitution in the cells. Emission remained low up until the end of the irradiation period (20 min), which we interpret as a dynamic steady-state with interconversion between [Ru(Ph_2_phen)_2_(H_2_O)_2_]^2+^ and/or several [Ru(Ph_2_phen)_2_(protein/DNA)_1/2_]^*n*+^ species. After light irradiation was stopped and the cells were put back in the incubator for 2, 12, and 24 h, a gradually increased emission was observed with increasing incubation time, suggesting that the interaction between [Ru(Ph_2_phen)_2_(H_2_O)_2_]^2+^ and biomolecules generated phosphorescent ruthenium species. As phosphorescence in ruthenium polypyridyl complexes typically originates from ^3^MLCT excited states that are also capable of generating ^1^O_2_, these experiments suggested that these secondary photoproducts may be also capable, under prolonged light irradiation, to generate the ROS observed in Figure 4a. Thus, we concluded that in normoxic cancer cells, both isomers Λ-[**1**]Cl_2_ and Δ-[**1**]Cl_2_ were able to be photosubstituted and subsequently generate ROS upon irradiation, while such ROS generation does not come from the prodrug [**1**]Cl_2_ itself, but from its photoproduct, i.e., [Ru(Ph_2_phen)_2_(protein/DNA)_1/2_]^*n*+^. Overall, Λ-[**1**]Cl_2_ and Δ-[**1**]Cl_2_ carry at the same time both PACT and PDT characteristics.

To see how cells reacted to photoactivation of the prodrug, a further study of the cell death mode was conducted in 2D U87MG cells. In this experiment, the cells were incubated with each complex for 24 h, irradiated or not with green light, and further incubated in the dark for 1, 2, 6, and 24 h, finally using an Apopxin Deep Red Indicator (for apoptosis) and Nuclear Green DCS1 (for necrosis) double-staining protocol to distinguish apoptotic from necrotic cell death. Cisplatin and Rose Bengal were also included for comparison (1 or 24 h). As shown in Figures 4c and S27–S28, in a very short time after light activation, i.e., 1 h, cells treated with Λ-[**1**]Cl_2_ and Δ-[**1**]Cl_2_ died mainly *via* necrosis (Apop–/DCS1+) in the light group, which was similar to cells treated with Rose Bengal and light. Meanwhile, control cells treated with cisplatin mainly died *via* apoptosis (Apop+/DCS1−). When cells were collected and analyzed at 2, 6, or 24 h after light activation, cells treated with [**1**]Cl_2_ became positive for both markers (Apop+/DCS1+), showing a transformation from necrosis to secondary necrosis. This transformation is typical for dead cells. For cells treated with ruthenium, much more cell death was observed 24 h after light irradiation, compared to the dark group. Overall, U87 cells treated with [**1**]Cl_2_ and light clearly died by necrosis very quickly (starting 1 h after irradiation). No significant difference was found between the two isomers of [**1**]Cl_2_.

### 2.7 Receptor-Mediated Cell Uptake

**Figure 5:**
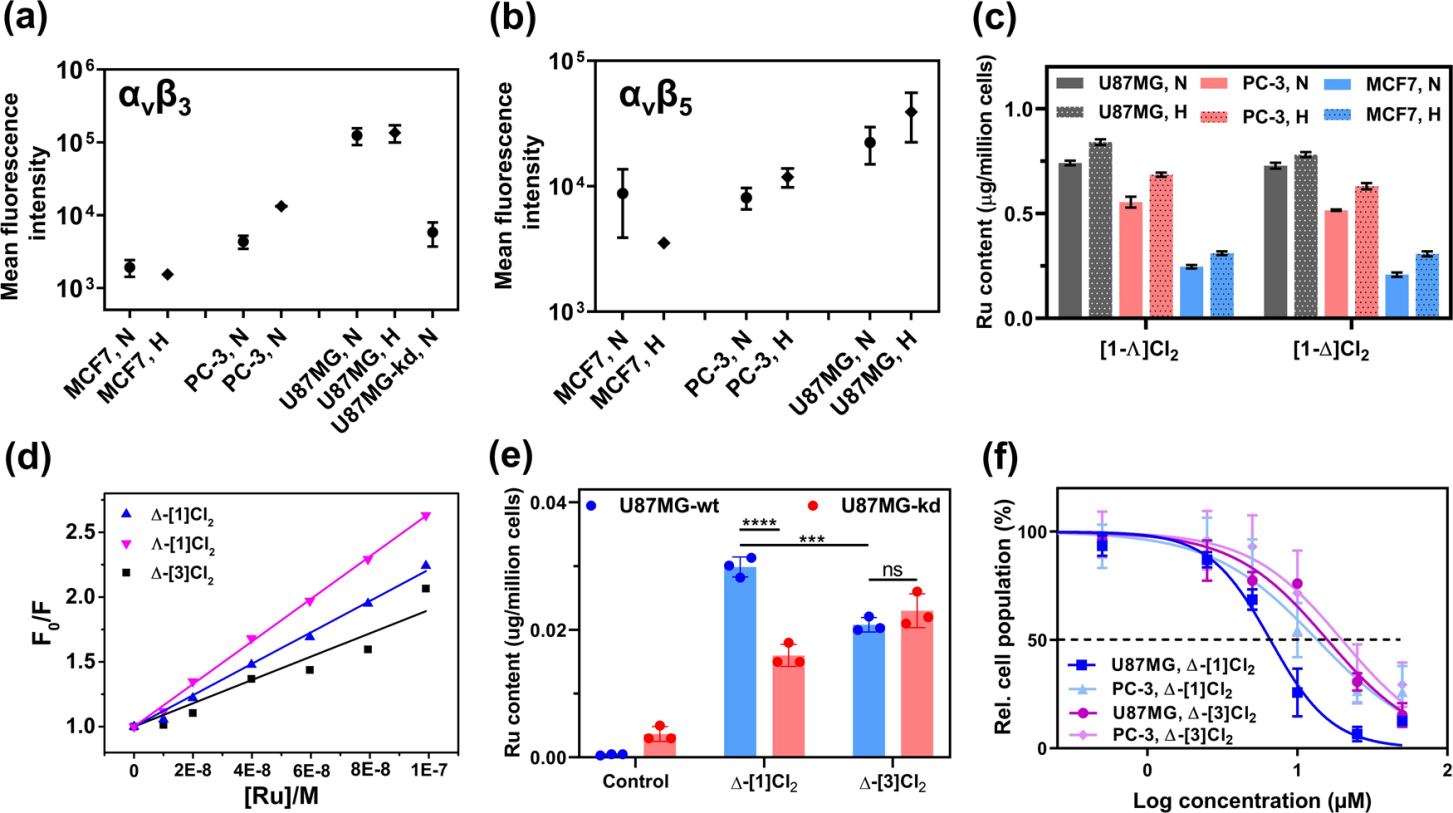
Receptor-mediated cell uptake. The expression of a_V_β_3_ (a) and a_V_β_5_ (b) integrin in U87MG, PC-3, MCF7, and ITGAV knockdown U87MG cell lines. Cell groups (N: normoxia, H: hypoxia) represent the fluorescence intensity of the cells incubated with either anti-integrin α_V_β_3_ or α_V_β_5_ monoclonal antibodies, followed by a secondary antibody conjugated to Alexa-Fluor 488. Error represents the standard deviation (SD) from duplicate experiments. The representative flow cytometry histogram can be found in Figures S29 and S30. (c) Intracellular Ru content (μg/million cells) of U87MG, PC-3, or MCF7 cells after exposure to complexes Λ-[**1**]Cl_2_ or Δ-[**1**]Cl_2_ (12.5 μM, dark, 6 h) in normoxia (N, 21% O_2_) or hypoxia (H, 1% O_2_). Error represents the standard error of the mean (SEM) from triplicate wells. (d) Fitting curve of the Stern–Volmer plots (*F*_0_/*F* vs [Ru]) for the quenching of the emission of integrin α_IIb_β_3_ titrated with complexes Λ-[**1**]Cl_2_, Δ-[**1**]Cl_2_, and Δ-[**3**]Cl_2_ (see raw data in Figure S31). (e) Intracellular Ru content (μg/million cells) of wild-type U87MG (U87MG-wt) and ITGAV knockdown U87MG (U87MG-kd) cells after exposure to complexes Δ-[**1**]Cl_2_ or Δ-[**3**]Cl_2_ (10 μM, dark, 2 h) in normoxia. Error represents SD, *n* = 3. (f) Dose–response curves for U87MG and PC-3 cell lines incubated with Δ-[**1**]Cl_2_ or Δ-[**3**]Cl_2_ for 6 h, washed with fresh medium, and irradiated with green light (520 nm, 13.1 J/cm^2^). Response curves in the dark can be found in Figure S33; error bars represent 95% confidence intervals, *n* = 3.

The U87MG glioblastoma cell line was reported to have a higher integrin expression level^58^ compared to PC-3 cells MCF7.^59,60^ To figure out the relation between the RGD-related integrin expression level in these three cell lines and ruthenium prodrug uptake, the integrin expression level was measured experimentally by a reported double-immunofluorescence protocol.^61^ For this study we checked two typical RGD-targeted integrin subunits, i.e., α_v_β_3_ and α_v_β_5_, and looked at their expression level at the surface of U87MG, PC-3, and MCF7 cells both in normoxic and hypoxic conditions. To ensure full adaptation to such conditions, each cell line was cultured under 21% O_2_ or 1% O_2_ for one month. Cells only incubated with the secondary antibody were included as a negative control (Figure 5a,b). First, as expected, within this series of conditions, the U87MG cell line showed the highest expression for both integrin heterodimers. Whether in normoxia or hypoxia, both MCF7 cells showed hardly any fluorescence intensity, compared with the vehicle control, indicating low integrin α_v_β_3_ and α_v_β_5_ expressions. For PC-3, low integrin α_v_β_3_ and α_v_β_5_ expressions were also observed in normoxic cells, but relatively higher levels were found in hypoxic cells. Hypoxic cells have been reported to upregulate many cellular processes,^31^ and it is interesting to see that a higher expression of integrin was observed for some of the cell lines, offering a new perspective to overcome the drug resistance induced by hypoxia.

In a second step, intracellular Ru accumulation was measured using inductively coupled plasma mass spectrometry (ICP-MS) in the three cell lines for the two different O_2_ concentrations. The uptake study was accomplished in 2D cell monolayers grown in 96-well plates to be better compared with cytotoxicity studies (Figure 5c and Table S4). In normoxic conditions, for [**1**]Cl_2_, U87MG showed the highest ruthenium accumulation (0.74 ± 0.01 μg Ru/million cells), while the difference between the two diastereoisomers was not significant. Ruthenium accumulation was found to be the lowest in MCF7, with about 1/3 of the ruthenium content compared to U87MG, while PC-3 stood in between. These results agreed with the integrin expression study (Figure 5a,b); higher integrin expression in glioblastoma U87MG cells is associated with a higher accumulation of the Ru-RGD conjugates and lower integrin expression with lower uptake. Under hypoxia, all three cell lines showed slightly higher ruthenium uptake, compared to normoxic conditions (Figure 5c, Table S4), but the trends were similar to normoxic conditions, with a ruthenium cellular accumulation decreasing following the series U87MG > PC-3 > MCF7. Overall, the higher cellular uptake of the prodrug was found in the cell lines expressing higher levels of integrin, which strongly suggests that the receptor-mediated uptake of the compounds is taking place and that [**1**]Cl_2_ indeed targets the a_V_β_3_ and a_V_β_5_ integrin present at the surface of the cells.

Since the ruthenium complexes were designed to target integrins, their interaction with the protein should be a key process for recognition, uptake, and toxicity. To check whether the ruthenium-coordinated peptide still retained the interaction binding affinity with the targeted integrin, the association constant (*K*_a_) of both isomers of [**1**]Cl_2_ to integrin α_IIb_β_3_ was measured using luminescence spectroscopy. In this assay, the emission intensity from aromatic residues (tryptophan, tyrosine, and phenylalanine) in integrin α_IIb_β_3_ (λ_emi_ = 345 nm) was monitored upon the addition of different concentrations of the ruthenium-peptide conjugate. The corresponding Stern–Volmer plot shows the evolution of *F*_0_/*F* vs ruthenium-peptide conjugate concentration ([Ru]) (Figure 5d). Here, the [Ru] concentration is the free amount of quencher (i.e., total bound) and is iteratively calculated. This definition means that in the first step [Ru] = C_Ru_ (total amount) is set, and the slope of the plot corresponds to an approximated evaluation of the reciprocal of the binding constant (1/*K*_d_),^33,62^ which is used to calculate the *K*_d_ value. As Gly-Ala replacement in RGD has been reported to induce lower integrin affinity.^63,64^ Without affecting the chemical properties of the peptide, the analogue complex [Ru(Ph_2_phen)_2_(κ^S^,κ^N^-Ac-MRADH-NH_2_)]Cl_2_ (Λ-[**3**]Cl_2_ and Δ-[**3**]Cl_2_) was synthesized (see the Supporting Information, Figures S9–S14), and Δ-[**3**]Cl_2_ was involved as a representative comparison with [**1**]Cl_2_ in the following studies because it was obtained in a higher yield. In the protein-binding assay (Figure 5d), Λ-[**1**]Cl_2_ showed the highest binding affinity with integrin α_IIb_β_3_ (*K*_d_ = 0.061 ± 0.003 μM), followed by Δ-[**1**]Cl_2_ (*K*_d_ = 0.083 ± 0.001 μM) and finally Δ-[**3**]Cl_2_ (*K*_d_ = 0.112 ± 0.009 μM, see Table S5 and associated spectra in Figure S31). All 3 constants had the same order of magnitude (∼0.1 μM), which was comparable to a reported Ru-RGD conjugate (dissociation constants *K*_d_ = 0.25 ± 0.29 μM, binding with integrin α_IIb_β_3_).^33^ The free linear RGD peptide has been reported with *K*_d_ = 1.7 μM for the same integrin heterodimers, as well as a *K*_d_ = 0.03 μM for a natural ligand fibrinogen.^65^ According to these experiments, the interaction affinity with integrin α_IIb_β_3_ of the RGD fragment in Ac-MRGDH-NH_2_ remained upon coordination to Ru and cyclization. When Gly from RGD in [**1**]Cl_2_ was replaced by Ala, the association constant of the ruthenium-peptide conjugate decreased, but the Δ-[**3**]Cl_2_ conjugate retained significant binding affinity with integrin α_IIb_β_3_. There seems, therefore, to exist some nonspecific interaction between the metallacycle and the protein, probably *via* π–π stacking and/or electrostatics, considering the large aromatic rings and the positive charge of the ruthenium complex.

In a more biological context, if the ruthenium-peptide prodrug targets integrins, then cells showing lower integrin expression should be less sensitive to the drug. A cellular ruthenium uptake study was hence realized through wild-type U87MG cells (U87MG-wt) and ITGAV (integrin α_v_) knockdown U87MG cells (U87MG-kd), which have lower α_v_β_3_ expression compared with U87MG-wt cells (Figure 5a). After incubating the cells with Δ-[**1**]Cl_2_ and Δ-[**3**]Cl_2_ for a short period (2 h), the uptake amount of the two compounds by cells was determined by subsequent ICP-MS. First, compared to Δ-[**1**]Cl_2_, the cellular uptake of Δ-[**3**]Cl_2_ toward the U87MG-wt cell line was found to be significantly lower (Figure 5e), which corresponds well with the protein interaction study. Second, the uptake amount of Δ-[**1**]Cl_2_ in U87MG-kd cell lines was shown to be significantly decreased compared to U87MG-wt cells, confirming that the decreased expression of integrin α_v_ has a significant influence on the drugs’ uptake. For Δ-[**3**]Cl_2_, the compounds in two cell lines were taken up in similar amounts. Next to receptor binding, passive diffusion through the membrane can also play a role in the uptake of ruthenium-peptide conjugates.^66^ The octanol–water partition coefficients (log *P*) were hence measured for all three compounds, which showed that the Gly-Ala replacement had a benign influence on the lipophilicity of the conjugate (Figure S32); the log (*P*) values of Λ-[**1**]Cl_2_, Δ-[**1**]Cl_2_, and Δ-[**3**]Cl_2_ were −0.03 ± 0.02, 0.00 ± 0.02, and 0.02 ± 0.05, respectively. Such values indicated that the passive uptake of all three compounds should be comparable. As a result, the lower cellular uptake of Δ-[**3**]Cl_2_ in U87MG-wt cells, compared with Δ-[**1**]Cl_2_, was not a consequence of a difference in lipophilicity; instead, it demonstrated, combined with the lower protein-binding affinity of Δ-[**3**]Cl_2_ with the integrin, that the cellular uptake of Δ-[**1**]Cl_2_ was receptor-dependent. In other words, *in vitro* Δ-[**1**]Cl_2_ indeed targets integrins at the surface of the U87MG-wt cells.

The cytotoxicity of Δ-[**1**]Cl_2_ and Δ-[**3**]Cl_2_ against U87MG and PC-3 cells was further tested in normoxic conditions using a 6 h drug-to-light interval and a washing step with drug-free medium directly prior to light activation. With such a protocol, only the compounds inside the cells or effectively bound to the cell surface before medium refreshment, are responsible for the cytotoxicity. The dose–response curves in the dark and after green light irradiation following this new protocol are shown in Figure S33. In the irradiated group (Figure 5f), the lowest EC_50,light_ value was observed in the U87MG cell line treated with Δ-[**1**]Cl_2_. Higher (and similar) EC_50,light_ values (Table S6) were observed in PC-3 cells treated with Δ-[**1**]Cl_2_ and in both cell types treated with Δ-[**3**]Cl_2_. Altogether, all *in vitro* data demonstrate that Δ-[**1**]Cl_2_ targets U87MG cells better than U87MG-kd or PC-3 cell lines, and that the uptake of [**1**]Cl_2_ in cancer cells is receptor-mediated.

### 2.8 Antitumor Study *In Vivo*

**Figure 6:**
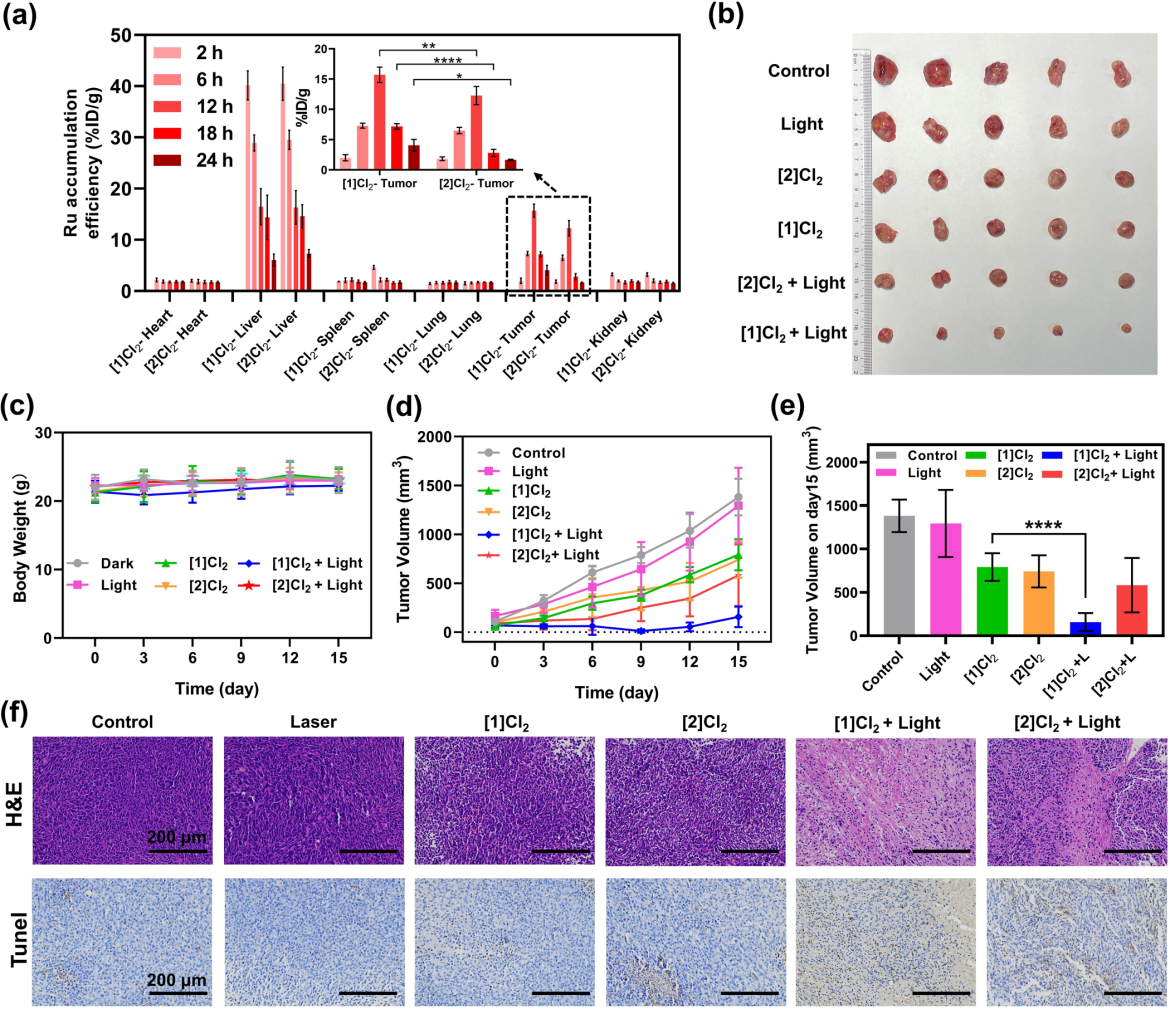
Antitumor effect in vivo. (a) Biodistribution of Ru content (% ID/g, *n* = 3) in major organs of mice at different time points following intravenous injection of [**1**]Cl_2_ (7.7 mg/kg) or [**2**]Cl_2_ (5 mg/kg). % ID/g = Ru content (μg)/tissue (g) compared to total injection of Ru (μg) determined by ICP-OES. Inset: Ru content in the tumor tissue. (b) Photographs of the tumor captured on day 15 for mice treated with phosphate-buffered saline (PBS), light, [**1**]Cl_2_, [**2**]Cl_2_, [**1**]Cl_2_ + light, or [**2**]Cl_2_ + light. Body weight (c) and tumor volume (d) of mice following time evolution after treatments for 15 days, *n* = 5. (e) Tumor volume of mice on day 15 from Figure 6d, *n* = 5. (f) H&E and TUNEL stained images of tumor slices after different treatments on day 7. All of the errors in Figure 6 represent the standard deviation (SD); two-way analysis of variance (ANOVA) was used to determine the significance of the comparisons of data (*P < 0.05; **P < 0.01; ****P < 0.0001).

Considering the outstanding light-activated anticancer effect and integrin-targeting properties of Λ-[**1**]Cl_2_ and Δ-[**1**]Cl_2_*in vitro*, *in vivo* studies were undertaken using a subcutaneous U87MG nude mice tumor model. Since limited differences in activity were observed between the two isomers, nonseparated mixtures containing 40% of Λ-[**1**]Cl_2_ and 60% of Δ-[**1**]Cl_2_, which we call **[1]**Cl_2_ hereafter, were used for this *in vivo* study, as it could be obtained in larger amounts than the isolated isomers. To evaluate the targeting effect of the RGD cyclic peptide, an RGD-free analogue compound **[2]**Cl_2_ was also included in the study (as a racemate). The anticancer effects of these two compounds on the normoxic 2D monolayer U87MG cell *in vitro* are shown in Figure S34a. In accordance with the previous report,^42^ [**2**]Cl_2_ possesses higher cytotoxicity both in the dark and upon light irradiation than [**1**]Cl_2_, leading to a PI of 6, while [**1**]Cl_2_ as a mixture of diastereoisomers had similar activity compared to the individual isomers, with a PI of 16. In order to investigate tumor targeting and to determine the drug-to-light interval (DLI), which is the time point that offers the maximum tumor accumulation of the compound and hence where laser irradiation of the tumor should be performed, a biodistribution study was realized first. Following intravenous injections of the same molar amount of [**1**]Cl_2_ (7.7 mg/kg, Mw = 1493 g/mol) or [**2**]Cl_2_ (5 mg/kg, Mw = 975 g/mol) in the tail of glioblastoma-bearing nude mice, inductively-coupled plasma optical emission spectroscopy (ICP-OES) was used at different time points to quantitatively evaluate the Ru concentration of each resected tumor and organs (Figure 6a and Table S7). The liver was the major organ for microphase Ru uptake, and both compounds presented similar hepatic pharmacokinetics at 24 h post-injection. Maximum accumulation of both compounds in the tumor was found 12 h after injection, suggesting that this time point should be used as DLI. Most importantly, at this time point the amount of ruthenium in the tumor was found to be 28% higher (15.7 ± 1.3%ID/g) for the group treated with the RGD-functionalized prodrug [**1**]Cl_2_ than for the group treated with [**2**]Cl_2_ (12.3 ± 1.5%ID/g, *P* < 0.01). This effect was stronger at *t* = 18 h, where the amount of Ru in the tumor was almost twice as high for [**1**]Cl_2_ (7.2 ± 0.5%ID/g) than for [**2**]Cl_2_ (2.8 ± 0.6%ID/g). Overall, the presence of the tumor-targeting RGD peptide not only achieved excellent delivery of [**1**]Cl_2_ in the tumor 12 h after injection (Table S7) but also efficiently increased the retention time of the drug in tumors. This combination of effects could offer a prolonged DLI window for the activation of the drug in future clinical trials.^67^ It should be noted that next to active targeting by peptide conjugation, passive tumor targeting by the EPR effect may play a role for [**1**]Cl_2_ as well, considering the self-aggregation properties of this amphiphilic compound.^68^ EM images of tumor cells captured at 12 h after injection confirmed the existence of nanoparticles (Figure S35, red arrows), which can be one explanation for the improved retention time of [**1**]Cl_2_ in the tumor area. It should also be acknowledged here that the accumulation of the nontargeted compound [**2**]Cl_2_ was also relatively excellent. We may suggest the possible interaction of [**2**]Cl_2_ with serum albumin, whose role is also to transport hydrophobic compounds in blood circulation. More *in vivo* studies would be needed to confirm this hypothesis.

Considering the promising accumulation of [**1**]Cl_2_ and [**2**]Cl_2_ in the tumor at 12 h, we finally evaluated the antitumor efficacy of both compounds in the subcutaneous glioblastoma-bearing Balb/c mouse model. All mice were randomly divided into 6 groups (*n* = 5) and received *via* tail vein injection various treatments of vehicle control (PBS), vehicle control + light, [**1**]Cl_2_, [**2**]Cl_2_, [**1**]Cl_2_ + light, or [**2**]Cl_2_ + light. As in the biodistribution experiment, the same molar amount was used for both compounds, resulting in a higher mass amount for [**1**]Cl_2_ (7.7 mg/kg) than for [**2**]Cl_2_ (5 mg/kg) in both light and dark groups. After one treatment at day 0 and laser illumination at a light dose of 60 J/cm^2^ using a DLI of 12 h, the tumor volume and body weight of each group was recorded every three days for 15 days, after which the mice were sacrificed for histological analysis of the tumor tissues (Figure S34b). Tumor weights and tumor photos of all groups (15 days) were in accordance with the tumor volume results (Figure 6b,d,e). According to the time evolution of the tumor volumes (Figure 6d), both compounds were found to have similar antitumor properties in the dark. However, in the light groups, both compounds behaved differently. [**1**]Cl_2_ + Light showed a much stronger tumor growth suppression, compared to the dark group only treated with [**1**]Cl_2_. For [**2**]Cl_2_, the effect of light irradiation on the antitumor efficiency of the compound was statistically nonsignificant (Figure 6e). Most importantly, [**1**]Cl_2_ + light had better antitumor efficiency compared to [**2**]Cl_2_ + light. Hematoxylin and eosin (H&E) staining images of tumor slices were taken on day 7. They showed that a majority of cells in the tumor tissues were severely damaged in the [**1**]Cl_2_ + light and [**2**]Cl_2_ + light groups (Figure 6f). TUNEL staining results demonstrated that both [**1**]Cl_2_ and [**2**]Cl_2_ induced outstanding apoptosis of tumor cells when combined with 520 nm laser irradiation (Figure 6f). These *in vivo* results are interesting to compare to cytotoxicity studies *in vitro*, where [**2**]Cl_2_ showed higher cytotoxicity (EC_50,light_ = 0.3 μM) than [**1**]Cl_2_ (EC_50,light_ = 2.4 μM, see Figure S34a) toward U87MG; the most cytotoxic compound *in vitro* is not necessarily the one that shows the highest efficiency *in vivo*. Overall, it seems that conjugation of the targeting peptide to ruthenium brought additional efficiency to the tumor treatment in real physiological conditions. As an important note, 15 days after treatment, all mice experienced negligible weight fluctuation (Figure 6c), and no obvious pathological changes or damages were detected in the major organ tissues from H&E staining images of all groups (Figure S36). Altogether these data suggest that the targeted ([**1**]Cl_2_) light-activated ruthenium prodrugs combined a strong antitumor effect and negligible toxicity to the mice.

## 3 Discussion

Initially, we designed compound [**1**]Cl_2_ as the first tumor-targeting, small-molecule PACT compound reminiscent of cyclic RGD peptides. Even though full photosubstitution of the RGD pentapeptide from [**1**]Cl_2_ was observed in the chemical laboratory, ultimately leading to the formation of the cytotoxic bis-aqua [Ru(Ph_2_phen)_2_(H_2_O)_2_]^2+^ species, quantification of the quantum yields of the two photosubstitution steps showed that the second step is much slower than the first one. In a biological setting, depending on the light dose reaching each individual cell, either the intermediate photoproduct (with a κ^N^-coordinated peptide and a coordinated H_2_O molecule) or the bis-aqua photoproduct (together with the free peptide), will be present and interact with biomolecules, to form secondary photoproducts. These secondary photoproducts are clearly emissive and at the same time capable of generating ROS. As a result, upon treatment with [**1**]Cl_2_ and light, cell death seems to be a consequence of a combination of PACT and PDT effects. Such dual action has been implemented by design by different research groups, for example, by combining polypyridyl ligands promoting ^1^O_2_ generation (e.g., dppn) and photolabile ligand(s) (e.g., MeCN) within the same ruthenium complex.^21,69^ These systems were only tested *in vitro*, but high photoindexes were usually observed, which led to the claim that combining both modes of action was beneficial for photoactivated compounds. Here, we show that the “PACT” compound [**1**]Cl_2_ showed, in fact, unexpected photodynamic effects, and hence that it similarly combines a PDT and PACT mode-of-action. Considering its excellent antitumor effects *in vivo*, this compound suggests that compounds combining PDT and PACT effects are very efficient indeed, but we cannot, at this moment, compare it with a complex working *via* only a PDT or only a PACT mechanism.

Second, though [**1**]Cl_2_ is a chiral compound, it seems that the Λ or Δ configuration of the metal center is not relevant in terms of biological activity. The dissociation constant (*K*_d_) of Λ-[**1**]Cl_2_ with integrin α_IIb_β_3_ (*K*_d_ = 0.061 ± 0.003 μM) was found slightly lower than that of the Δ diastereomer (*K*_d_ = 0.083 ± 0.001 μM), but the cytotoxicity of both diastereoisomers (EC_50_ and photoindex) *in vitro* showed only insignificant differences. Upon irradiation, the chirality of ruthenium polypyridyl compounds is accompanied by racemization from Λ to Δ or *vice versa*. Photoracemization, in fact, has been reported a long time ago for photosubstitutionally nonlabile complexes such as [Ru(bpy)_3_]^2+^ and analogues;^46^ it is usually deemed a relatively quick process. For the photosubstitutionally labile complex [**1**]Cl_2_, the CD signature of either Λ- or Δ-[**1**]Cl_2_ gradually disappeared during light activation, demonstrating that photoracemization in this compound was slower than thioether photosubstitution but faster than histidine photosubstitution. *In vivo*, as the light dose received by different cells varied, it would be difficult to say which isomer is responsible for the observed biological effects. Upon extensive irradiation (i.e., at high light doses) the chirality of the metal center will not be retained, and a racemic mixture of the photoproduct will be obtained. This consideration, combined with the easier purification and higher preparative yields, also explains why we opted for testing [**1**]Cl_2_ as a mixture of Λ and Δ isomers in mice tumor models. Still, as for any chiral (pro)drug, the biosafety of each isomer might have to be tested independently if such compounds would ever follow further clinical developments.

## 4 Conclusions

In this work, we proposed a new strategy for the construction of tumor-targeted ruthenium-based PACT compounds. This design consisted of the direct coordination of an RGD-containing pentapeptide to a cytotoxic ruthenium fragment using histidine and methionine terminal residues. This design produced the two diastereoisomers Λ-[**1**]Cl_2_ and Δ-[**1**]Cl_2_ of a cyclic ruthenium-peptide that could be separated by HPLC and characterized individually. Despite the very hydrophobic ruthenium fragment, these cyclic RGD conjugates were soluble in aqueous solutions, where the peptide remained stably conjugated to the metal, thereby preventing the binding of the ruthenium core to biomolecules. Light activation released the free peptide in two well-identified photochemical steps, together with a cytotoxic ruthenium-containing warhead. In cells, these photosubstitution reactions were accompanied by the generation of significant amounts of ROS, overall resulting in a combination of PACT and photodynamic (PDT) cell-killing mechanisms. Due to their amphiphilic structure, these conjugates self-assembled into nanoaggregates in serum-containing media, thereby making their own drug-delivery system. *In vitro* a correlation between the integrin expression level of the cancer cell line and the cellular uptake of the Ru prodrug was established. In an *in vivo* subcutaneous glioblastoma (U87MG) mice model, the targeted compound [**1**]Cl_2_ showed better retention in the tumor at *t* = 12, 18, and 24 h, respectively, after prodrug injection, compared to its nontargeted analogue [**2**]Cl_2_, though [**2**]Cl_2_ reached the tumor in surprisingly high amounts. Upon light activation with a DLI of 12 h, compound [**1**]Cl_2_ + light showed better antitumor efficiency compared to nonactivated [**1**]Cl_2_, and a significantly better antitumor efficacy compared to [**2**]Cl_2_ + light. Overall, these results propose the coordination of targeting peptides to ruthenium warheads as a promising strategy to obtain tumor-targeted photoactive compounds and demonstrate the interest of [**1**]Cl_2_ as prodrug candidate for the phototherapeutic treatment of glioblastoma. Further research should aim at extending light activation in the red or near-infrared region of the spectrum, which bears a higher potential for human medicine.

## 5 Experimental Section

### 5.1 Photochemistry Studies

The photochemistry of different compounds was studied *via* a combination of methods: by monitoring the evolution of the absorbance (UV–vis) spectra of a solution of the compound irradiated with light, by circular dichroism (CD), and by high-performance liquid chromatography (HPLC) under light irradiation. Mass spectra (MS) were tested before and after light irradiation.

#### 5.1.1 Photosubstitution

##### 5.1.1.1 Following Photosubstitution Using UV–Vis Spectroscopy and Photosubstitution Quantum Yield Calculation

UV–vis spectroscopy was performed using a Cary 60 spectrometer from Varian equipped with a temperature control set to 25 °C and a magnetic stirrer. Experiments were performed in a 1 cm quartz cuvette containing 3 mL of solution. The desired complex was prepared using MilliQ water or acetonitrile to a certain concentration. A beam of green light produced by a cooled 515 nm LED (photon flux = 1.77 × 10^–8^ photons cm^–2^ s^–1^) was shone perpendicularly from the top of the cuvette, and the light was turned on immediately when recording started. Standard measurement method: a spectrum measurement (from 800 to 200 nm) was performed every 30 s until 120 min. Photosubstitution quantum yield calculations were analyzed with Microsoft Excel and Glotaran, as explained in detail by Bahreman and Bonnet.^70^

##### 5.1.1.2 Following Photosubstitution by Circular Dichroism

Circular dichroism spectra were collected from 200 to 600 nm with the step of 1 nm on a Bio-Logic MOS-500 spectrometer at 25 °C. A 0.2 mm path-length quartz cuvette was used for a specific concentration of a complex solution. Samples dissolved in water (67 μM) were measured sequentially by CD after irradiation with 515 nm LED green light (the same as UV–vis) for 0, 2, 7, 20, 60, 120, and 150 min. Every spectra were measured at least 3 times, and the spectra were averaged and smoothed after background subtraction.

##### 5.1.1.3 Following Photosubstitution by High-Performance Liquid Chromatography (HPLC)

The analysis of compound dissociation was performed using a 250 mm × 21.2 mm Jupiter 4 μm Proteo 90 Å C_12_ column using a Thermo Scientific UHPLC system equipped with four UV detectors (214, 290, 350, 450 nm). The gradient was controlled by four pumps with a total flow rate = 14 mL/min. The mobile phase consisted of H_2_O containing 0.1% v/v formic acid (phase A) and acetonitrile containing 0.1% v/v formic acid (phase B). A standard method of two-phase followed the gradient: 10–90% phase B/phase A, 25 min, flow rate = 14 mL/min, UV channel = 290 nm. Samples dissolved in water (0.67 mM) were injected sequentially after irradiation with 515 nm LED green light (the same as UV–vis) for 0, 2, 4, and 7 min.

#### 5.1.2 Determination of ^1^O_2_ Generation Quantum Yields

Singlet oxygen quantum yield measurements were performed by direct spectroscopic detection of the 1275 nm emission, as described by Meijer et al.^71^

### 5.2 Nanoaggregation

#### 5.2.1 Size Distribution According to Dynamic Light Scattering (DLS)

DLS was used to determine the distribution of particles in complex solutions (50 μM) in Opti-MEM (Gibco complete medium 11058-021, supplemented with 0.2% v/v penicillin/streptomycin (P/S), and 1% v/v glutamine) with or without 2.5% fetal calf serum (FCS) proteins *via* a ZEN1600 Zetasizer Nano instrument (Malvern Instruments Limited) operated with a 633 nm laser.

#### 5.2.2 TEM Measurement of Metal Complexes in Different Solutions

The TEM experiments were carried out with a TEM JEOL 1010:100 kV transmission electron microscope using a Formvar/carbon-coated copper grid (Polysciences Inc.). For the preparation of samples, a 1 mM stock aqua solution was diluted to 50 μM by Opti-MEM, after which, each drop (15 μL) of the complex solution was deposited on a parafilm (Bemis, HS234526C). The grids (Electron Microscopy Sciences, 71137) were placed on top of the drops for 5 min and then the excess liquid on the grid was removed with filter paper and air dried for 2 h for the TEM measurement. The TEM measurements were carried out under vacuum conditions.

### 5.3 Cytotoxicity Assay

#### 5.3.1 2D Cytotoxicity Assay with the 24 h DLI & No Wash Protocol

A549 cells (5000), U87MG cells (6000), and PC-3 cells (6000) were seeded in 96-well plates (Sarstedt, 83.3924) at *t* = 0 h; each well contains 100 μL of Opti-MEM (Gibco complete medium 11058-021, supplemented with 2.5% v/v fetal calf serum (FCS), 0.2% v/v penicillin/streptomycin (P/S), and 1% v/v glutamine). 24 h later, six different concentrations of Λ-[**1**]Cl_2_, Δ-[**1**]Cl_2_, or cisplatin (0.5 to 50 μM) dissolved in Opti-MEM were added to the wells in triplicate, reaching a total medium volume of 200 μL in each well. For each complex, two identical plates were prepared, one of which was irradiated with light, while the other was used as a dark control. Plates were incubated in the dark, at 37 °C, normoxia (21% O_2_ and 7.0% CO_2_) or hypoxia (1.0% O_2_ and 7.0% CO_2_) for 24 h. At *t* = 48 h and without removing the excess drug, for each cell type, one plate was irradiated with green light (520 nm) for 20 min at 37 °C for normoxia (dose = 13.1 J/cm^2^, intensity = 10.9 mW/cm^2^) or 30 min for hypoxia (dose = 13.1 J/cm^2^, intensity = 7.22 mW/cm^2^), while the other plate was kept in the dark as the control. The cells were further incubated for another 2 days in the normoxic or hypoxic dark incubator, respectively. Finally, at *t* = 96 h, 100 μL of cold trichloroacetic acid (10% w/v) was added to each well to fix the cells, and all plates were then transferred to a 4 °C refrigerator for 48 h before performing an SRB cell quantification endpoint assay.^72^

#### 5.3.2 2D Cytotoxicity Assay with the 6 h DLI & Wash Protocol

U87MG cells (6000) and PC-3 cells (6000) were seeded in 96-well plates (Sarstedt, 83.3924), each well containing 100 μL Opti-MEM. 24 h later, six different concentrations of Δ-[**1**]Cl_2_, or Δ-[**3**]Cl_2_ (0.5 to 50 μM) dissolved in Opti-MEM were added to the wells in triplicate. For one group of complexes, there are two plates with the same condition except for dark and light. Plates were then incubated in the dark, 37 °C, normoxia (21% O_2_) for 6 h. After 6 h dark incubation, the compound-containing medium was removed and each well was washed with PBS buffer (2 × 150 μL) and refilled with 200 μL of Opti-MEM. At *t* = 30 h, one plate was irradiated with green light (520 nm) for 20 min at 37 °C in normoxia (dose = 13.1 J/cm^2^), while the other plate was kept in the dark as the control. The cells were incubated until *t* = 96 h in normoxia. Finally, at *t* = 96 h, 100 μL of cold trichloroacetic acid (10% w/v) was added to each well, and plates were then transferred to a 4 °C refrigerator for 48 h.

#### 5.3.3 SRB Assay

Trichloroacetic acid was first removed and each plate was gently washed with demi water 3–5 times. Then, each well was dried in the air, and 100 μL of 0.6% SRB solution (0.6% w/v in 1% v/v acetic acid/H_2_O solution) was added into each well where it was allowed to stain for 30 min. Then, the plates were washed 3–5 times using an acetic acid solution (1% v/v). Once the plate was washed it was allowed to dry overnight. Then, 200 μL of 10 mM Tris base buffer was added to each well and the plate was allowed to sit on an orbital shaker for 0.5–16 h. After mixing, the absorbance of each well was determined by an M1000 Tecan Reader, reading at 510 nm. All experiments were conducted in independent biological triplicate. The obtained data were analyzed with Graphpad Prism 5 using the dose–response two-parameter Hill slope equation (eq 1) to obtain the half-maximal effective concentrations EC_50_ (defined as the concentration of drug that kills 50% of cells, compared to the untreated control).
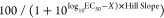
1

#### 5.3.4 3D Tumor Spheroid Viability Assay

U87MG cells (500 cells) were added to a 96-well round-bottomed Corning spheroid (Catalogue CLS4520) microplate and incubated under normoxia for 3 days to generate 3D tumor spheroids (∼500 nm). 100 μL of Opti-MEM was contained in each well. 1 dark and 1 light plate were included in one group. After that, 100 μL of different concentrations of Λ-[**1**]Cl_2_, Δ-[**1**]Cl_2_, or cisplatin dissolved in Opti-MEM were added to the wells in triplicate to reach final concentrations in the wells of 0, 1, 5, 10, 20, 40, and 60 μM. The spheroids were incubated further under normoxia. After 24 h, the light plate was irradiated with a green light for 30 min (dose = 13.0 J/cm^2^), and the other plate was left in the dark. The cells were further incubated under normoxia in the dark for 2 days, and a CellTiter-Glo 3D solution (100 μL/well) was added to each well to stain the 3D tumor spheroids afterward. After 30 min of shaking on an IKA Vibrax shaker at 500 rpm at room temperature, the luminescence in each well was measured with a Tecan microplate reader. Similar to 2D cell culture, half-maximal effective concentrations (EC_50_) for 3D tumor spheroid growth inhibition were calculated by Graphpad Prism 5 using the dose–response two-parameter Hill slope equation (eq 1). All experiments were conducted in three biologically independent replicates.

### 5.4 Measurement of Intracellular ROS

The generation of ROS (reactive oxygen species) in U87MG cells was measured using a ROS deep red fluorescence indicator (Abcam, ab186029). U87MG (1 × 10^5^, 1 mL) were seeded into 12-well plates and incubated for 24 h in the dark under normoxia. The cells were then treated with 15 μM complexes with Opti-MEM, Λ-[**1**]Cl_2_, Δ-[**1**]Cl_2_, cisplatin, or Rose Bengal. There are two groups for each drug (dark + light). After 24 h of incubation under normoxia, the plate was washed with cold PBS once, and cells were trypsinized, harvested, and then resuspended in 150 μL of PBS. The cell suspension from the centrifuge tubes was transferred to 96-round-bottom well plates (Thermo Scientific, 268200), and the light group was irradiated for 20 min with 520 nm light (dose = 13.1 J/cm^2^). After which, the Cellular ROS Deep Red dye (abcam, ab186029) was added with 1000× dilution, and cells were further stained for 1 h. Untreated cells were maintained as negative controls, whereas a 250 μM tBHP solution in Opti-MEM complete was administered as a positive control for ROS. The levels of intracellular ROS were then determined using the CytoFLEX flow cytometer. Forward versus side scatter (FSC vs SSC) gating was used to select the population of interest and avoid cell debris. A forward scatter height (FSC-H) vs forward scatter area (FSC-A) gating was used for doublet exclusion. Fluorescence measurements were acquired with the APC-A (638 nm excitation, 660/10 nm emission) channel given the known excitation/emission wavelengths of the ROS Deep Red dye (650/675 nm, respectively). All flow cytometry data were processed using FlowLogic 8.5 software.

### 5.5 Detection of Secondary Photoproducts by Emission Spectroscopy (FACS) Upon Light Activation in U87MG

1 × 10^5^ cells were seeded in 12-well plates in Opti-MEM (1 mL) and cultured in a normoxic incubator for 24 h. Δ-[**1**]Cl_2_ (10 μM) was added to each well and cells were incubated with the drug for 6 h. The cells were then refreshed with drug-free Opti-MEM medium, divided into 7 groups, and irradiated with a green light (520 nm, 13.1 J/cm^2^) for 0, 5, 10, and 20 min, or further incubated in the normoxic incubator for 2, 12, or 24 h after 20 min irradiation. In each group, cells were collected as follows: cells were typsinized, harvested, washed with cold PBS twice, and then concentrated in 100 μL of PBS. Cells were then transferred to a 96-well round-bottom plate (Thermo Scientific, 268200). The mean fluorescence intensity from the ruthenium-based photoproducts in each cell was then determined using the CytoFLEX flow cytometer using the PC5.5 channel (488 nm excitation, 650/50 nm emission). All flow cytometry data were processed using FlowLogic 8.5 software. Every group was conducted in triplicate, showing the standard derivation as errors. Dark and control cells (treated with a drug-free medium) were added as a comparison in certain groups.

### 5.6 Apoptosis Study

The apoptosis study of U87MG cells induced by Ru-peptide conjugates was measured by an Apopxin/Nuclear Green DCS1 double-staining assay (Abcam, ab176750). 1 mL of aliquots of the U87MG cell suspension (2 × 10^5^ cells/well) were seeded in two 12-well plates (Sarstedt) using Opti-MEM complete medium and allowed to incubate for 24 h in the dark at normoxia, after which 20 μM drug solutions were added. After 24 h of incubation, one plate labeled light was irradiated for 20 min using 520 nm green light (13.1 J/cm^2^), and then both plates were allowed to incubate further for 1, 2, 6, or 24 h in a normoxia incubator. After incubation, the cells were trypsinized, collected, and washed with cold PBS twice. The pellets were resuspended in 200 μL of assay buffer and then stained with 2 μL of the Apopxin Deep Red Indicator (100×) and 1 μL of Nuclear Green (200×) for 30–60 min at room temperature in the dark and then 300 μL of assay buffer was added to increase volume. Cells after staining were detected by flow cytometry (CytoFLEX flow cytometer) immediately. Control groups with only assay buffer, only buffer and Apopxin Deep Red Indicator, only buffer and Nuclear Green DCS1, and all three were included to be used for gating during data analysis. Parameters APC (638 nm excitation, 660/10 nm emission) and FITC (488 nm excitation, 525/40 nm emission) were used considering their similar excitation/emission wavelength with two detectors Apopxin Deep Red Indicator, *E*_x_/*E*_m_ = 630/660 nm (apoptosis), and Nuclear Green DCS1, *E*_x_/*E*_m_ = 490/520 nm (necrosis). All flow cytometry data were processed using FlowLogic 8.5 software.

### 5.7 Integrin Expression Analysis by Flow Cytometry

The double immune-fluorescence method was applied to study the expression of integrin α_V_β_3_ and α_V_β_5_ on the surface of U87MG, U87MG-kd, PC-3, and MCF7 cultured in normoxia (21% O_2_) and hypoxia (1% O_2_) conditions. Cells were cultured in a 25 cm^2^ flask in both conditions for more than one month and then collected and washed with phosphate-buffered saline (PBS) containing 0.1% bovine serum albumin (PBS/BSA). 6 × 10^4^ cells were resuspended in 50 μL of 10 μg/mL (1:100 dilution of stock by PBS) monoclonal antibodies against human α_V_β_3_ (clone LM609, Merck) or human α_V_β_3_ (ab177004, Abcam) for 40 min at 4 °C; after washing with PBS/BSA, cells were incubated for an additional 40 min at 4 °C with 50 μL of 5 μg/mL Alexa-Fluor 488-conjugated goat anti-mouse IgG antibody (Invitrogen, A-11001). After washing with PBS, cells were resuspended in 100 μL of PBS and analyzed by a CytoFLEX flow cytometer using a FITC channel (488 nm excitation, 525/40 nm emission); the data was further proceeded by FlowJo10 software.

### 5.8 Uptake Study by ICP-MS

U87MG cells (6000), PC-3 cells (5000), and MCF7 cells (8000) were seeded in 96-well plates, each well containing 100 μL of Opti-MEM. After 24 h, 12.5 μM drugs dissolved in Opti-MEM were added to each well and the plates were incubated in a normoxic or hypoxic incubator for 6 h. After that, the drug-containing medium was removed from each well, which was washed with PBS buffer once. Then, cells were stained by Nuclear Blue (Invitrogen, R37605) for 30 min to stain all nuclei (2 drops/mL medium). After this, the dye was thereafter removed and replaced with fresh medium. The entire well was imaged with 10× objective magnification and 7 × 6 montage using epifluorescence on a Nikon TiE2000 widefield microscope with a perfect focus system and automated xy-stage. The cell number of every well was analyzed by counting the nuclei using Image-Pro Analyzer 7.0. Thereafter, the medium was removed and the cells were digested by adding 100 μL of 65% HNO_3_ for 30 min at room temperature. The cell lysates were transferred into a 96-deep well plate (Eppendorf, E951033502), followed by the addition of 0.9 mL of MilliQ water into each well, and the plate was then mixed well with a 1000 μL Eppendorf pipette.

The uptake studies in U87MG and U87MG-kd cell lines were conducted as follows: 2 × 10^5^ cells were cultured in 6-well plates for 24 h in Opti-MEM (2 mL), after which cells were incubated with Δ-[**1**]Cl_2_ or Δ-[**3**]Cl_2_ (10 μM) for 2 h, washed with cold PBS twice, harvested, concentrated in 200 μL of PBS, and counted using trypan blue and a cell counter (Bio-Rad). Finally, cell pellets were collected in 1.5 mL Eppendorf tubes after removing additional PBS by centrifuging at 500*g*; 0.25 mL of 65% HNO_3_ was then added to each tube, and all tubes were kept at room temperature (RT) overnight. The cell lysates were transferred to 15 mL centrifuge tubes (Biologix) and filled with 4.75 mL of MilliQ H_2_O.

The ruthenium concentration was measured in each sample by ICP-MS (NexION 2000, PerkinElmer), providing the metal content in each well in ppb. Combining the cell numbers and Ru uptake, averaging over technical triplicates, the ruthenium uptake values were finally expressed in μg Ru/million cells.

### 5.9 Protein Interaction Study by Fluorescence Spectroscopy

Purified human platelet glycoprotein integrin α_IIb_β_3_ was purchased from Enzyme Research Laboratories. Glycerol was removed by the following procedure according to the literature:^33^ The protein (0.7 mL) was moved from −20 °C to 4 °C and then defrozen; 1.4 mL of Tris buffer (20 mM Tris-HCl, 150 mM NaCl, 1 mM CaCl_2_) was added and the protein solution was moved to an Amico ultracentrifugal filter unit (MWCO 5 KDa, washed with MilliQ water and buffer in advance). The diluted protein solution was centrifuged at 4000 rpm at 4 °C for 1 h. After which 1.4 mL of fresh buffer was added again and the centrifugation process was repeated at least 3 times. The resulting protein solution was around 800 μL, and the concentration was determined to be 4.2 μM by absorption spectroscopy (*A*_protein_ = 280 nm, extinction coefficient (1%) = 9.1). The solution was divided into 6 tubes and stored at −20 °C until further required.

The protein working solution was prepared by adding a certain volume of Tris buffer (20 mM Tris-HCl, 150 mM NaCl, 1 mM CaCl_2_) to the stock solution (4.2 μM) after it had been taken out from the freezer. The protein-drug mixture was prepared by adding a different volume of drug stock solution (25 μM) to 500 μL of protein working solution (0.1 μM) to make the drug working concentration as indicated in Table S5. The emission spectrum of the solution was measured every time by an F900 Florescence spectrometer (Edinburgh Instruments) with an excitation wavelength of 280 nm. The dissociation constant of the drug to integrin α_IIb_β_3_ was determined by the Stern–Volmer eq 2
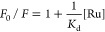
2where *F*_0_ and *F* are the fluorescence intensities at 345 nm (arbitrary units) in the absence and presence of the complex, respectively, [Ru] is the concentration of the complex after correction (in M), and *K*_d_ is the dissociation constant (in M), which was obtained from the reciprocal of the slope of the Stern–Volmer plot *F*_0_/*F* vs [Ru].

### 5.10 Log *P* Measurements

Each complex was first dissolved in octanol-saturated water (0.5 mM, 500 μL) and centrifuged (2000 rpm, 5 min) to isolate the undissolved solid. 450 μL of the supernatant was taken out as the stock solution; after that, 5, 10, 50, 100, and 150 μL of solution were added to the 15 mL tube, and more octanol-saturated water was added to adjust the final volume to 1 mL. 1 mL of water-saturated octanol was added further, and the tubes were mildly shaken (Gesellschaft für Labortechnik mbH, type3016) for 24 h under dark conditions. After that, the solutions were centrifuged for 5 min at 2000 rpm at room temperature, and 0.5 mL of the water phase (below phase) was moved to a 15 mL centrifuge tube by an Eppendorf pipette. Then, 0.1 mL of 65% HNO_3_ was added into the tube using an organic pipette gun, and each solution was shaken for 1 h. 9.9 mL of MilliQ water was added into each tube, making a total volume of 10 mL. To detect the Ru content of the stock solution, 10 μL of each stock solution was mixed with 0.490 mL of 65% HNO_3_, and the mixture was shaken for 1 h. Then, 9 mL of MilliQ water was added to the tube, making a total volume of 10 mL. ICP-MS measurement was performed using a NexION 2000 from PerkinElmer. The ruthenium content in each well was obtained in ppb. The partition coefficients (log *P*) were calculated using the following eq 3
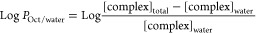
3

where [complex]_total_ is the concentration of the complex in the control sample (where no water-saturated octanol was added) and [complex]_water_ is the concentration of the complex in the aqueous layer.

### 5.11 *In Vivo* Antitumor Study

#### 5.11.1 Subcutaneous Solid Tumor Model Construction

All animal studies were approved by the Institutional Animal Care and Use Committee of the US National Institutes of Health and were performed according to the relevant guidelines (8th edition, 2011). Female BALB/c nude mice (6 weeks old) were purchased from Shanghai SLAC Laboratory Animal Co. LTD (Shanghai, China). Tumor-bearing mice were obtained by subcutaneously injecting U87MG tumor cells (2 × 10^6^ cells per mouse, dispersed in 100 μL of FBS) into the right hind limb and waiting for 15 days until the tumor volume reached 50–100 mm^3^.

#### 5.11.2 Biodistribution Evaluation

The Blab/c nude mice bearing U87MG tumors were randomly assigned to two groups (*N* = 15) and received an intravenous injection of [**1**]Cl_2_ (7.7 mg/kg) or [**2**]Cl_2_ (5 mg/kg) at the same molar dose. Then, the mice were sacrificed at 2, 6, 12, 18, and 24 h post-injection (each time point contained 3 mice). The main organs (heart, liver, spleen, kidney, lung) and tumor tissue were dissected. Then, around 1 g of organs and tumors was lysed in a 7 mL mixture solution containing 5 mL of 65% HNO_3_ and 2 mL of 30% (w/w) H_2_O_2_ at 100 °C. After 12 h, all solutions had evaporated, and 5 mL of aqueous solution containing 2% HNO_3_ was added. The Ru content in all samples was measured by ICP-OES (JY-Horiba ICP-OES Ultima 2).

#### 5.11.3 *In Vivo* Tumor Inhibition Studies

The mice bearing U87MG solid tumors were first randomly divided into 6 groups (*N* = 5): PBS, light, [**1**]Cl_2_, [**2**]Cl_2_, [**1**]Cl_2_ + light, and [**2**]Cl_2_ + light. The injectable solution of [**1**]Cl_2_ or [**2**]Cl_2_ was obtained by diluting the stock solution of the compound in DMSO (10 mg/mL) 10 times by RPMI-1640 medium containing 10% FBS, 100 units/mL penicillin, and 100 μg/mL streptomycin. The injection doses of [**1**]Cl_2_ and [**2**]Cl_2_ were set as 7.7, 5 mg/kg, respectively (injection volume = 100 μL of the same molar weight of Ru in the last four groups). In groups 2, 5, and 6, laser irradiation (520 nm, 100 mW/cm^2^, 5 min) was carried out twice at 12 h post-injection, with a 5 min interval. Accordingly, the total laser dose for each illumination was 100 mW/cm^2^, 10 min, and 60 J/cm^2^. Treatments in all groups were carried out only once on day 0. Digital photos of tumor-bearing mice, the tumor size (length and width, measured with a caliper), and mouse body weight in all groups were recorded every third day. The tumor volume was calculated according to the standard formula: 0.5 × length × width^2^. Both the average tumor volume and body weight were followed for 15 days. On day 7, one mouse from each group was sacrificed and then the tumor tissue was dissected and fixed with 10% paraformaldehyde. The tumor cell damage and apoptosis and necrosis conditions were evaluated by H&E or TUNEL stained protocols. On day 15 (the end of the tumor treatment period), all nude mice were humanly sacrificed, and the main organs (heart, liver, spleen, kidney, and lung) and tumors were resected. Digital photographs of tumors in each group were immediately obtained. All normal tissues were fixed with 10% paraformaldehyde and further analyzed in accordance with the H&E staining protocol, to estimate their off-target side effect after various treatments.

#### 5.11.4 TEM Photographs of Tumor Cells *In Vivo*

Two groups of tumor-bearing mice (*N* = 4) were treated with [**1**]Cl_2_ (7.7 mg/kg) (100 μL of RMPI 1640 medium) and PBS 1× (100 μL) through intravenous tail injection, respectively. After 12 h post-injection, all mice were sacrificed and then the tumor tissues were removed and immersed with a biological TEM fixation buffer (Wuhan Servicebio). Subsequently, the tumor tissues were cut into small fragments with a size of ∼1 mm^3^, and refixed by incubation with 1% osmic acid PB buffer for more than 2 h. Next, all of the above samples were dehydrated by ethanol under various concentrations (v/v = 30, 50, 70, 80, 95, or 100%, 20 min incubation) and pure acetone treatment (15 min) for two times. Latterly, all of the above samples were embedded with acetone/epon-812 medium with the volume ratio 1:1 for 2 h and 1:2 for 12 h, respectively. They were further treated with pure epon-812 at 37 °C for another 5 h incubation. The tumor tissue-containing embedding buffers were immersed in the embedding mold at 37 °C for 24 h, followed by 60 °C incubation for 48 h. Then, all obtained tissue-containing resins were cut into slices (∼60–80 nm thick around) by an ultramicrotome (Leica EM UC7), and the samples were immediately coated on the copper grid (300 mesh). The prepared grids were stained by uranyl acetate/ethanol (v/v = 2%) solution for 8 min and lead citrate/water (v/v = 2.6%) solution for another 8 min. Finally, after drying at room temperature overnight, the grids were observed using a JEOL JEM2100 TEM (Japan).

## Supporting Information Available

Materials, synthesis, and characterization data (1D and 2D NMR, HPLC, HR-MS), photochemistry data including photosubstitution and singlet oxygen generation quantum yields, protein interaction studies (*K*_d_), log *P* determination, phototoxicity data with and without washing, cellular uptake data, integrin expression data, cell death mechanism studies in cancer cell monolayers (ROS generation), and in vivo antitumor experiments (PDF)
