# Supplementary material for: Cyclic Ruthenium-Peptide Conjugates as Integrin-Targeting Phototherapeutic Prodrugs for the Treatment of Brain Tumors
Source: J Am Chem Soc. 2023 Jun 28;145(27):14963–80. doi: 10.1021/jacs.3c04855 (PMC10347550; doi:10.1021/jacs.3c04855)
Supplement: Supplementary file 1 — ja3c04855_si_001.pdf [file ja3c04855_si_001.pdf]

Supporting Information

of

Cyclic ruthenium-peptide conjugates as integrin-targeted phototherapeutic prodrugs for the treatment of brain tumors

Liyan Zhang,[a] Peiyuan Wang,[b,c] Xue-Quan Zhou,[a,b] Ludovic Bretin,[a] Xiaolong Zeng,[b] Yurii Husiev,[a] Ehider A. Polanco,[a] Gangyin Zhao,[d] Lukas S. Wijaya,[e] Tarita Biver,[f] Sylvia E. Le Dévédec,[e] Wen Sun,[b]* Sylvestre Bonnet[a]*

[a] Leiden Institute of Chemistry, Universiteit Leiden, Einsteinweg 55, 2333 CC, Leiden, Netherlands.

[b] State Key Laboratory of Fine Chemicals, Dalian University of Technology, 2 Linggong Road, Dalian 116024, P. R. China.

[c] Key Laboratory of Design and Assembly of Functional Nanostructures, Fujian Institute of Research on the Structure of Matter, Chinese Academy of Sciences, Fuzhou 350002, P. R. China.

[d.] Leiden Institute of Biology, Universiteit Leiden, Einsteinweg 55, 2333 CC, Leiden, Netherlands.

[e] Leiden Academic Centre for Drug Research, Universiteit Leiden, Einsteinweg 55, 2333 CC, Leiden, Netherlands.

[f] University of Pisa, Department of Chemistry and Industrial Chemistry, 56124 Pisa, Italy.

Corresponding author email: bonnet@chem.leidenuniv.nl; sunwen@dlut.edu.cn

Table of Contents

1 General information 3

2 Synthesis and characterization 3

3 Photochemistry 13

4 Nanoaggregate characterization 17

5 Cytotoxicity study without washing: dose-response curves 18

6 $^1O_2$ generation and intracellular ROS generation 20

7 Detection of secondary photoproducts by FACS in U87MG treated with Δ- [1]Cl_2 and light 22

8 Apoptosis assay by FACS 23

9 Integrin $\alpha_v\beta_3$ and $\alpha_v\beta_5$ expression by FACS analysis 24

10 Cellular uptake study by ICP-MS 25

11 Protein interaction study 26

12 Lipophilicity (log P) study 27

13 Cytotoxicity study including a washing step before irradiation: dose-response curves 27

14 *In vivo* antitumor study 28

1 General information

All reagents were purchased from commercial suppliers. The reactants and solvents were used without further purification. The peptides were synthesized by a CEM Liberty automated microwave peptide synthesizer. Electrospray ionization mass spectra were recorded by using a MSQ Plus Spectrometer in the positive ionization mode. ^{1}H NMR were obtained on a Bruker DMX-400 spectrometers, chemical shifts are indicated in ppm relative to the residual solvent peak. HPLC was accomplished by Thermo Scientific Dionex Ultimate 300 system equipped with a 250 x 21.2 mm Jupiter® 4 µm Proteo 90 Å C_{12} column. UV-vis spectra were recorded on a Cary 60 spectrometer from Varian. The emission spectra were measured *via* a F900 Spectrometer from Edinburgh Instruments Ltd. Absorbance measurements for analysis of 96-well plates were done by a M1000 Tecan Plate Reader. The TEM experiments were carried *via* TEM JEOL 1010: 100 kV transmission electron microscope using Formvar/Carbon coated copper grid from Polysciences Inc. Flow cytometry were conducted either by BD FACSCanto™ II Clinical Flow Cytometer or CytoFLEX flow cytometer. Human cancer cell lines A549 (adenocarcinoma alveolar basal epithelial cells), U87MG (primary glioblastoma cells), PC3WT (prostate cancer cells) and MCF7 (breast cancer cell) were distributed by the European Collection of Cell Cultures (ECACC) and purchased from Sigma Aldrich. Dulbecco's Modified Eagle Medium (DMEM, D6546), Glutamine-S (GM; 200 mm), penicillin/streptomycin (P/S), tris(hydroxylmethyl)aminomethane (Tris base), trichloroacetic acid (TCA), glacial acetic acid, and sulforhodamine B (SRB) were purchased from Sigma-Aldrich. Opti-MEM Reduced Serum Media without phenol red was obtained from Gibco (11058-021). Rose Bengal and $Ru(bpy)_3Cl_2$ were ordered from Sigma-Aldrich. Nuclear Blue (catalog No. R37605) was purchased from Invitrogen by Thermo Fisher Scientific. Cellular ROS Assay Kit (Deep Red, tBHP (tert-Butyl hydroperoxide) included) was purchased from Abcam (ab186029). Anti-integrin $\alpha_V\beta_3$ antibody for integrin expression study was purchased from Merck (MAB1976), Anti-Integrin $\alpha_V\beta_5$ antibody [P1F6] was from Abcam (ab177004). The Alexa Fluor™ 488 conjugated goat anti-mouse IgG (H+L) cross-adsorbed secondary antibody, was ordered from Invitrogen by Thermo Fisher Scientific (A-11001). Apoptosis/Necrosis Detection Kit(blue, red, green) was ordered from Abcam (ab176750). Purified human platelet glycoprotein integrin $\alpha_{IIb}\beta_3$ was purchased from Enzyme Research Laboratories (GP2b3a). Peptide Ac-MRADH-NH_2 was ordered from Biomatik.

2 Synthesis and characterization

4,7-Diphenyl-1,10-phenanthroline and $RuCl_3{\cdot}3H_2O$ were purchased from Sigma-Aldrich. The amino acids Fmoc-Met-OH, Fmoc-Arg(Pbf)-OH, Fmoc-Gly-OH, and Fmoc-Asp(OtBu)-OH as well as Fmoc-His(Trt)-OH for peptide synthesis were purchased from MerckMillipore. All reactants and solvents were used without further purification. The synthesis of cis-$Ru(Ph_2phen)_2Cl_2$ and rac-$[Ru(Ph_2phen)_2(mtmp)]Cl_2$ ([**2**]Cl_2) were carried out using literature procedures.[1]

Ac-MRGDH-NH_2 and Ac-MRADH-NH_2

The peptides Ac-MRGDH-NH_2 were synthesized on a CEM Liberty microwave synthesizer, according to standard Fmoc solid-phase protocols. The peptides were synthesized from their C-termini to N-termini. The acetylation of N-termini was realized by 1:1:3 acetic anhydride/pyridine/DMF reacting for 1 h. After cleavage from the resin (95% TFA : 2.5% H_2O : 2.5% Triisopropylsilane), the collected peptides were precipitated with cold diethyl ether, stored at 4 °C for 3 h and then washed 3 times by cold diethyl ether. The peptides were then dissolved in MilliQ water and freeze-dried for further LC-MS test. The peptide's purity was characterized to be >90% (Figure S1), it was used directly in the next step without any further purification. Ac-MRADH-NH_2 was ordered from Biomatik with 95% purity.

[Ru(Ph_2phen)$_2$(Ac-MRGDH-NH_2)]Cl_2 ([1]Cl_2)

Ru(Ph_2phen)$_2Cl_2$ (0.025 mmol, 20.9 mg) was added to a 2-neck 25 mL round-bottom flask, the peptide powder Ac-MRGDH-NH_2 (0.025 mmol, 16.40 mg) was dissolved in demi-water (2.5 mL) and adjusted to pH = 7.5 using 0.5 mM NaOH and 0.1 mM and HCl solutions. After adding ethanol (2.5 mL), the peptide solution was degassed by N_2 bubbling for 10 min to remove the O_2. After the flask had been put under vacuum and re-filled with N_2 thrice, the deoxygenized peptide solution was then injected to the reaction flask under N_2. The mixture was then heated at 60 °C for 3 days. After that, the reaction solution was cooled down to room temperature, ethanol was firstly removed by rotary evaporation. After filtration and washing of the solid by cold water, the filtrate was freeze-dried, to afford a reddish powder. Further purification was accomplished by HPLC. The purification was performed by a 250 × 21.2 mm Jupiter® 4 µm Proteo 90 Å C12 column using Thermo Scientific UHPLC system. The gradient was controlled by four pumps. The mobile phase consisted in H_2O containing 0.1% v/v formic acid (phase A) and acetonitrile containing 0.1% v/v formic acid (phase B). The gradient for the preparative separation of [**1**]Cl_2 was 30-40% phase B/phase A for 20 min. The fractions were monitored by four UV detector (set at 214 nm, 290 nm, 350 nm, and 450 nm) and the flow rate was 14 mL/min. The compound was collected at UV-detector 290 nm. After lyophilization, two enantiotimers were obtained as orange-red powder (the yield for Λ-[**1**]Cl_2 and Δ-[**1**]Cl_2 was around 11% and 17%, respectively). Λ-[1]Cl_2 **HR-MS** *found* (*calc*): *m/z* = 710.7267 (710.7260 for Λ-[**1**]$^{2+}$, [$C_{73}H_{73}N_{15}O_8RuS$]$^{2+}$) and 474.1542 (474.1537 for [Λ-[**1**]+H]$^{3+}$, [$C_{73}H_{74}N_{15}O_8RuS$]$^{3+}$). **HPLC** (10-90% phase B/phase A, 25 min): t_R = 12.8 min; **^{1}H NMR** (400 MHz, Methanol-d_4) δ 10.14 (s, 1H), 9.40 (d, J = 5.4 Hz, 1H), 8.38 (d, J = 9.5 Hz, 1H), 8.30 – 8.21 (m, 3H), 8.18 – 8.10 (m, 2H), 8.03 (d, J = 5.5 Hz, 1H), 7.87 (d, J = 7.3 Hz, 2H), 7.80 (d, J = 7.0 Hz, 4H), 7.70 (m, 8H), 7.60 (d, J = 5.5 Hz, 1H), 7.59 – 7.53 (m, 14H), 7.00 (s, 1H), 4.64 (s, 2H), 4.53 (s, 1H), 4.47 (s, 1H), 4.23 (s, 1H), 4.05 (d, J = 16.8 Hz, 1H), 3.80 – 3.64 (m, 1H), 3.51 – 3.46 (m, 1H), 3.27 – 3.11 (m, 1H), 2.85 (d, J = 12.7 Hz, 1H), 2.72 (s, 2H), 2.16 (s, 2H), 1.92 (s, 3H), 1.67 (s, 6H).

Δ-[**1**]Cl_2 **HR-MS** *found* (*calc*): *m/z* = 710.7267 (710.7260 for Λ-[**1**]$^{2+}$, [$C_{73}H_{73}N_{15}O_8RuS$]$^{2+}$) and 474.1542 (474.1537 for [Λ-[**1**]+H]$^{3+}$, [$C_{73}H_{74}N_{15}O_8RuS$]$^{3+}$). **HPLC** (10-90% phase B/phase A, 25 min): t_R = 13.1 min. **^{1}H NMR** (400 MHz, Methanol-d_4) δ 10.03 (d, J = 5.3 Hz, 1H), 9.30 (d, J = 5.3 Hz, 1H), 8.41 (d, J = 9.4 Hz, 1H), 8.26 (dd, J = 9.5, 6.2 Hz, 3H), 8.21 – 8.13 (m, 2H), 8.05 (dd, J = 13.2, 5.3 Hz, 2H), 7.93 (d, J = 6.7 Hz, 3H), 7.82 – 7.61 (m, 9H), 7.56 (m, 13H), 7.02 (s, 1H), 4.68 – 4.52 (m, 2H), 4.15 (d, J = 16.4 Hz, 2H), 4.00 – 3.84 (m, 2H), 3.52 – 3.40 (m, 1H), 3.25 (d, J = 14.8 Hz, 1H), 3.16 – 3.06 (m, 2H), 2.89 (dd, J = 14.8, 9.1 Hz, 1H), 2.50 (d, J = 17.0 Hz, 1H), 2.34 (s, 1H), 2.19 (s, 1H), 2.09 – 1.86 (m, 1H), 1.80 (s, 3H), 1.65 (s, 6H).

[Ru(Ph_2phen)$_2$(Ac-MRADH-NH_2)]Cl_2 ([3]Cl_2)

Ru(Ph_2phen)$_2Cl_2$ (0.025 mmol, 21 mg) was added to a 2-neck 25 mL round-bottom flask, the peptide Ac-MRADH-NH_2 (0.025 mmol, 16.8 mg, as powder) was dissolved in water (2.5 mL) and the pH adjusted to 7.5 by adding 0.5 mM NaOH and 0.1 mM HCl solutions. After adding ethanol (2.5 mL), the peptide solution was degassed by N_2 for 10 min to remove O_2. After the flask had been degassed and put under N_2 by 3 vacuum/N_2 cycles, the deoxygenized peptide solution was injected to the reaction flask. The mixture was then stirred at 60 °C for 3 days. After that, the reaction solution was cooled down to room temperature, ethanol was removed by rotary evaporation, and the suspension filtered and washed by cold water. The filtrate was then freeze-dried, to afford a reddish powder. Further purification was accomplished by HPLC. The purification was realized on a 250 x 21.2 mm Jupiter® 4 µm Proteo 90 Å C12 column using a Thermo Scientific UHPLC system. The gradient was controlled by four pumps. The mobile phase consisted of mixture of H_2O containing 0.1% v/v formic acid (A phase) and acetonitrile containing 0.1% v/v formic acid (B phase). The gradient for [**3**]Cl_2 preparative separation was 25-40% phase B/phase A, for 20 min. The fractions were monitored by four UV detector (214 nm, 290 nm, 350 nm, 450 nm) and the flow rate was 14 mL/min. Compound were collected at UV-detector 290 nm. After lyophilization, two enantiomers were obtained as orange-red powder (the yield for Λ-[**3**]Cl_2 and Δ-[**3**]Cl_2 was around 6% and 18%, respectively) Δ-[**3**]Cl_2 **HR-MS** *found* (*calc*): *m/z* = 478.8257 (478.8256 for [Δ-[**3**]+H]$^{3+}$, [$C_{74}H_{76}N_{15}O_8RuS$]$^{3+}$. **HPLC** (10-90% phase B/phase A, 25 min): t_R = 13.3 min.

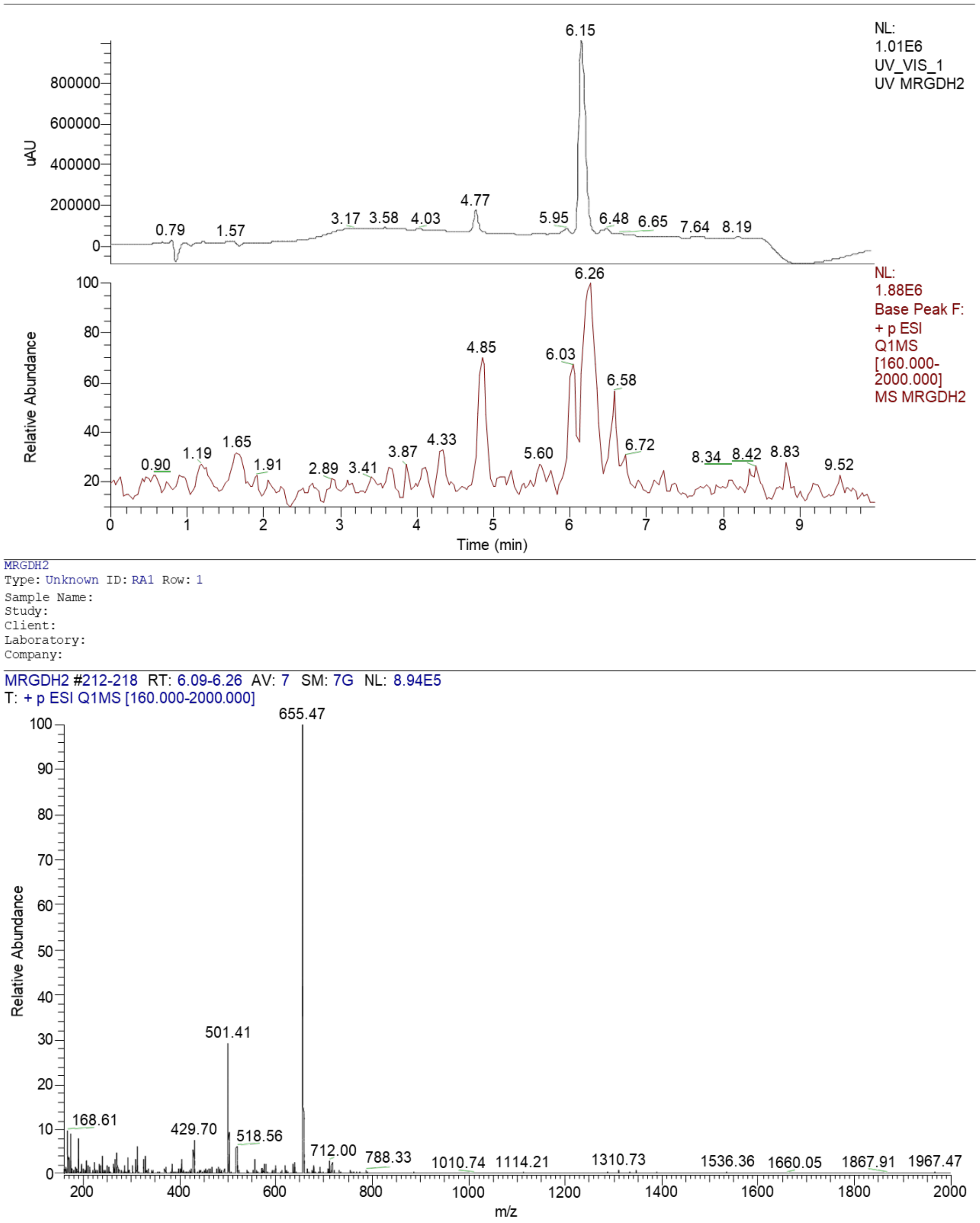

Figure S1. LC-MS chromatogram of peptide Ac-MRGDH-NH_2. MS: calculated 655.3, found 655.5.

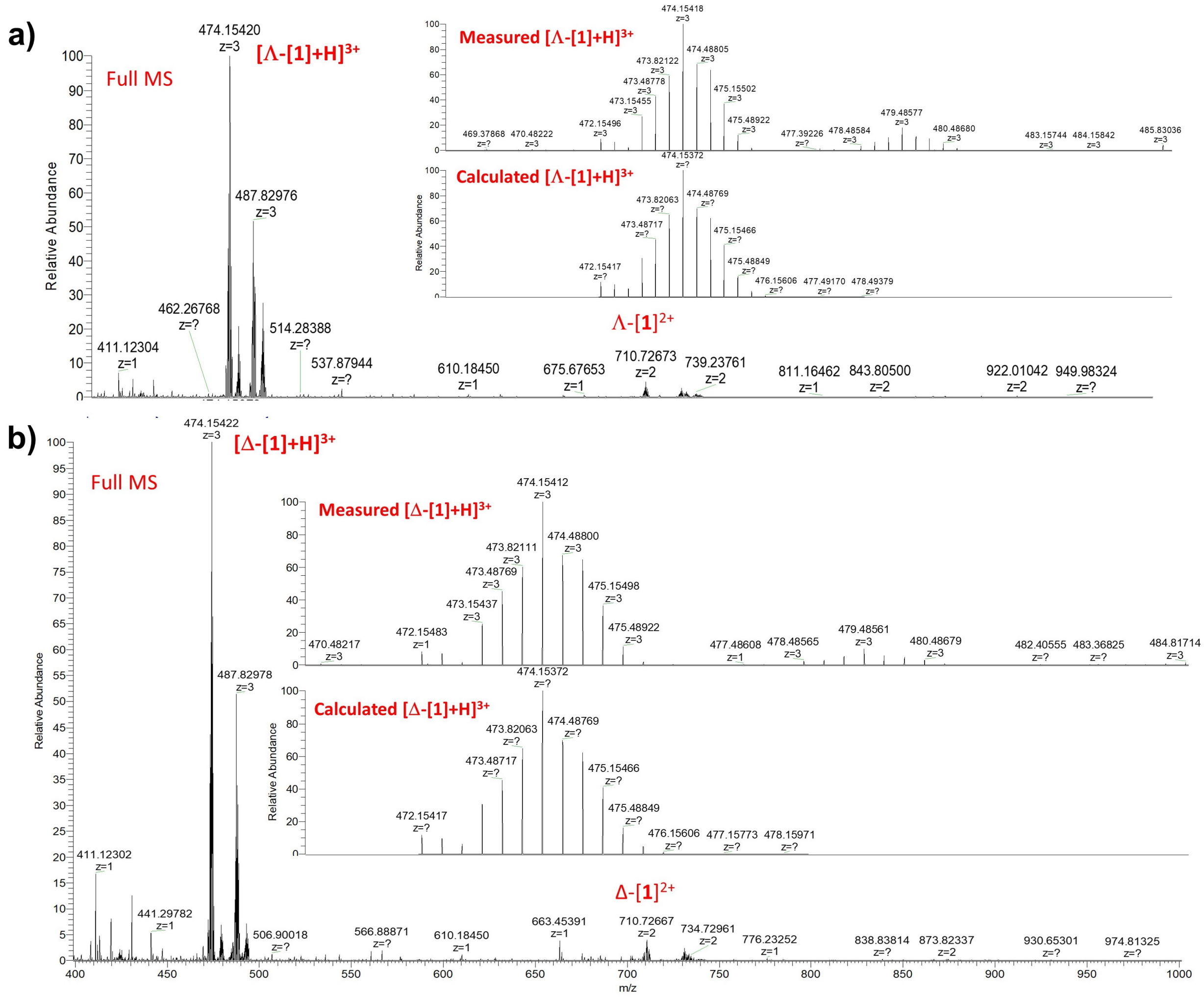

Figure S2. High resolution mass spectra (HR-MS) of conjugates Λ-[**1**]Cl_2 (a) and Δ-[**1**]Cl_2 (b) in acetonitrile after purification. Calc. m/z for [**M**]$^{2+}$ = 710.7260 and [**M**+H]$^{3+}$ = 474.1537).

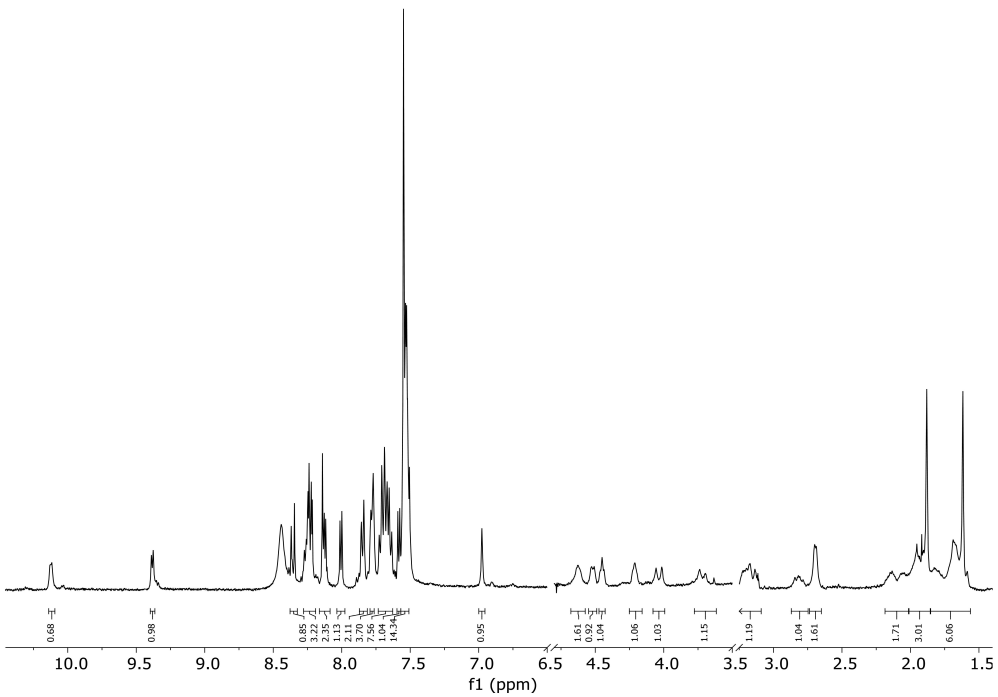

Figure S3. ^{1}H NMR (400 MHz, Methanol-d$_4$) spectra of Λ-[**1**]Cl_2.

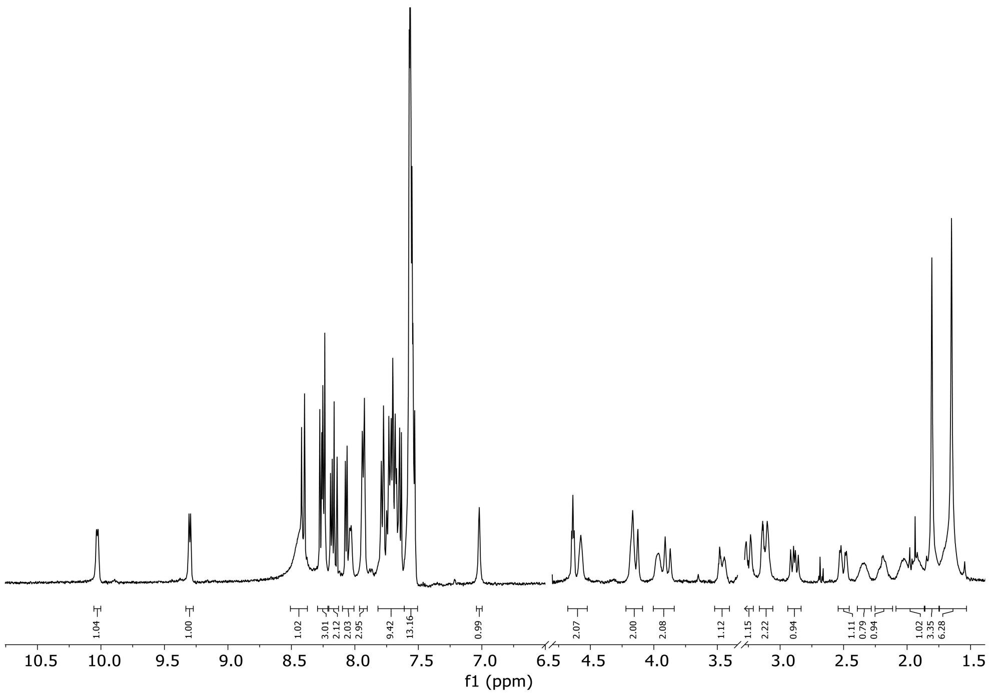

Figure S4. ^{1}H NMR (400 MHz, Methanol-d$_4$) spectra of Δ-[**1**]Cl_2.

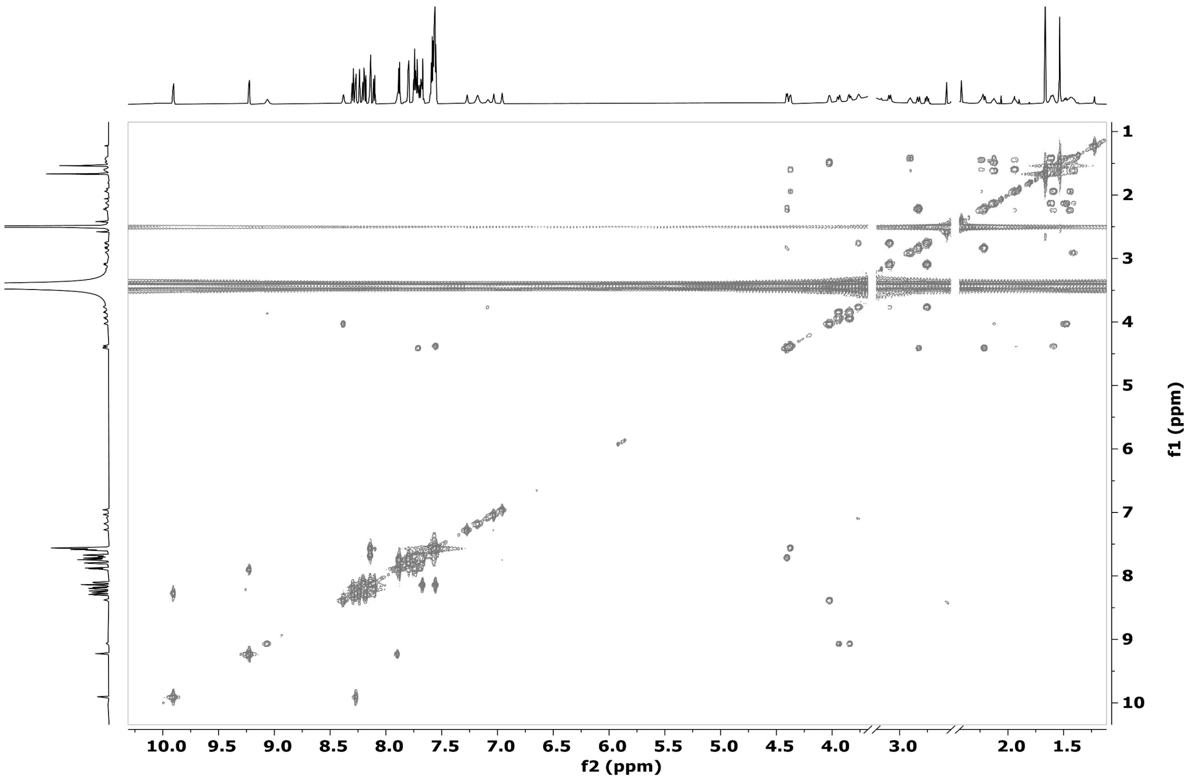

Figure S5. COSY (850 MHz, DMSO-d_6) spectra of Δ-[**1**]Cl_2.

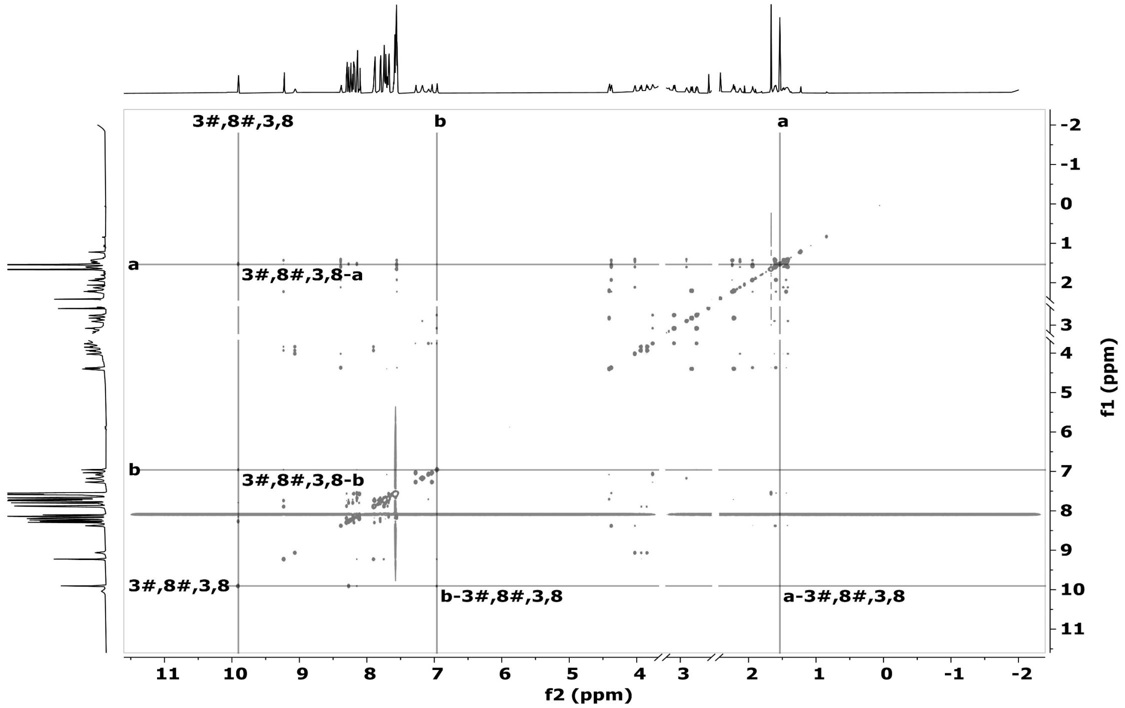

Figure S6. NOESY (850 MHz, DMSO-d_6) spectra of Δ-[**1**]Cl_2.

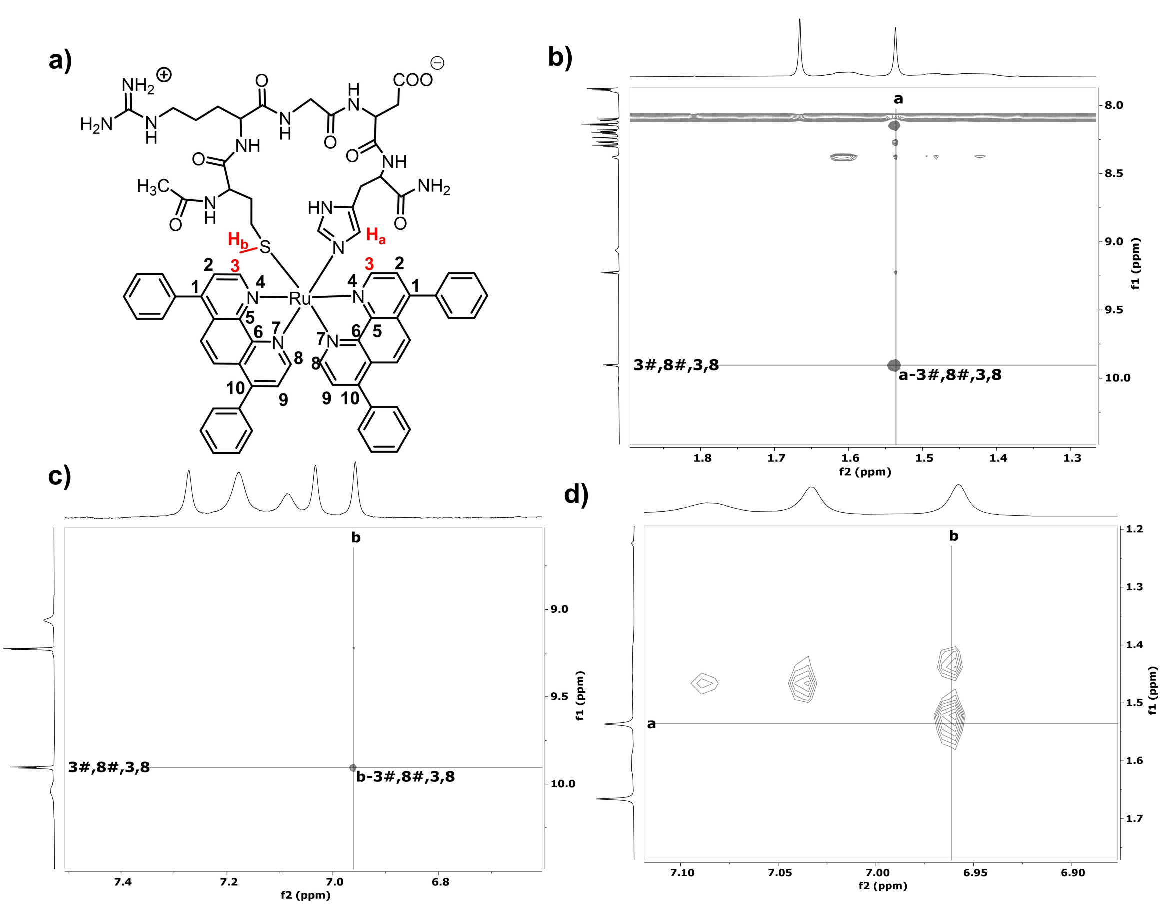

Figure S7. Partial region of NOESY (850 MHz, DMSO-d_6) spectra of Δ-[**1**]Cl_2.

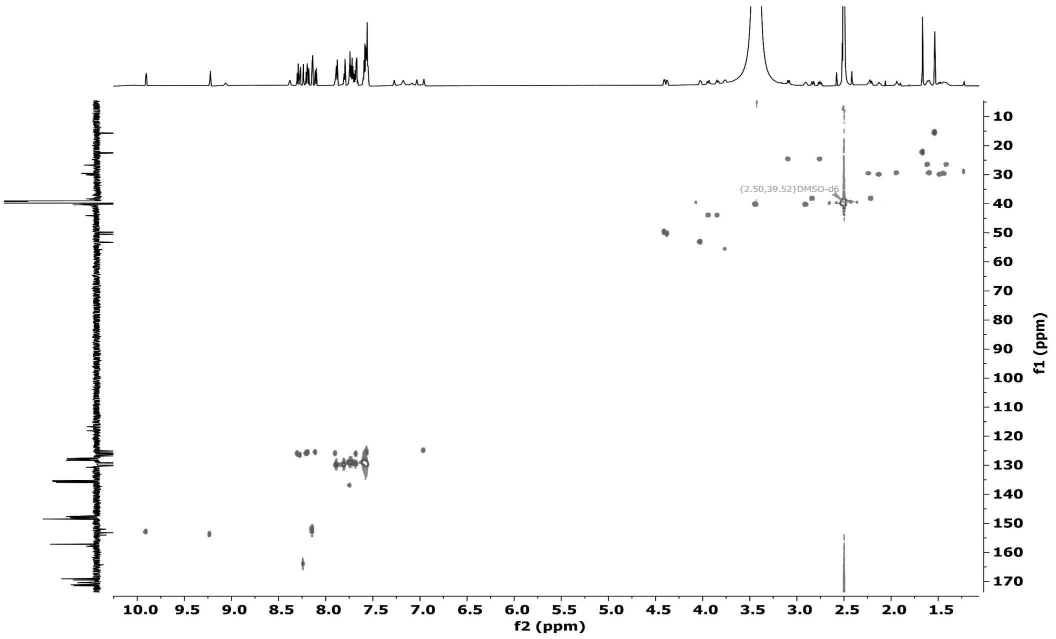

Figure S8. HSQC (850 MHz, DMSO-d_6) spectra of Δ-[**1**]Cl_2.

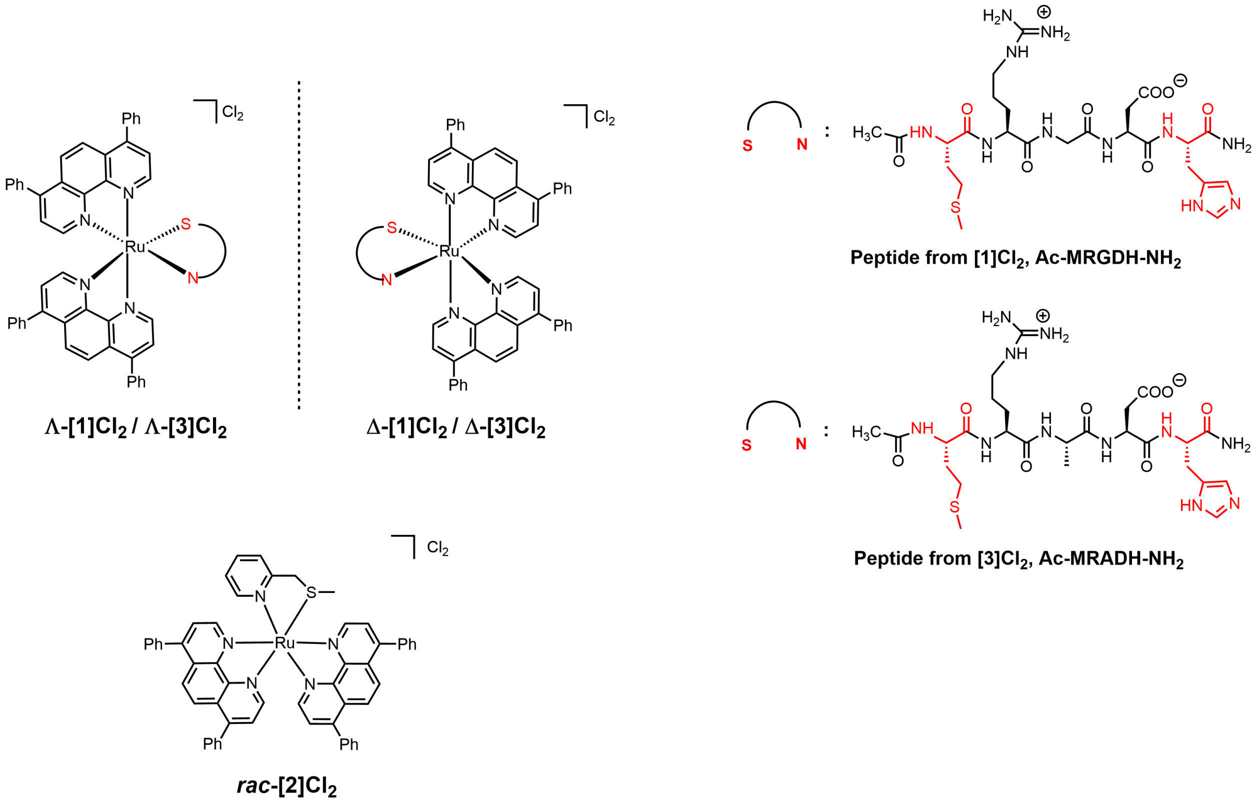

Figure S9. Molecular structures of Λ-[**1**]Cl_2, Δ-[**1**]Cl_2, [**2**]Cl_2, Λ-[**3**]Cl_2 and Δ-[**3**]Cl_2.

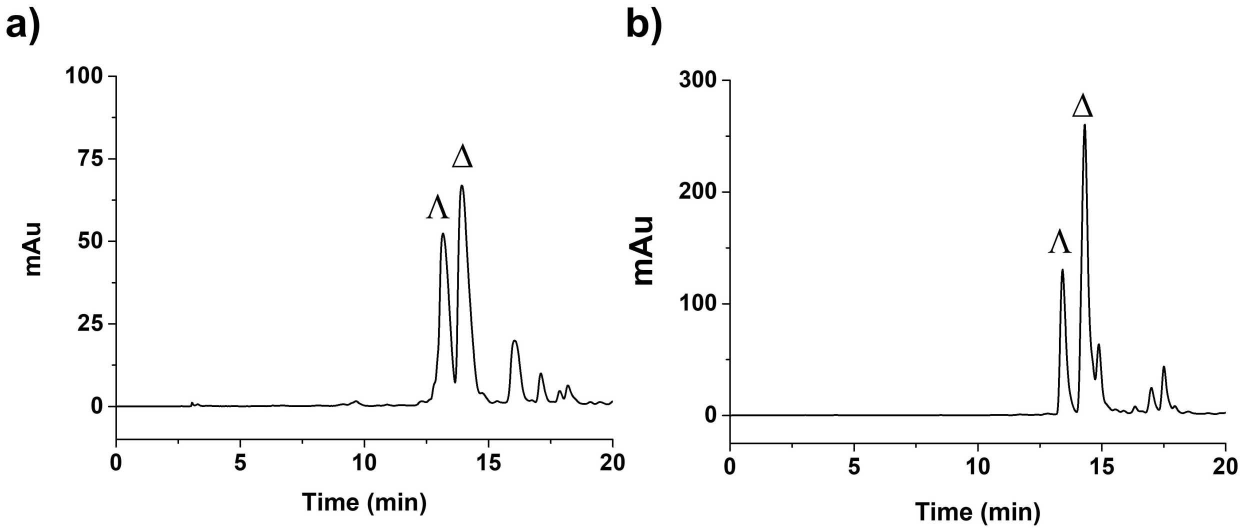

Figure S10. a) HPLC trace of crude product during preparation of [**1**]Cl_2, i.e., before purification. Gradient: 30-40% phase B/phase A, 20 min, flow rate = 14 mL/min, detector UV channel = 290 nm. b) HPLC trace of crude product during preparation of [**3**]Cl_2, i.e., before purification. Gradient: 25-40% phase B/phase A, 20 min, flow rate = 14 mL/min, detector UV channel = 290 nm.

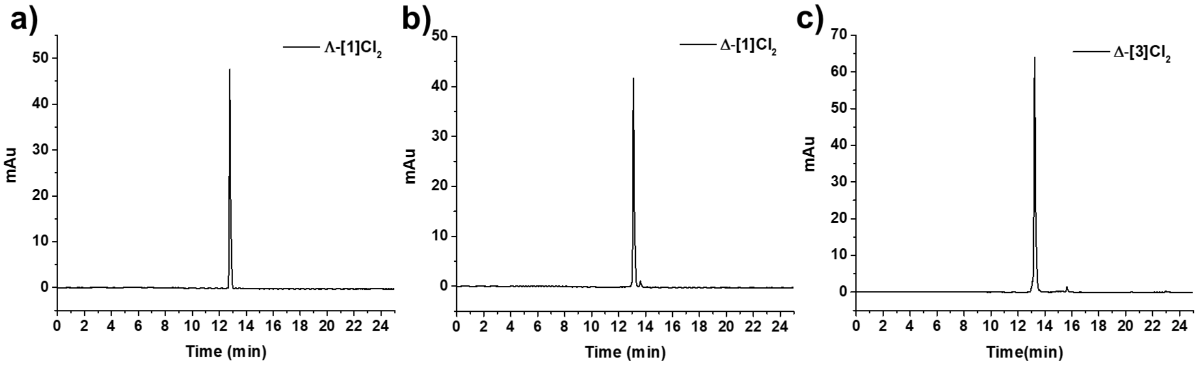

Figure S11. HPLC trace of conjugates Λ-[**1**]Cl_2 (a, t_R = 12.8 min), Δ-[**1**]Cl_2 (b, t_R = 13.1 min) and Δ-[**3**]Cl_2 (c, t_R = 13.3 min) after purification. Gradient: 10-90% phase B/phase A, 25 min, detector UV channel = 290 nm.

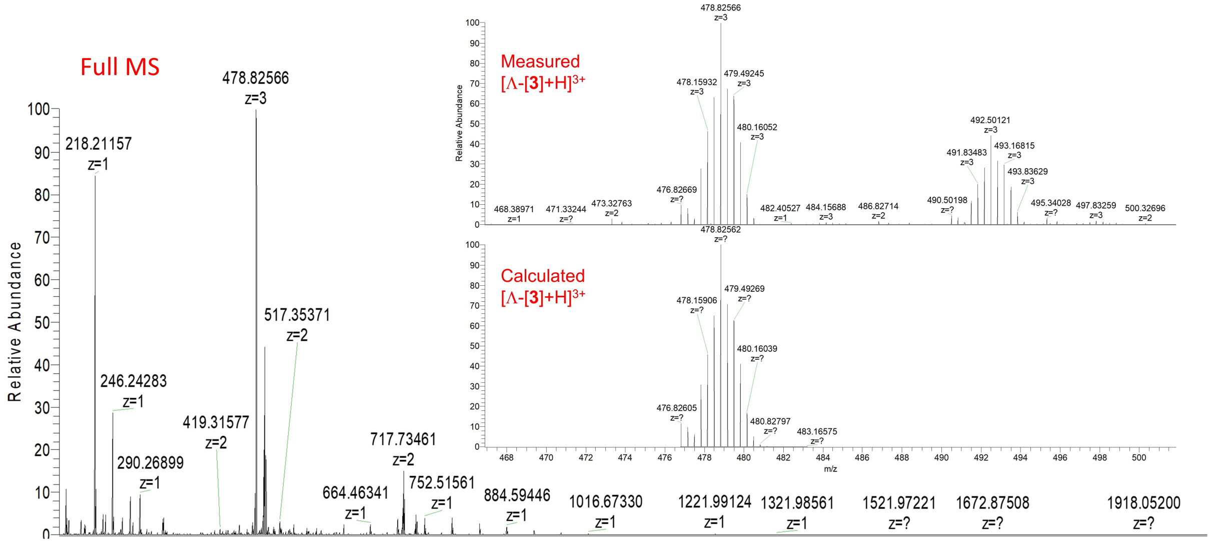

Figure S12. High resolution mass spectra (HR-MS) for conjugates Δ-[**3**]Cl_2 in acetonitrile after purification. (calc. m/z for $[\mathbf{M}]^{2+}$ = 717.7338 and $[\mathbf{M}+H]^{3+}$ = 478.8256).

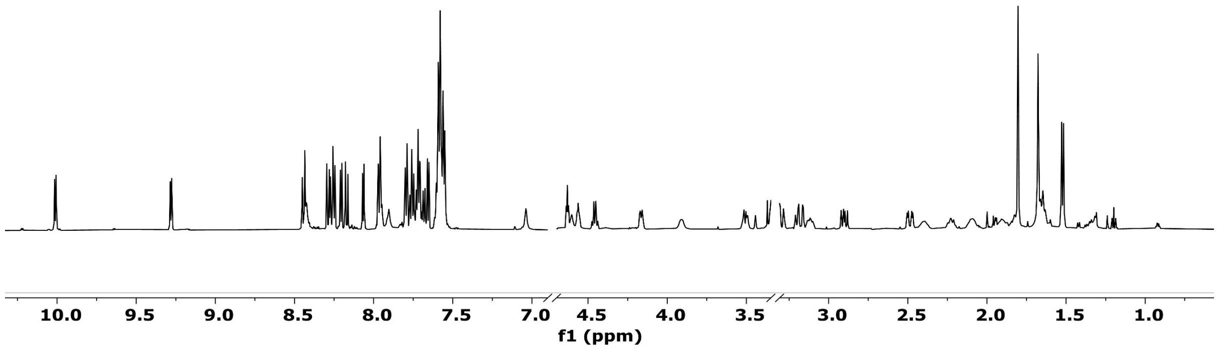

Figure S13. ^{1}H NMR (600 MHz, CD_3OD) spectra of Δ-[3]Cl_2.

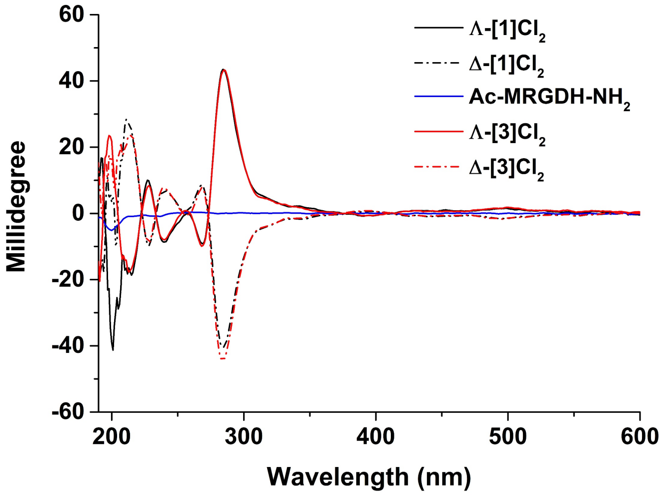

Figure S14. CD spectra comparison of free peptide Ac-MRGDH-NH_2, Λ-[**1**]Cl_2, Δ-[**1**]Cl_2, Λ-[**3**]Cl_2 and Δ-[**3**]Cl_2 in aqueous solution (25 μM).

3 Photochemistry

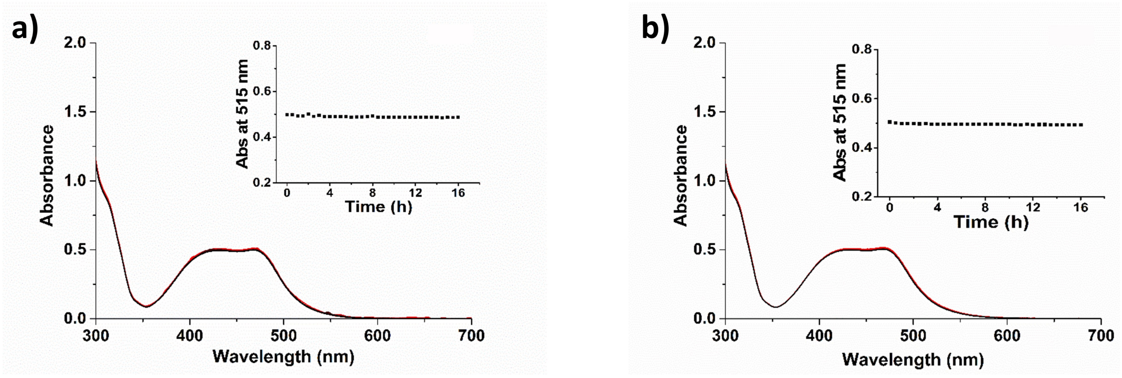

Figure S15. Evolution of the UV-vis spectra of Λ-[**1**]Cl_2 (a) and Δ-[**1**]Cl_2 (b) (40 μM) in 1:1 H_2O:CH_3CN when kept in the dark at room temperature.

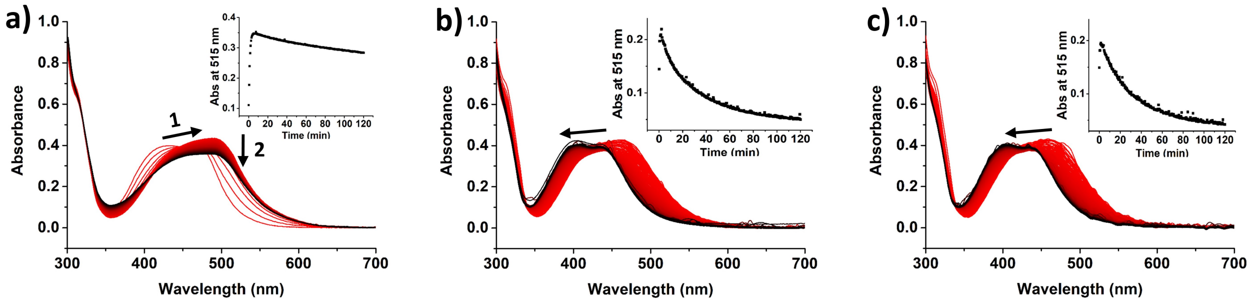

Figure S16. Evolution of the UV-vis spectra of solutions of Λ-[**1**]Cl_2 (25 μM) in H_2O (a) and in a H_2O/ACN (1:1 v/v) mixture (b); or (c) of Δ-[**1**]Cl_2 (25 μM) in a H_2O/ACN (1:1 v/v) mixture, respectively, under green light (515 nm, 2 h, intensity:4.0 mW/cm^2) irradiation.

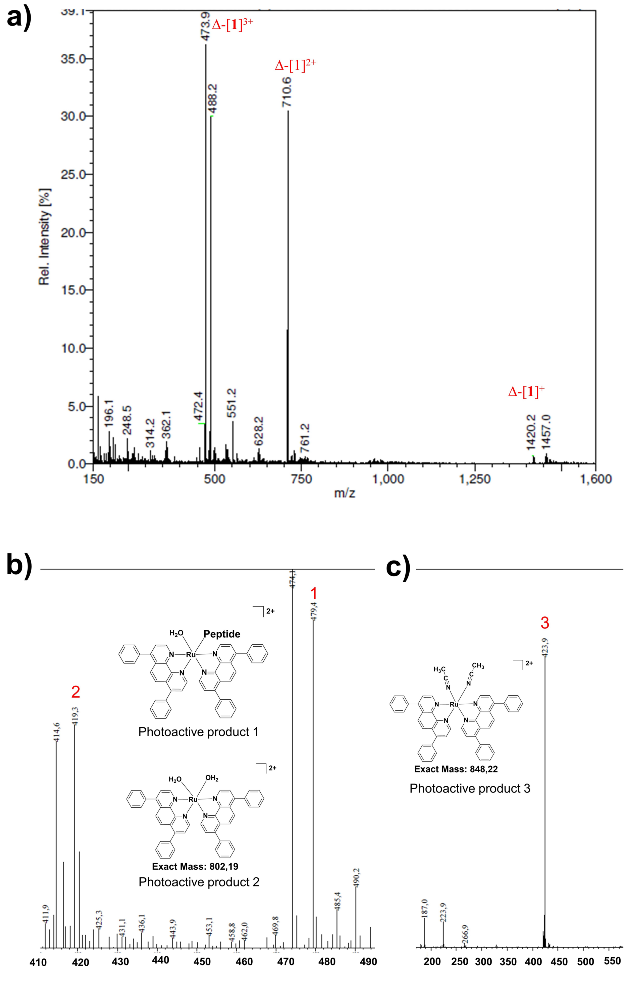

Figure S17. Mass spectra of Δ-[**1**]Cl_2 in H_2O under dark (a), or after green light (515 nm) irradiation by 2 h in H_2O (b) and H_2O:CH_3CN (1:1 v/v) (c). Photoactive product 1: $[Ru(Ph_2phen)_2(Ac\text{-}MRGDH\text{-}NH_2)(H_2O)]^{3+}$ (calc. m/z for $[M]^{3+}$ = 479.4); photoactive product 2: $[Ru(Ph_2phen)_2(H_2O)_2]^{2+}$ plus H_2O (calc. m/z = 419.1); Photoactive product 3: $[Ru(Ph_2phen)_2(ACN)_2]^{2+}$ (calc. m/z= 424.1).

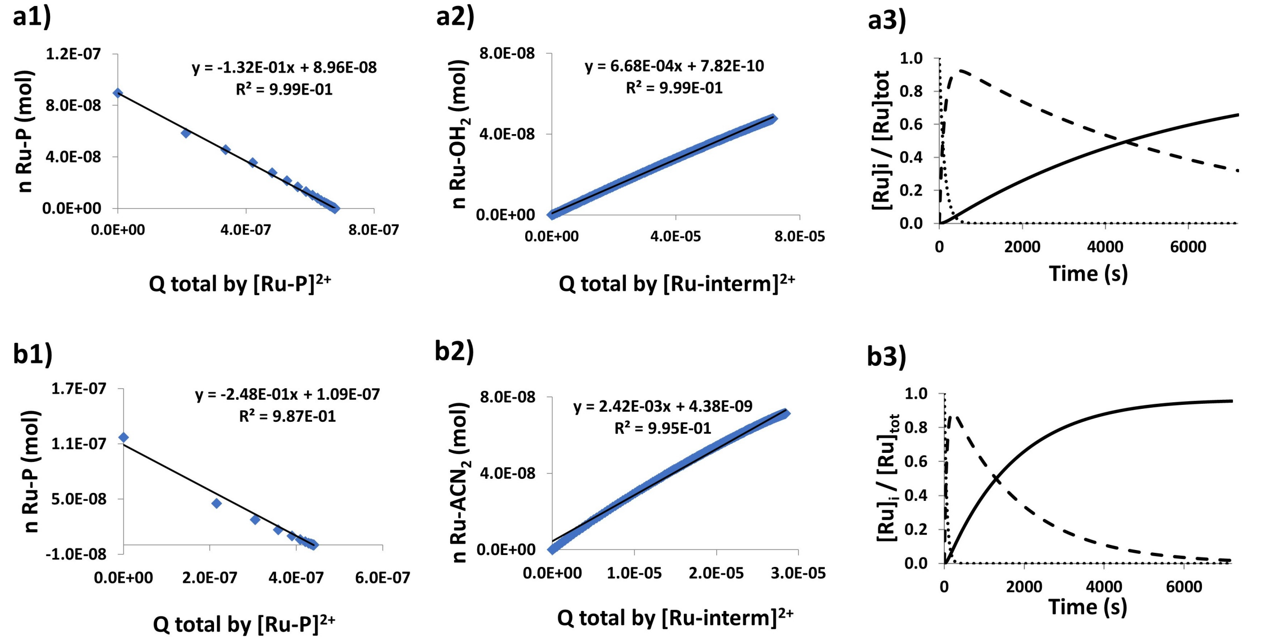

Figure S18. Fitting of the UV-vis time evolution when Λ-[**1**]Cl_2 is irradiated with green light (515 nm, 4.0 mW/cm^2) in H_2O (a1-a3) or in H_2O:CH_3CN (1:1 v/v) (b1-b3). (1) Amount of Ru-peptide reagent (Λ-[**1**]Cl_2) plotted *vs*. the number of photons Q absorbed by Λ-[**1**]Cl_2 since t=0 (in mol). (2) Amount of photosubstituted product (either Ru-OH_2 or Ru-ACN_2) generated *vs*. the number of photons Q absorbed by the η^1-intermediate. (3) Evolution of the relative concentrations of Ru-peptide reactant (dotted line), η^1-intermediate (dashed line) and photoproduct (solid line) according to global fitting using Glotaran. The absolute value of the slopes of the trendlines in 1) and 2) correspond to the quantum yields for the two consecutive steps in the two photosubstitution reactions, one leading to Ru-OH_2 (in water), the other leading to Ru-ACN_2 (in H_2O:CH_3CN).

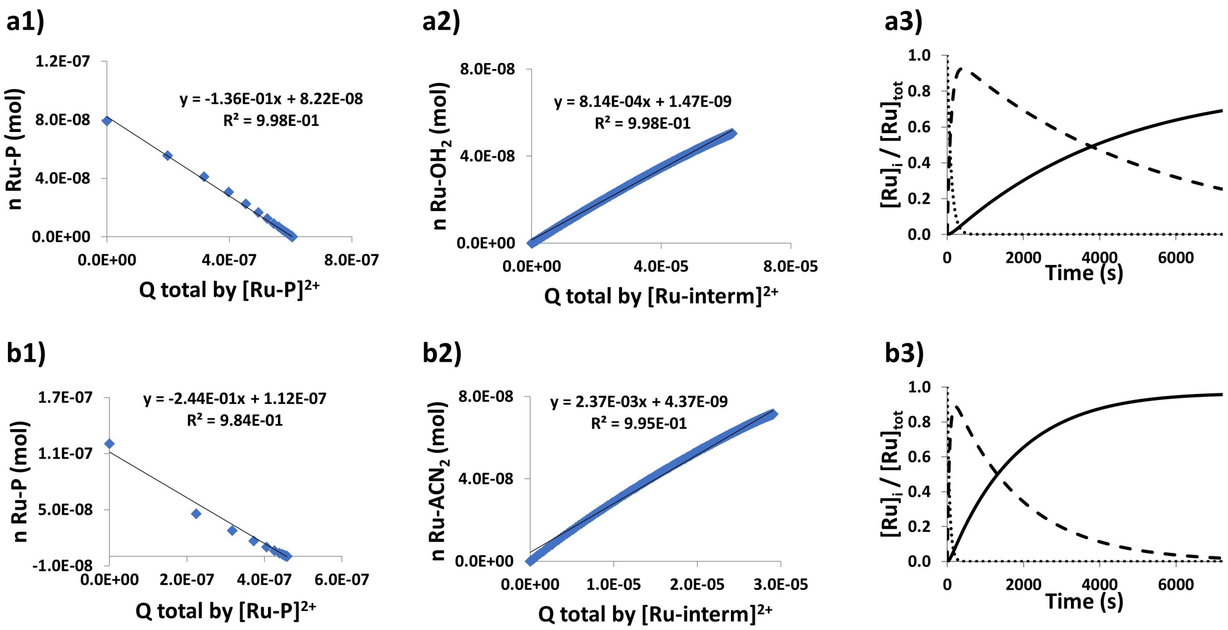

Figure S19. Fitting of the UV-vis time evolution when Δ-[**1**]Cl_2 is irradiated with green light (515 nm, 4.0 mW/cm^2) in H_2O (a1-a3) or in H_2O:CH_3CN (1:1 v/v) (b1-b3). (1) Amount of Ru-peptide reagent (Δ-[**1**]Cl_2) plotted *vs*. the number of photons Q absorbed by Δ-[**1**]Cl_2 since t = 0 (in mol). (2) Amount of photosubstituted product (either Ru-OH_2 or Ru-ACN_2) generated *vs*. the number of photons Q absorbed by the η^1-intermediate. (3) Evolution of the relative concentrations of Ru-peptide reactant (dotted line), η^1-intermediate (dashed line) and photoproduct (solid line) according to global fitting using Glotaran. The absolute value of the slopes of the trendlines in 1) and 2) correspond to the quantum yields for the two consecutive steps in the two photosubstitution reactions, one leading to Ru-OH_2 (in water), the other leading to Ru-ACN_2 (in H_2O:CH_3CN).

Table S1. Quantum yields for step 1 (Φ_{PS1}) and step 2 (Φ_{PS2}) of the photosubstitution reaction in H_2O or 50% CH_3CN in H_2O.

compound	Solvent	Φ_{PS1}	Φ_{PS2}
Λ-[**1**]Cl_2	H_2O	0.13	0.0007
	H_2O: CH_3CN (1:1 v/v)	0.25	0.0024
Δ-[**1**]Cl_2	H_2O	0.14	0.0008
	H_2O: CH_3CN (1:1 v/v)	0.24	0.0024

4 Nanoaggregate characterization

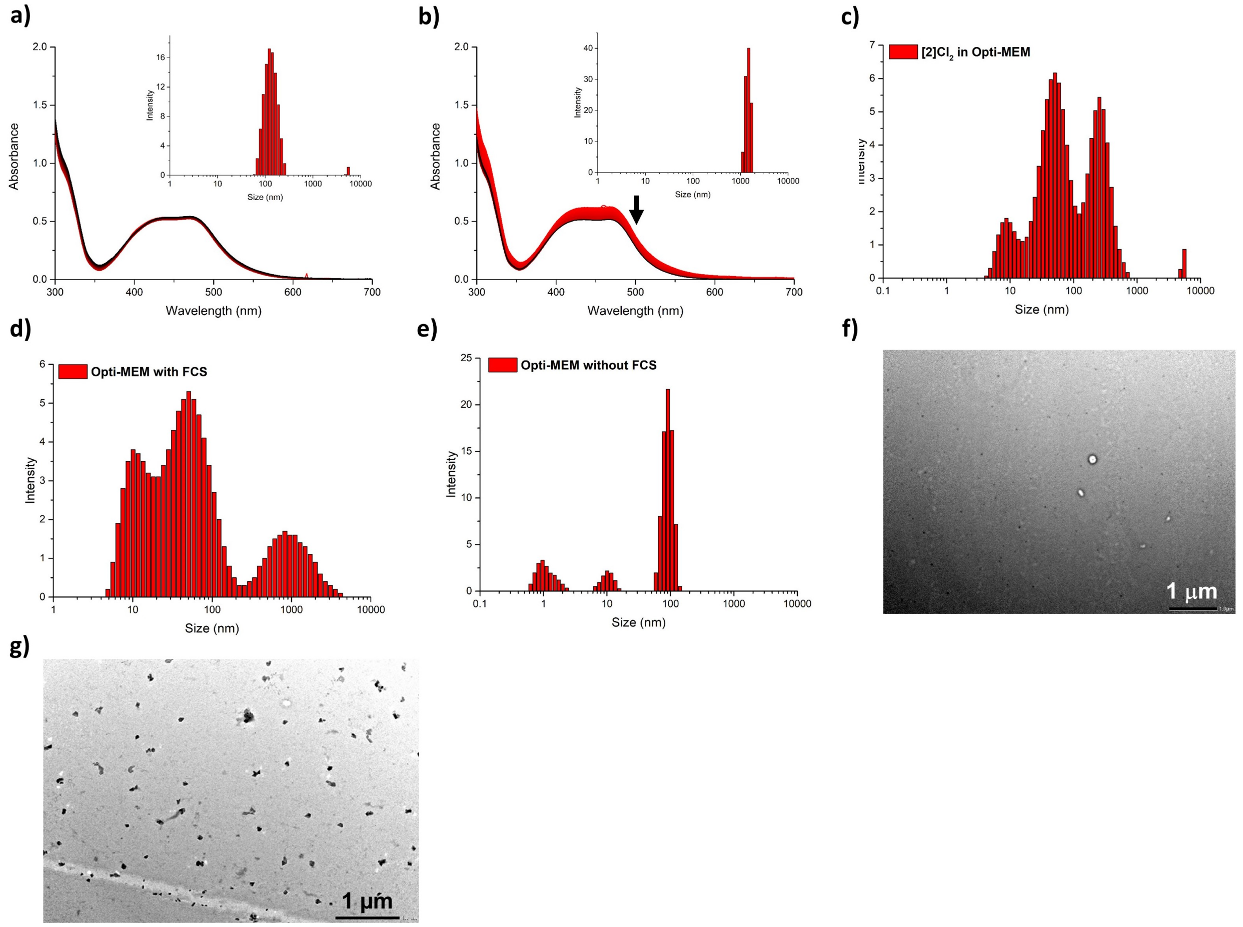

Figure S20. Time evolution of the UV-vis absorption spectra of Λ-[**1**]Cl_2 (50 μM) solution in opti-MEM medium with (a) and without (b) 2.5% FCS in the dark (24 h). Insert: corresponding DLS results at t = 24 h. c) DLS of **[2]**$\mathbf{Cl_2}$ (50 μM) in opti-MEM with 2.5% FCS in the dark at t = 24 h. d) and e) DLS results of opti-MEM medium samples with and without 2.5% FCS, respectively. (f) TEM image of only Opti-MEM containing 2.5% FCS. (g) TEM image of Δ-[**1**]Cl_2 (50 μM) solution in Opti-MEM containing 2.5% FCS.

5 Cytotoxicity study without washing: dose-response curves

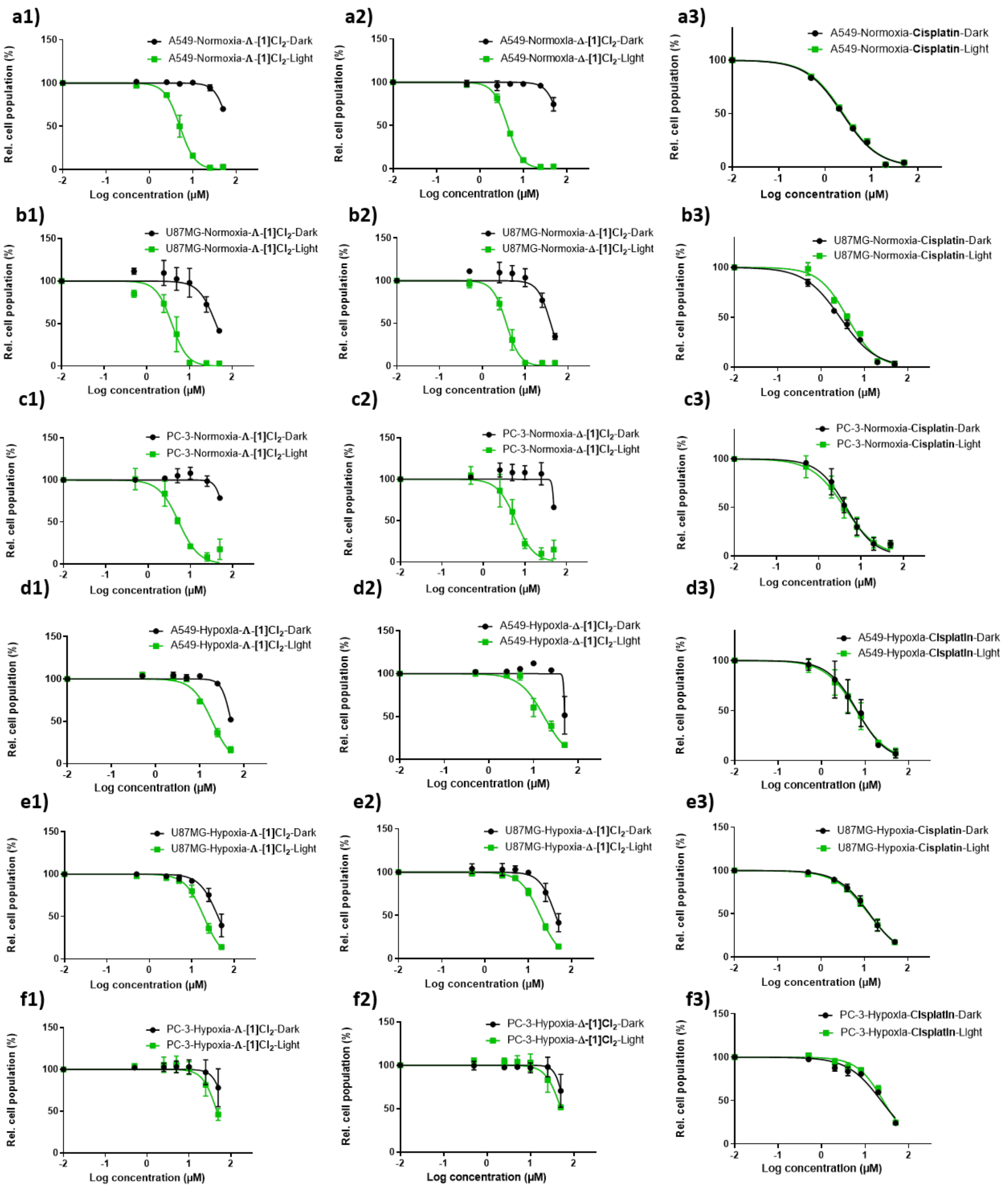

Figure S21. Dose-response curves for Λ-[**1**]Cl₂ (1), Δ-[**1**]Cl₂ (2) and cisplatin (3), in normoxic 2D monolayers of A549 (a), U87MG (b), or PC-3 (c), and in hypoxic 2D monolayers of A549 (d) , U87MG (e), or PC-3 (f) cells. Normoxia: 37°C, 21% O_2 and 5% CO_2; Hypoxia: 37°C, 1% O_2 and 5% CO_2; black curve: dark condition; green curve: irradiated with green light (Normoxia: 520 nm, 10.9 mW/cm^2, 13.1 J/cm^2, 20 min and hypoxia: 520 nm, 7.22 mW/cm^2, 13.1 J/cm^2, 30 min). Every group was conducted in triplicate, error bars represent 95% confidence intervals.

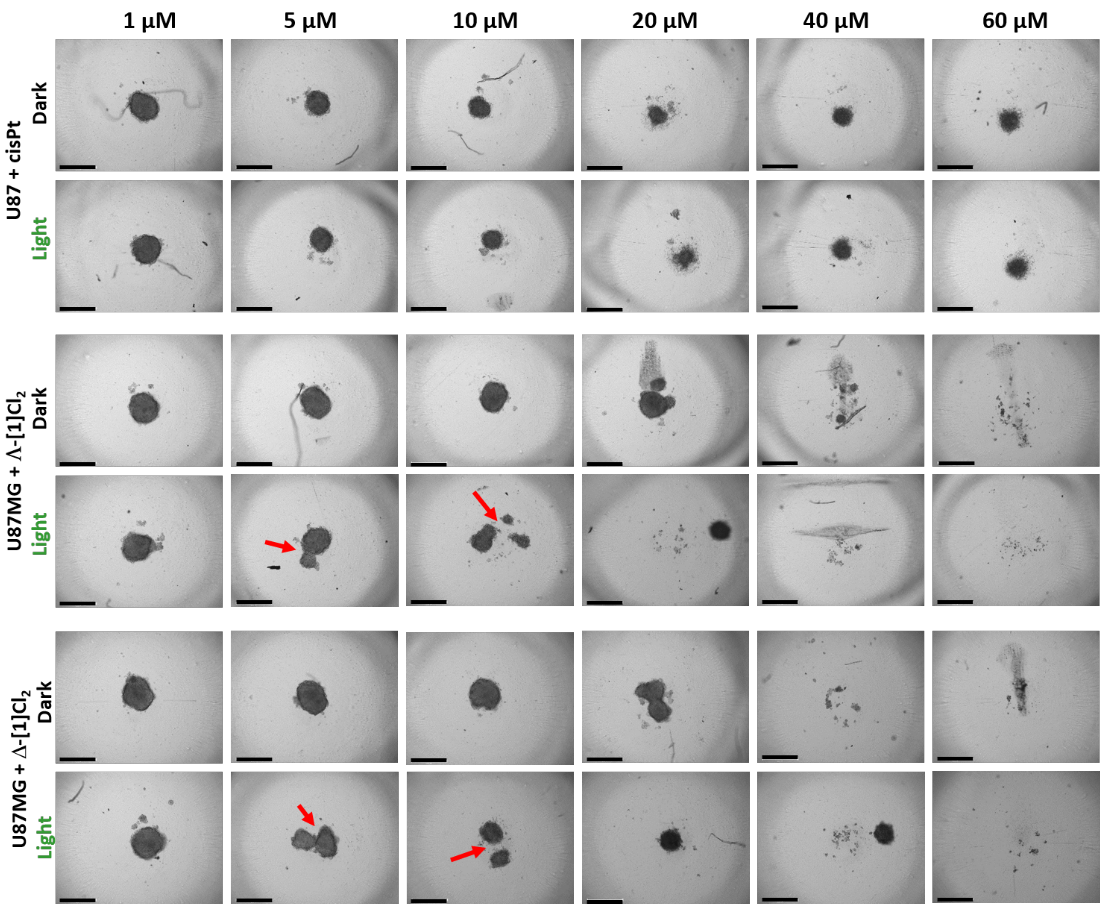

Figure S22. Bright field images of U87MG 3D tumor spheroids treated with different concentrations of cisplatin, Λ-[**1**]Cl_2 or Δ-[**1**]Cl_2 and left in the dark or irradiated with green light (520 nm, 13.1 J/cm^2). Scale bar = 500 μm.

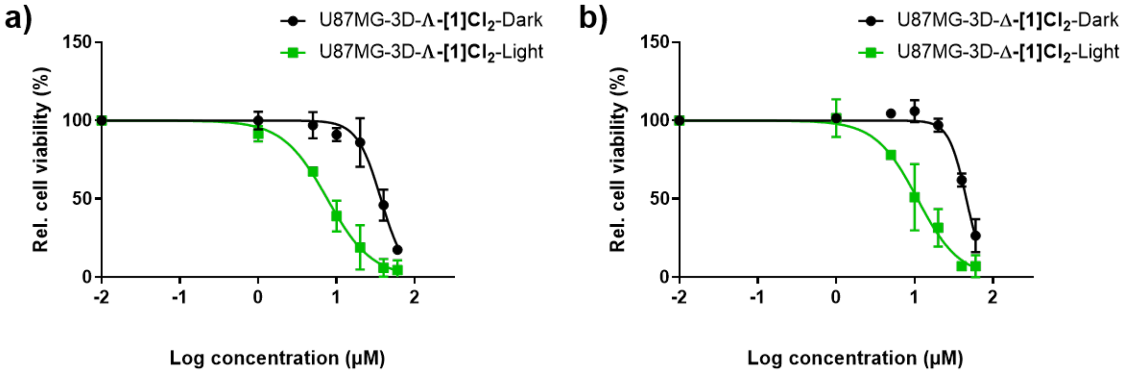

Figure S23. Dose-response curves for U87MG 3D tumor spheroids incubated with complex Λ-[**1**]Cl_2 or Δ-[**1**]Cl_2 in the dark (black) or irradiated with green light (in green, 520 nm, 13.1 J/cm^2).

6 $^1O_2$ generation and intracellular ROS generation

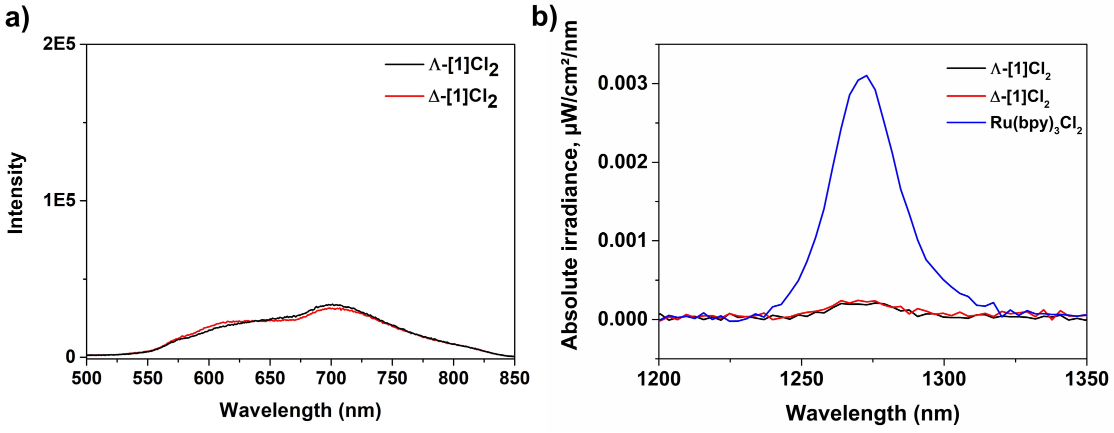

Figure S24. (a) Emission spectra of Λ-[**1**]Cl_2 and Δ-[**1**]Cl_2 (50 μM, λ_{ex} = 480 nm) in MeOD and (b) normalized near infrared spectroscopy (NIR) emission from $^1O_2$ generated by Ru(bpy)$_3$$Cl_2$, Λ-[**1**]$Cl_2$, Δ-[**1**]$Cl_2$ in MeOD, conditions: 298 K, 50 mW laser power, 450 nm.

Table S2. Determination of singlet oxygen quantum yields (Φ_Δ) corresponding to Figure S**24**.

	**Λ-[1]Cl₂**	**Λ-[1]Cl₂**	**Ru(bpy)$_3$Cl$_2$ [a]**
Absorbance at 450 nm	0.112	0.113	0.089
Φ_Δ	0.046 ± 0.021	0.059 ± 0.034	0.73

[a] The prototypical [Ru(bpy)$_3$]Cl_2 complex was used as a reference (Φ_Δ^{ref} = 0.73).[2]

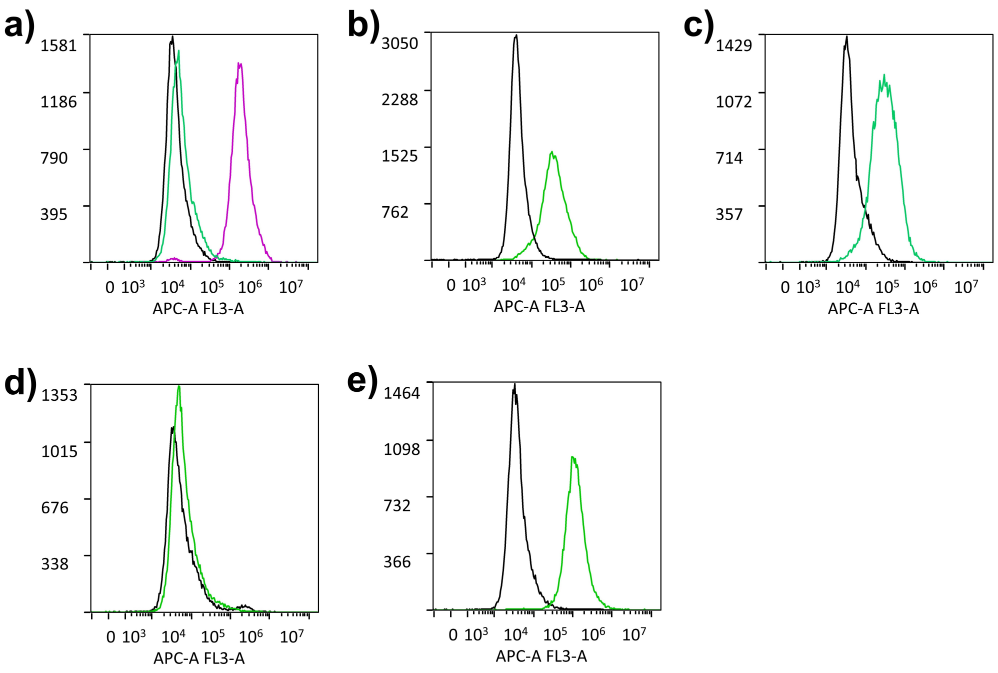

Figure S25. Reactive Oxygen Species generation in U87MG cells according to FACS analysis using CellROX™ Deep Red Reagent as ROS probe, after treatment with medium only or tBHP (a, 250 μM, positive control), and complexes (15 μM, 24 h) Λ-[**1**]Cl_2 (b), Δ-[**1**]Cl_2 (c), cisplatin (d) or Rose Bengal (e) in the dark or after light irradiation (515 nm, 13.1 J/cm^2, 20 min). Dark group (black curve), light group (green curve) and tBHP (purple curve) samples are as shown. X-axis represents the ROS probe's intensity detected by APC-A channel of FACS, higher value means higher ROS generation and Y-axis represents counted cell numbers.

Table S3. Mean fluorescence intensity (×10^3) of cells treated with complex Λ-[**1**]Cl_2, Δ-[**1**]Cl_2, cisplatin, Rose Bengal or [2]Cl_2 (15 μM, 24 h) under the dark and green light and then with CellROX™ Deep Red Reagent as ROS probe. [a, b]

	**TBHP**[*c*]	**Control**	**Λ-[1]Cl_2**	**Δ-[1]Cl_2**	**cisplatin**	**Rose Bengal**
Dark	*232.8*	5.54	5.83	5.86	13.9	6.08
Light		9.64	41.0	48.3	11.2	145.7
Ratio of L/D		1.74	7.03	8.24	0.81	24.0

[a] ROS generation measurement conditions: U87MG cell lines, [b] Λ-[**1**]Cl_2, Δ-[**1**]Cl_2, cisplatin and Rose Bengal: 15 μM; [c] tBHP (tert-Butyl hydroperoxide, positive control): 250 μM; ROS indicator: 1000x dilution (Cellular ROS Assay Kit (Deep Red) ab186029).

7 Detection of secondary photoproducts by FACS in U87MG treated with Δ-[1]Cl_2 and light

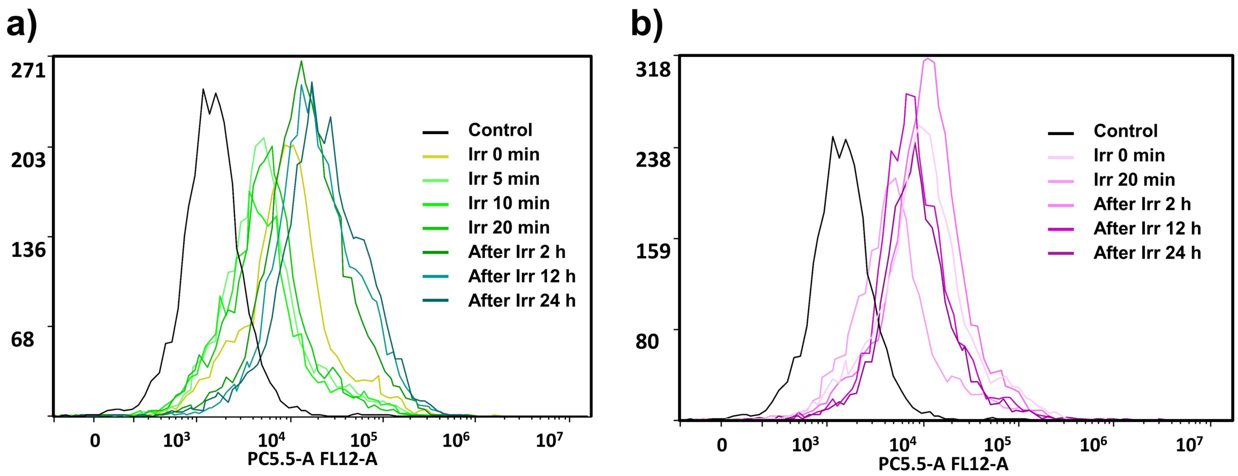

Figure S26. Flow cytometry histograms of U87MG cells after treatment with **Δ-[1]Cl_2** (10 μM, 6 h) and then washed, either irradiated with green light for 0, 5, 10 or 20 min, or put back into normoxic incubator for 2, 12 or 24 h after 20 min's irradiation (a, 520 nm, intensity = 10.9 mW/cm^2). Non-irradiated cells at the similar time points are shown in b. Cells treated only with medium were used as control, X-axis represents the fluorescence intensity detected using the PC5.5 channel (488 nm excitation, 650 ± 50 nm emission) of the FACS apparatus.

8 Apoptosis assay by FACS

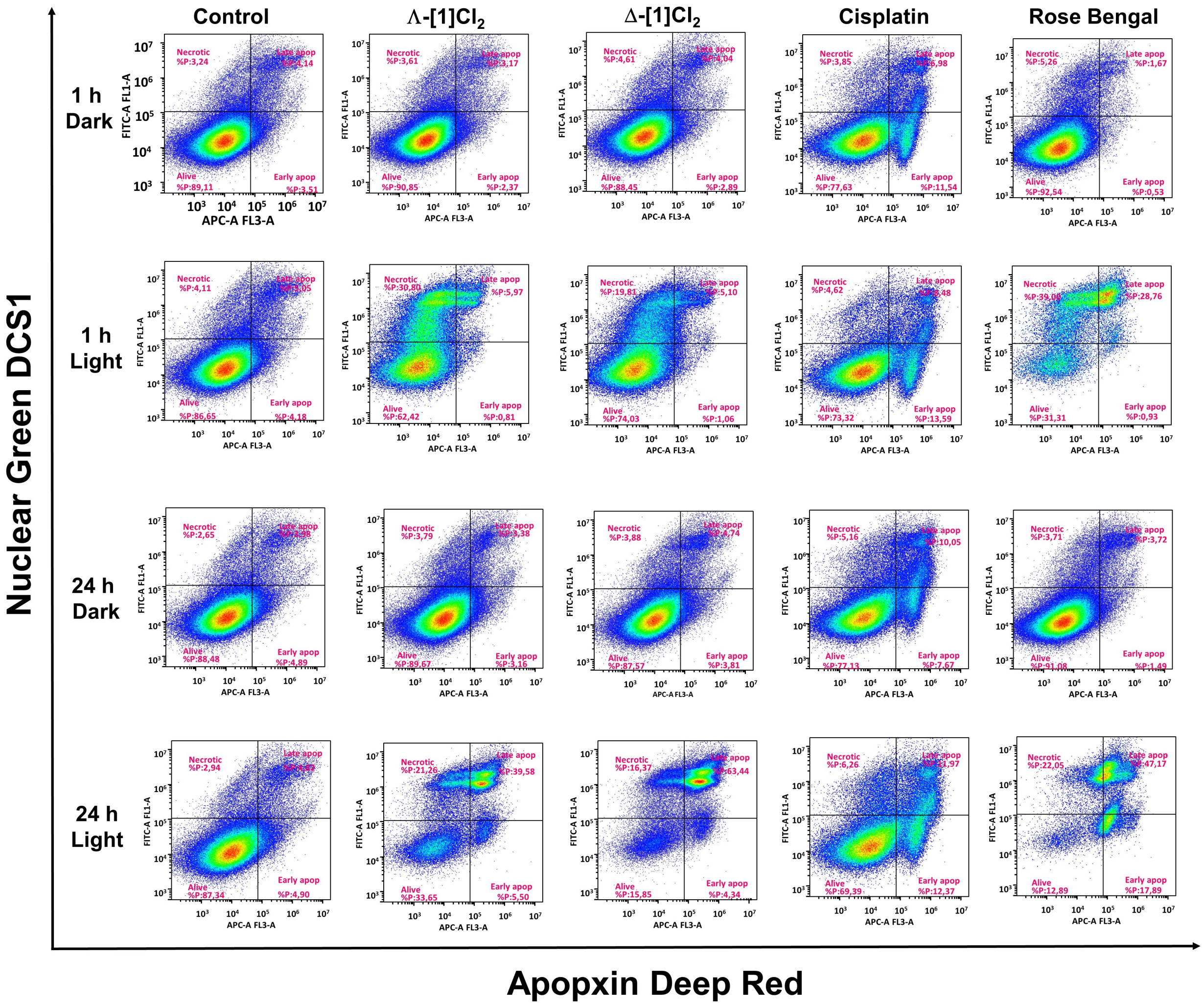

Figure S27. Apopxin/Nuclear Green double staining FACS data from U87MG cells upon treatment with medium (control), Λ-[**1**]Cl₂, Δ-[**1**]Cl₂, cisplatin, or Rose Bengal (20 μM) in the dark or after green light irradiation for 1 h or 24 h by flow cytometry using ab176749 Apoptosis/Necrosis Assay Kit. Conditions: normoxia, dark or irradiated with green light (520 nm, 13.1 J/cm^2). Axes are logarithmic and consistent, showing Nuclear Green DCS1 (probe of necrosis) fluorescence values detected by FITC channel of FACS on the Y-axis, and Apopxin Deep Red (probe of apoptosis) fluorescence values detected by APC-A channel of FACS on the X-axis.

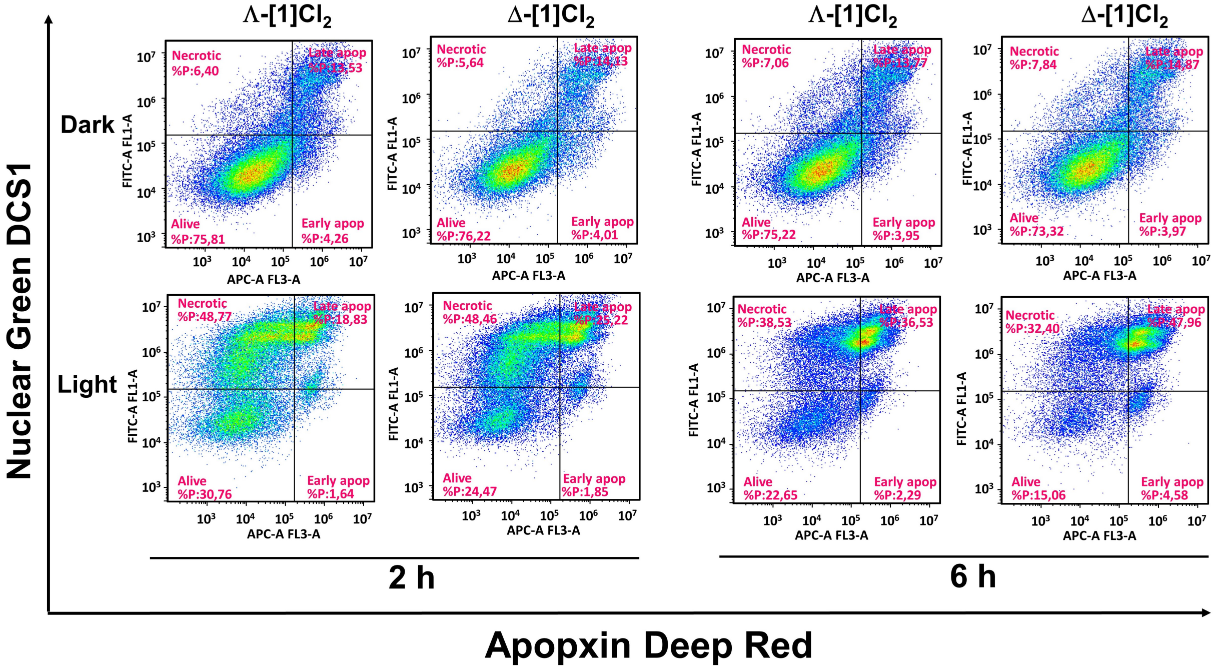

Figure S28. Apopxin/Nuclear Green double staining FACS data from U87MG cells upon treatment with Λ-[**1**]Cl_2, Δ-[**1**]Cl_2 (20 μM) in the dark or after green light irradiation for 2 h or 6 h by flow cytometry using ab176749 Apoptosis/Necrosis Assay Kit. Conditions: normoxia, dark or irradiated with green light (520 nm, 13.1 J/cm^2). Axes are logarithmic and consistent, showing Nuclear Green DCS1 (probe for necrosis) fluorescence values detected by FITC channel of the FACS machine on the Y-axis, and Apopxin Deep Red (probe for apoptosis) fluorescence values detected by APC-A channel of the FACS machine on the X-axis.

9 Integrin $\alpha_v\beta_3$ and $\alpha_v\beta_5$ expression by FACS analysis

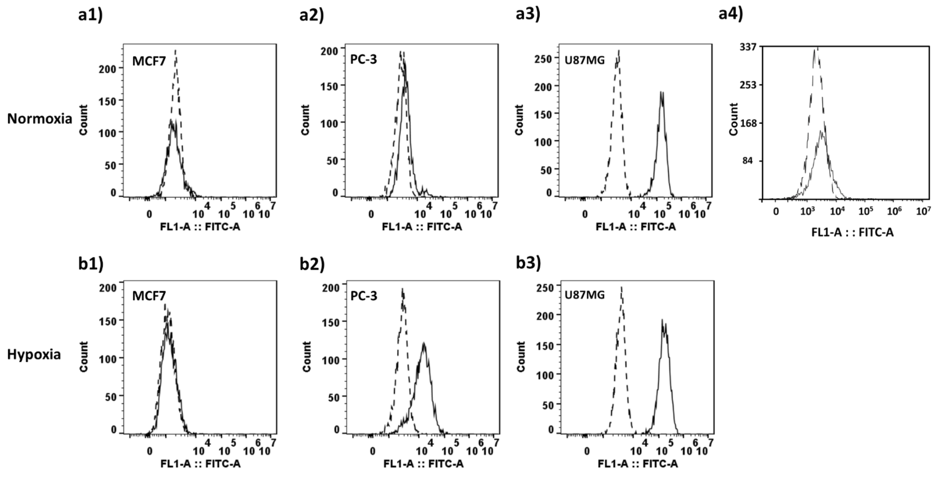

Figure S29. (a) Representative flow cytometry histogram of integrin $a_V\beta_3$ expression of MCF7 (1), PC-3 (2), U87MG (3) and U87MG-kd (4) cells cultured under normoxia (21% O_2, a) and hypoxia (1% O_2, b).

Solid lines represent the fluorescence intensity of the cells after the incubation with anti-integrin $a_V\beta_3$ first antibody followed by Alexa Fluor™ 488 conjugated goat anti-mouse IgG second antibody. Dotted lines indicate the control group in which the cells were stained only by the secondary antibody.

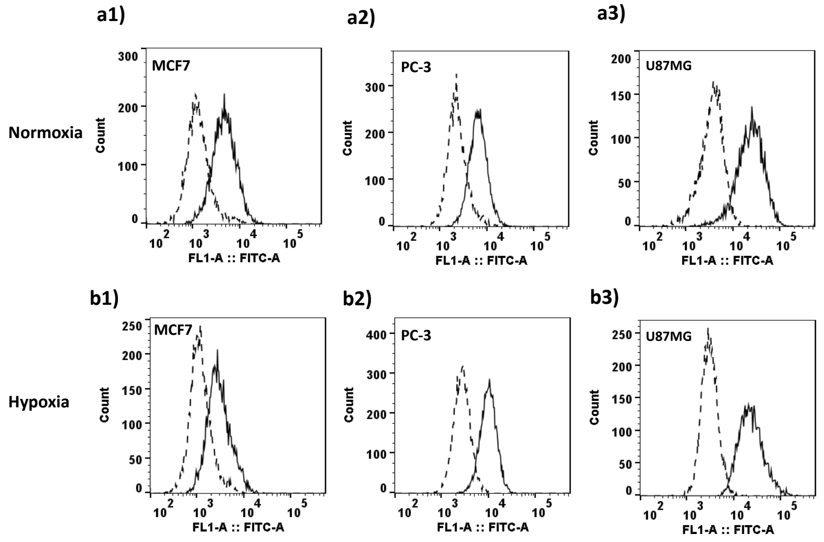

Figure S30. a) Representative flow cytometry histogram of integrin $a_V\beta_5$ expression of MCF7 (1), PC-3 (2) and U87MG (3) cells cultured under normoxia (21% O_2, a) and hypoxia (1% O_2, b). Solid lines represent the fluorescence intensity of the cells after the incubation with anti-integrin $a_V\beta_3$ first antibody followed by Alexa Fluor™ 488 conjugated goat anti-mouse IgG second antibody. Dotted lines indicate the control group in which the cells were stained only by the secondary antibody.

10 Cellular uptake study by ICP-MS

Table S4. Ru accumulation (μg Ru/million cells) for U87MG, PC-3 and MCF7 cells 6 h after exposure to Λ-[**1**]Cl_2 or Δ-[**1**]Cl_2 (12.5 μM, dark) under normoxia and hypoxia. Each experiment was conducted in triplicate wells (technical replicates).

Compounds	U87MG (norm)	PC-3 (norm)	MCF7 (norm)	U87MG (hypo)	PC-3 (hypo)	MCF7 (hypo)
Λ-[**1**]Cl_2	0.74 ± 0.01	0.55 ± 0.03	0.25 ± 0.01	0.84 ± 0.01	0.68 ± 0.01	0.31 ± 0.01
Δ-[**1**]Cl_2	0.73 ± 0.01	0.51 ± 0.01	0.21 ± 0.01	0.78 ± 0.01	0.63 ± 0.02	0.31 ± 0.01

11 Protein interaction study

Table S5. Concentration of protein and of Λ-[**1**]Cl_2, Δ-[**1**]Cl_2 or Δ-[**3**]Cl_2 during the titration monitored by emission spectrometer. [a]

No.	Concentration of protein (μM)	Concentration of complexes (μM) [b]
**0**	0.1	0
**1**	0.1	0.01
**2**	0.1	0.02
**3**	0.1	0.04
**4**	0.1	0.06
**5**	0.1	0.08
**6**	0.1	0.1

[a] The concentration of protein and complexes stock solution were 4.2 μM and 1 mM respectively, and the working solution of protein and complexes were 0.1 μM and 10 μM. [b] Final concentration was corrected because of volume dilution.

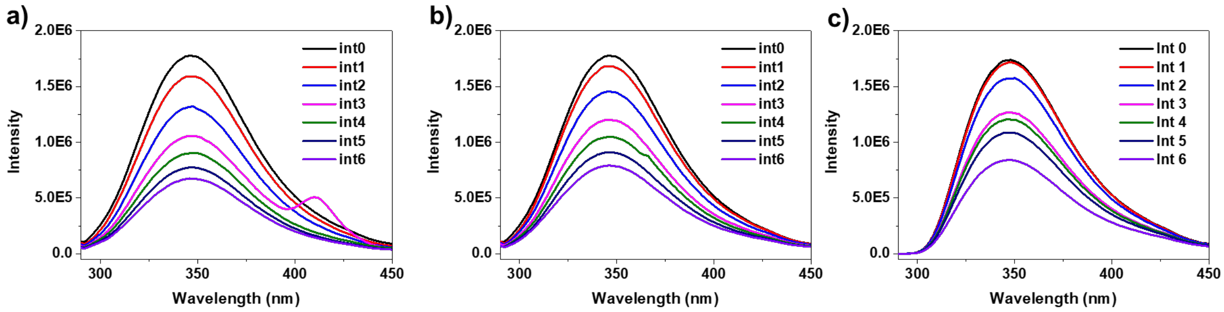

Figure S31. The emission spectrum of integrin, $\alpha_{IIb}\beta_3$ (0.1 μM in TrisHCl buffer; λ_{ex} = 280 nm; λ_{em} = 345 nm) in the presence of increasing amounts of complexes Λ-[**1**]Cl_2 (a), Δ-[**1**]Cl_2 (b), Δ-[**3**]Cl_2 (c). Stock concentration of protein and complexes were 4.2 μM and 1 mM. Complex working solution (10 μM), dissolved in TrisHCl buffer (20 mM trisHCl, 150 mM NaCl, 1 mM $CaCl_2$) was added sequentially to 0.1 μM integrin in the same buffer to generate the final working concentration as Table S5, the emission spectrum was monitored accordingly. Corrections for dilution were applied for all recorded spectra.

12 Lipophilicity (log P) study

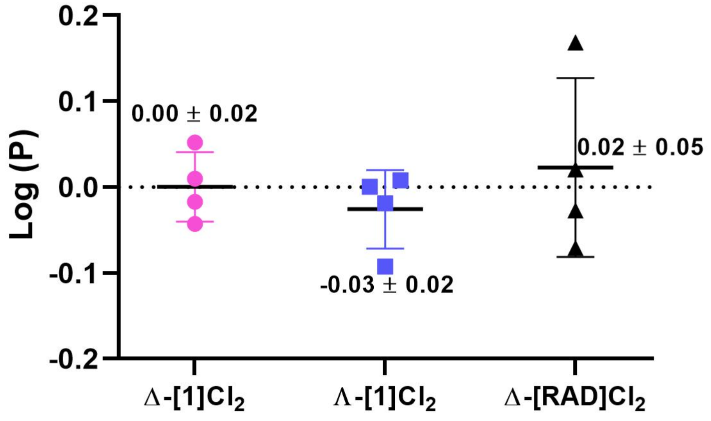

Figure S32. Octanol-water partition coefficients (Log P) of Λ-[**1**]Cl_2, Δ-[**1**]Cl_2 and Δ-[**3**]Cl_2. Errors were calculated as standard derivation from 4 concentration points (see experimental part).

13 Cytotoxicity study including a washing step before irradiation: dose-response curves

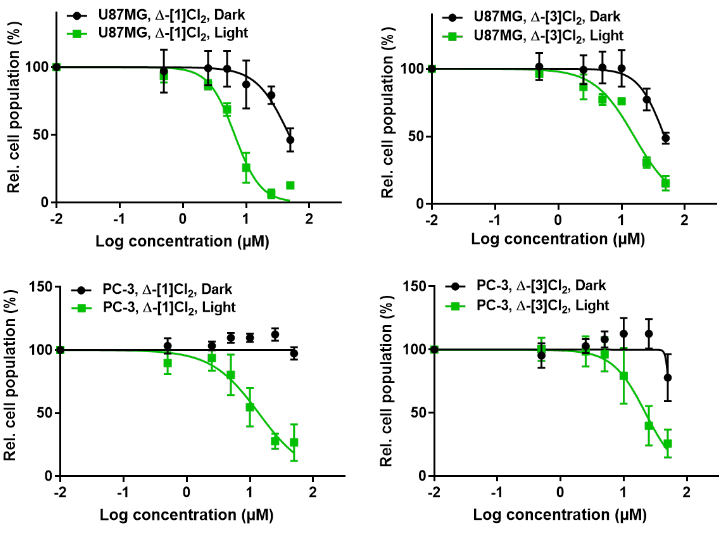

Figure S33. Dose-response curves for U87MG and PC-3 cell lines incubated with Δ-[**1**]Cl_2, or Δ-[**3**]Cl_2 for 6 h, washed with fresh medium, then either left in the dark or irradiated with green light (520 nm, 13.1 J/cm^2), and finally incubated in the dark for another 48 h. End point cell viability assay: SRB (at t = 96 h). Conditions: 37°C, 21% O_2 and 5% CO_2. Every group was conducted in triplicate, error bars represent 95% confidence intervals.

Table S6. Half-maximal effective concentrations (EC_{50} in µM) and 95% confidence intervals (CI_{95} in µM) in U87MG and PC-3 cells treated with Δ-[**1**]Cl_2 or Δ-[**3**]Cl_2 for 6 h and washed with drug-free medium before light irradiation (520 nm, 13.1 J/cm^2).

Complex	EC50 (µM)[a]						
	U87MG				PC-3		
	dark		light		dark	light	
Δ-[1]Cl_2	47.8	-9.2 +21.4	6.6	-0.7 +0.8	>50	13.8	-3.4 +5.0
Δ-[3]Cl_2	48.0	-7.2 +12.6	15.8	-2.4 +2.7	>50	22.1	-4.4 +5.8

14 *In vivo* antitumor study

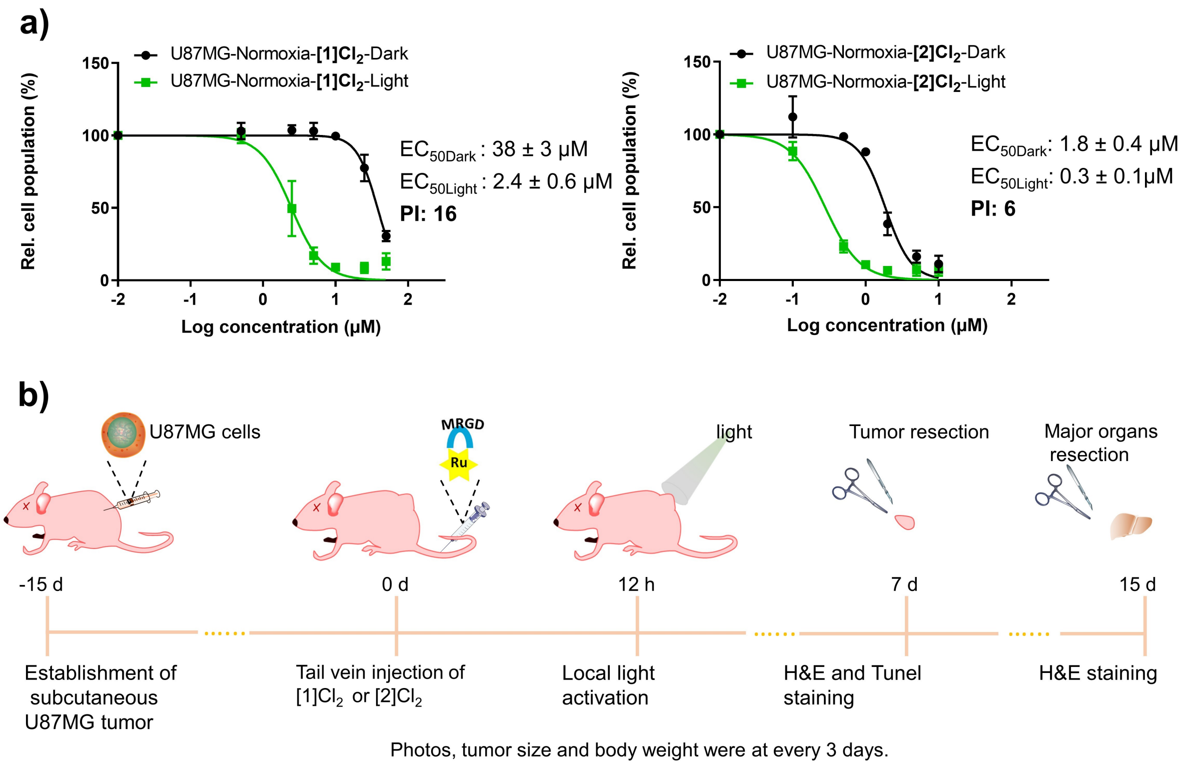

Figure S34. (a) Dose-response curves of 2D monolayers U87MG cells incubated with complex [**1**]Cl_2 and [**2**]Cl_2 (both as mixture of Δ and Λ) in the dark (black) or irradiated with green light (green, 520 nm, 13.1 J/cm^2). (b) Schematic illustration of the schedule for tumor therapy.

Table S7. Biodistribution of Ru content (%ID/g, n = 3) in major organs of mice at different time points following intravenous injection of [**1**]Cl_2 (7.7 mg/kg). or [**2**]Cl_2 (5 mg/kg). %ID/g = Ru content (μg) /tissue (g) compare to total injection Ru (μg).

Time	Complex	Heart	Liver	Spleen	Lung	Tumor	Kidney
2 h	[**1**]Cl_2	2.2 ± 0.4%	40.2 ± 2.8%	1.9 ± 0.1%	1.5 ± 0.2%	2.0 ± 0.5%	3.2 ± 0.3%
	[**2**]Cl_2	2.0 ± 0.3%	40.5 ± 3.3%	4.6 ± 0.4%	1.5 ± 0.3%	1.8 ± 0.3%	3.2 ± 0.4%
6 h	[**1**]Cl_2	1.8 ± 0.3%	29.0 ± 1.6%	2.1 ± 0.5%	1.6 ± 0.2%	7.3 ± 0.4%	2.0 ± 0.2%
	[**2**]Cl_2	1.9 ± 0.5%	29.5 ± 1.9%	2.2 ± 0.5%	1.6 ± 0.2%	6.5 ± 0.5%	2.0 ± 0.4%
12 h	[**1**]Cl_2	1.8 ± 0.2%	16.5 ± 3.5%	2.2 ± 0.4%	1.6 ± 0.2%	15.7 ± 1.3%	1.7 ± 0.3%
	[**2**]Cl_2	1.8 ± 0.3%	16.3 ± 3.3%	2.2 ± 0.3%	1.7 ± 0.2%	12.3 ± 1.5%	1.7 ± 0.2%
18 h	[**1**]Cl_2	1.9 ± 0.2%	14.4 ± 4.3%	1.9 ± 0.3%	1.8 ± 0.3%	7.2 ± 0.5%	1.9 ± 0.2%
	[**2**]Cl_2	1.7 ± 0.2%	14.6 ± 2.3%	1.6 ± 0.2%	1.7 ± 0.1%	2.8 ± 0.6%	1.9 ± 0.2%
24 h	[**1**]Cl_2	1.8 ± 0.1%	6.1 ± 1.2%	1.7 ± 0.2%	1.7 ± 0.2%	4.1 ± 1.0%	1.8 ± 0.2%
	[**2**]Cl_2	1.7 ± 0.2%	7.3 ± 0.8%	1.7 ± 0.2%	1.7 ± 0.1%	1.7 ± 0.1%	1.5 ± 0.1%

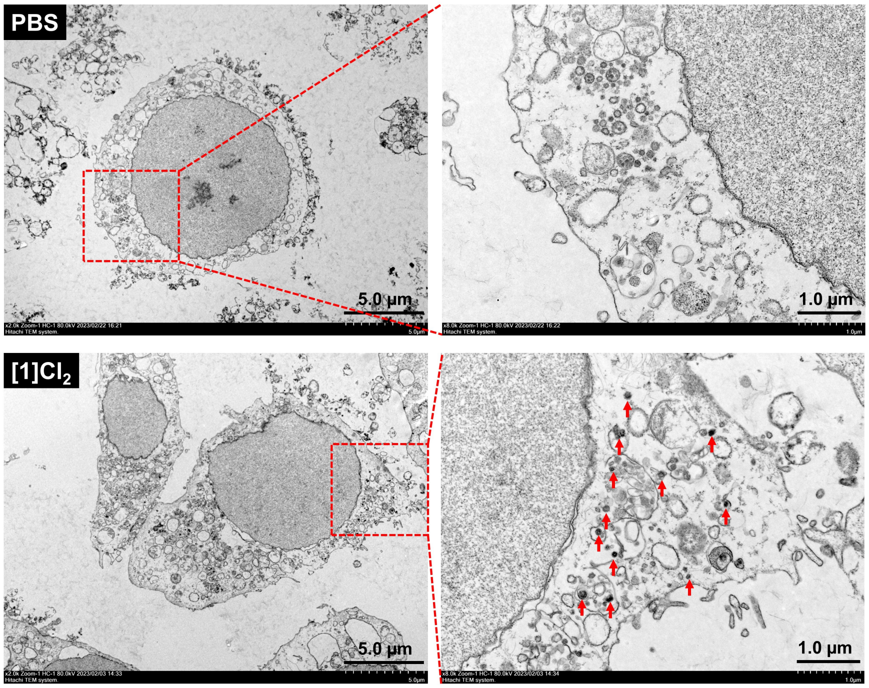

Figure S35. EM images of U87MG cells after 12 h post-injection of PBS 1X (100 μL) or [**1**]Cl_2 (7.7 mg/kg) (100 μL RMPI 1640 medium).

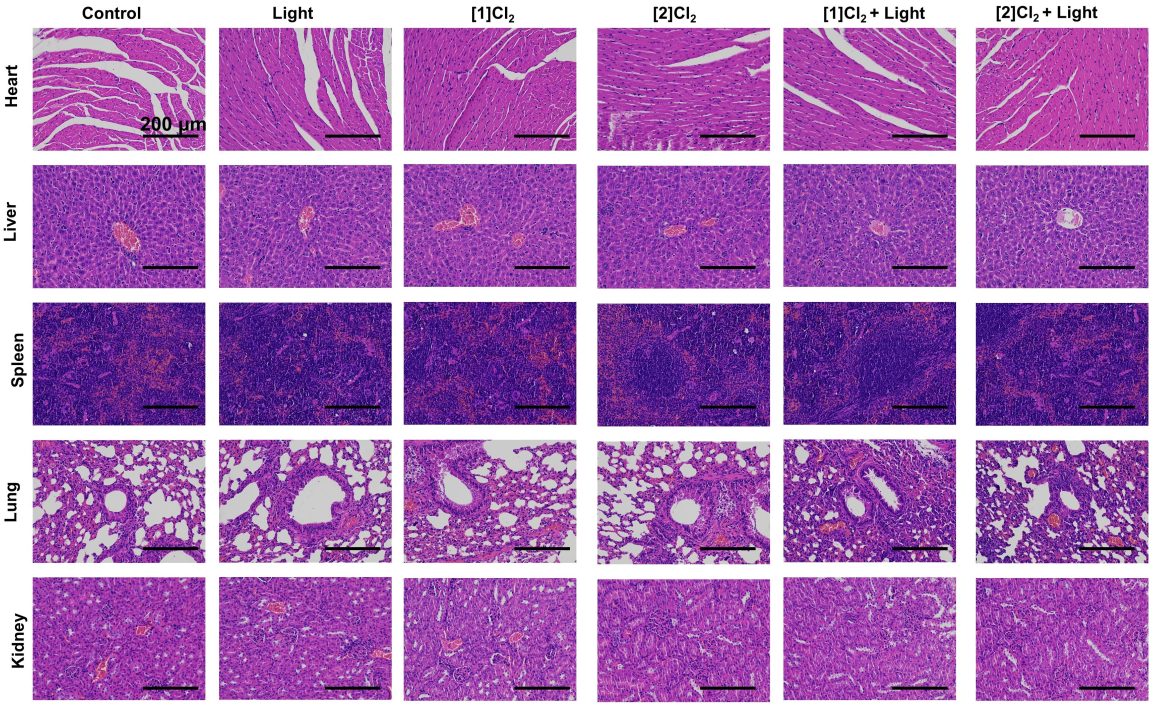

Figure S36. H&E stained images of major organs resected from of U87MG tumor-bearing mice after different treatments at day 15.

Reference

1. J.-A. Cuello-Garibo, M. S. Meijer and S. Bonnet, *Chemical Communications*, 2017, **53**, 6768-6771.
2. M. C. DeRosa and R. J. Crutchley, *Coordination Chemistry Reviews*, 2002, **233**, 351-371.
